# The prevalence and role of human respiratory syncytial virus in pediatric respiratory tract infections: a systematic review and meta-analysis of global data

**DOI:** 10.1016/j.eclinm.2026.103837

**Published:** 2026-03-20

**Authors:** Pegah Khales, Mohammad Hossein Razizadeh, Saied Ghorbani, Hassan Saadati, Zahra Salavatiha, Afagh Moattari, Ahmad Tavakoli

**Affiliations:** a Department of Virology, School of Medicine, Shiraz University of Medical Sciences, Shiraz, Iran; b Division of Microbiology and Infection, University of Leicester, Leicester, United Kingdom; c Addiction and Behavioral Sciences Research Center & Department of Epidemiology and Biostatistics, School of Health, North Khorasan University of Medical Sciences, Bojnurd, Iran; d Department of Virology, School of Medicine, Iran University of Medical Sciences, Tehran, Iran; e Biological Products and Blood Safety Research Center, High Institute for Research and Education in Transfusion Medicine, Tehran, Iran

**Keywords:** Human respiratory syncytial virus, Pediatric respiratory infections, Global prevalence, hRSV, Meta-analysis, Respiratory tract infections

## Abstract

**Background:**

Human respiratory syncytial virus (hRSV) is a major cause of respiratory tract infections in children worldwide. This study aims to describe the prevalence of hRSV in pediatric patients with respiratory tract infections, clarifying its association with such infections.

**Methods:**

We analyzed studies from PubMed, Scopus, and Web of Science up to August 15, 2025, focusing on polymerase chain reaction-confirmed cases in children under 18 years. Data from 539 studies (584 datasets) were included. Pooled prevalence was calculated using a random-effects model, with subgroup analyses by region, gender, age group, sampling time, type of respiratory disease, types of patient care, genotypes, and subtypes of hRSV. Odds ratios evaluated the association between hRSV infection and respiratory disease risk.

**Findings:**

The global prevalence among 1,733,341 children was 21.6%, with the highest rates in children aged less than 6 months (33.8%), and inpatients (25.9%). Bronchiolitis showed the highest prevalence (56.9%). Prevalence declined over time, possibly due to the coronavirus disease 2019 pandemic. hRSV-A (55.7%) was more common than hRSV-B (44.3%). Infection significantly increased respiratory infection risk (odds ratio = 7.0), especially for lower respiratory infections.

**Interpretation:**

hRSV is a key contributor to pediatric respiratory tract infections, with notable variations by age and region. Prevention strategies, including vaccines and monoclonal antibodies, are urgently needed for high-risk groups.

**Funding:**

None.

**Research in context**

**Evidence before this study**

Prior to this study, research on human respiratory syncytial virus (hRSV) in pediatric respiratory tract infections (RTIs) had been conducted extensively, but existing studies were often limited by regional focus, small sample sizes, or methodological inconsistencies. To systematically evaluate the evidence, we performed a comprehensive search of the published literature. Previous findings indicated that hRSV is a leading cause of pediatric RTIs. However, the lack of a unified global analysis, combined with heterogeneity in study designs and seasonal variations, hindered the generalizability of these results.

**Added value of this study**

This study advances the field by presenting the first global systematic review and meta-analysis of hRSV prevalence in pediatric RTIs, integrating data from 539 studies across the world. Its contributions include an unprecedented geographic scope that shows disparities in prevalence. By restricting inclusion to PCR-confirmed cases, the study minimized diagnostic variability and improved comparability across datasets. Additionally, it identified a notable decline in hRSV prevalence after 2020, apparently attributable to the impact of COVID-19-related public health measures.

The study also quantified the strong association between hRSV infection and severe respiratory outcomes. By analyzing subgroups such as age, patient type (inpatient vs. outpatient), and respiratory conditions (e.g., bronchiolitis, pneumonia), this work provides nuanced insights that were previously unavailable in literature.

**Implications of all the available evidence**

The results show hRSV as a major contributor to pediatric respiratory morbidity, particularly among infants and hospitalized children. These findings have critical implications for public health policies and clinical practice. The high disease burden in low- and middle-income countries (LMICs) highlights the urgent need for accessible prevention strategies, including vaccines and monoclonal antibodies.

## Introduction

Respiratory tract infections (RTIs) are a leading cause of morbidity and mortality among children worldwide, particularly in low- and middle-income countries (LMICs).^1^ Among the various pathogens responsible for RTIs, human respiratory syncytial virus (hRSV) stands out as one of the most significant contributors to pediatric respiratory illness.^2^ hRSV is a major cause of bronchiolitis, pneumonia, and other lower respiratory tract infections (LRTIs) in infants and young children, often leading to hospitalization and, in severe cases, death.^3^ Despite its global impact, the burden of hRSV-associated RTIs remains poorly quantified in many regions, particularly in resource-limited settings where diagnostic capabilities and surveillance systems are often inadequate.^4^

The epidemiology of hRSV is characterized by seasonal outbreaks, typically occurring during colder months in temperate climates and during the rainy season in tropical regions.^5^ However, the timing and intensity of these outbreaks can vary significantly across different geographic areas, complicating efforts to implement targeted prevention and control strategies. Furthermore, while hRSV is recognized as a major cause of pediatric RTIs, the prevalence of hRSV among children with RTIs has not been systematically synthesized on a global scale.^6^ Existing studies often focus on specific regions or populations, limiting the generalizability of their findings.^7^

Understanding the global prevalence of hRSV among children with RTIs is critical for informing public health interventions, including vaccine development and deployment, as well as for guiding resource allocation in healthcare systems. Recent advances in hRSV vaccine candidates and monoclonal antibodies have highlighted the urgent need for accurate, up-to-date data on hRSV epidemiology to support their effective implementation.^8^^,^^9^ Despite this, a comprehensive synthesis of hRSV prevalence data across diverse geographic and demographic settings is lacking.

This systematic review and meta-analysis aims to address this gap by providing a comprehensive estimate of the worldwide prevalence of hRSV among children with RTIs. By synthesizing data from studies conducted across different regions and populations, this work will provide a clearer picture of the global burden of hRSV-associated RTIs in children. The novelty of this study lies in its global scope, encompassing data from both high-income countries (HICs) and LMICs, and its focus on the pediatric population, which is disproportionately affected by hRSV. Furthermore, this study will explore variations in hRSV prevalence by different variables such as geographic region and age group, offering insights that can inform targeted prevention and treatment strategies. The findings of this study are expected to contribute significantly to the global understanding of hRSV epidemiology and to support efforts to reduce the burden of hRSV-associated RTIs in children worldwide.

## Methods

The Preferred Reporting Items for Systematic Reviews and Meta-Analyses (PRISMA) guideline served as the foundation for this systematic review and meta-analysis approach.^10^

### Search strategy

To identify relevant studies, a comprehensive literature search was conducted across three electronic databases: PubMed, Scopus, and Web of Science. The search was limited to studies published from the inception of each database up to August 15, 2025. The specific search terms used for each database are detailed in Supplementary Table S1. Additionally, the reference lists of relevant articles were manually reviewed to identify further studies that met the inclusion criteria. For efficient data organization, the results of the systematic literature search were imported into EndNote software version ×8 (Thomson Reuters, California, USA).

### Selection criteria

Studies were considered qualified if they reported: (1) studies providing data related to the prevalence of hRSV among children less than 18 years with respiratory symptoms published in the English language in peer-reviewed journals; (2) the prevalence of hRSV genome in respiratory samples; (3) studies detecting hRSV genome by polymerase chain reaction (PCR)-based methods; (4) studies detecting the prevalence of hRSV among inpatients and outpatients; (5) original articles and short communications with sufficient data.

Notably, for prevalence analysis, we also included case–control studies that reported the number of laboratory-confirmed RSV cases among children with clinically suspected respiratory tract infection. For case–control studies, only data from the symptomatic “case” group were extracted. In these studies, the case group consisted of pediatric patients presenting with respiratory tract infection who underwent virological testing, and RSV status was determined using standard laboratory methods (PCR). Because the denominator comprised all tested symptomatic children within the defined study period, the proportion of RSV-positive cases in the case group is epidemiologically equivalent to period prevalence (detection rate) in a diagnostic cross-sectional cohort.

Studies that met any of the following criteria were excluded:

(1) The prevalence of hRSV infection among adult patients with respiratory symptoms. hRSV epidemiology, clinical presentation, and risk factors differ substantially between children and adults. Including adults would introduce unacceptable clinical and immunological heterogeneity.
(2) The prevalence of hRSV infection among children with underlying conditions such as cancer, cystic fibrosis, asthma, chronic obstructive pulmonary disease (COPD), chronic heart diseases, chronic neurological disease, acute otitis media, HIV, Kawasaki disease, immunocompromised status, transplant recipients, and down syndrome. These conditions markedly increase susceptibility to hRSV and severity of disease, leading to substantially higher detection rates that do not reflect the burden in the general pediatric population.
(3) Samples other than respiratory specimens such as blood. hRSV is primarily a respiratory pathogen; detection in blood usually reflects severe disseminated disease in immunocompromised patients rather than typical respiratory infection.
(4) Detection of hRSV by assays other than PCR-based methods such as ELISA, immunofluorescence, reverse transcription loop-mediated isothermal amplification (RT-LAMP), shell vial culture, flow cytometry system, complement fixation test, virus isolation, antigen detection, enzyme immunoassay, and immunochromatographic test. Older non-molecular methods have significantly lower and variable sensitivity/specificity compared with PCR, especially in older studies. Restricting to PCR-confirmed cases markedly reduces diagnostic misclassification bias and improves comparability across three decades of studies.
(5) Seroprevalence of hRSV antibodies. Antibody-based studies measure past exposure rather than acute infection and cannot be pooled with virological prevalence data.
(6) Studies including patients with non-respiratory symptoms. hRSV detection in asymptomatic or non-respiratory contexts does not contribute to understanding its role in acute respiratory illness.
(7) The prevalence of a specific genotype or variant of hRSV. Such studies often selectively test or report only certain lineages, biasing prevalence estimates.
(8) Letters, case series, notes, comments, reviews, case reports, posters, and conference abstracts. These typically lack sufficient methodological detail and raw data for reliable quality assessment and extraction.
(9) Articles published in languages other than English. Although this may introduce language bias, thorough extraction and quality assessment by the review team would not have been feasible without full comprehension of the original text.

### Data extraction and quality assessment

Three reviewers independently screened the titles and abstracts of all identified studies, removing those that were irrelevant to the research topic. The full texts of the remaining papers were retrieved and further evaluated by the reviewers, with studies failing to meet the inclusion criteria being excluded. Any disagreements among the reviewers were resolved through discussion with a fourth reviewer. To assess the quality of the included studies, a modified version of the Strengthening the Reporting of Observational Studies in Epidemiology (STROBE) checklist was utilized.^11^^,^^12^ The checklist included 12 questions designed to evaluate various methodological aspects of the studies. Only those studies that achieved a validity score of 8 or higher out of a maximum possible score of 12 were deemed eligible for inclusion in the main meta-analysis. Three reviewers extracted the data listed below from each eligible article: first author's last name, year of publication, year of sampling, study location, study design, sample size, type of sample, age ranges of patients, age groups of patients, the gender of patients, number of hRSV-positive cases, types of patient care, type of respiratory disease, and subtypes of hRSV. The retrieved data were entered into a pre-designed Excel spreadsheet (Microsoft Corporation, Redmond, Washington, USA).

### Statistical analysis

Prevalence in this meta-analysis was defined as the proportion of children with clinically suspected acute respiratory tract infection (i.e., presenting with respiratory symptoms that prompted virological testing) who tested positive for hRSV by PCR-based methods in respiratory specimens. This corresponds to the detection rate of hRSV among symptomatic pediatric patients tested in the included studies and is the standard measure used in the vast majority of diagnostic prevalence studies of respiratory viruses. We pooled the hRSV infection in children suffering from respiratory diseases using the metaprop package.^13^ We applied the random-effects meta-analysis framework and subgroup analysis was conducted based on region, gender, age group, sampling time, type of respiratory disease, types of patient care, genotypes, and subtypes of RSV. We also conducted meta-analyses of risk estimates for respiratory diseases and exposure to hRSV, and we reported pooled estimates of odds ratio (OR) and 95% CIs. DerSimonian and Laird method^14^ was used to compute the pooled estimate of OR with confidence interval (95% CI) using random models. To calculate prevalence during the peak season and throughout the year, studies were categorized according to surveillance duration as (1) peak-season studies, defined as those conducted during a defined RSV epidemic or high-transmission season only, and (2) year-round studies, defined as those conducting continuous surveillance for ≥12 months. Statistical heterogeneity between studies was evaluated with Cochran's Q test and quantified by I^2^ statistic.^15^ We investigated the presence and the effect of publication bias using a combination of the visual inspection of funnel plots that were constructed, plotting the logarithmically transformed ORs against the standard error of the associated log (OR) and Begg's test and Egger's test. Subgroup differences were evaluated using the random-effects subgroup analysis. For each subgroup, a pooled prevalence was estimated and statistical differences between subgroups were tested using the Q-test for heterogeneity between groups (Q_between). A *P*-value <0.05 was considered statistically significant. All statistical tests were two-tailed and the significance level was considered less than 0.05 for all, except heterogeneity test that were set at less than 0.1, and statistical analyses were performed using Stata 14.1 (Stata Corp, College Station, TX, USA).

### Ethics statement

Ethics statement was not required for this study.

### Statement on informed consent

Informed consent was not required.

### Role of the funding source

There was no funding source for this study.

## Results

### Literature search

**Fig. 1:**
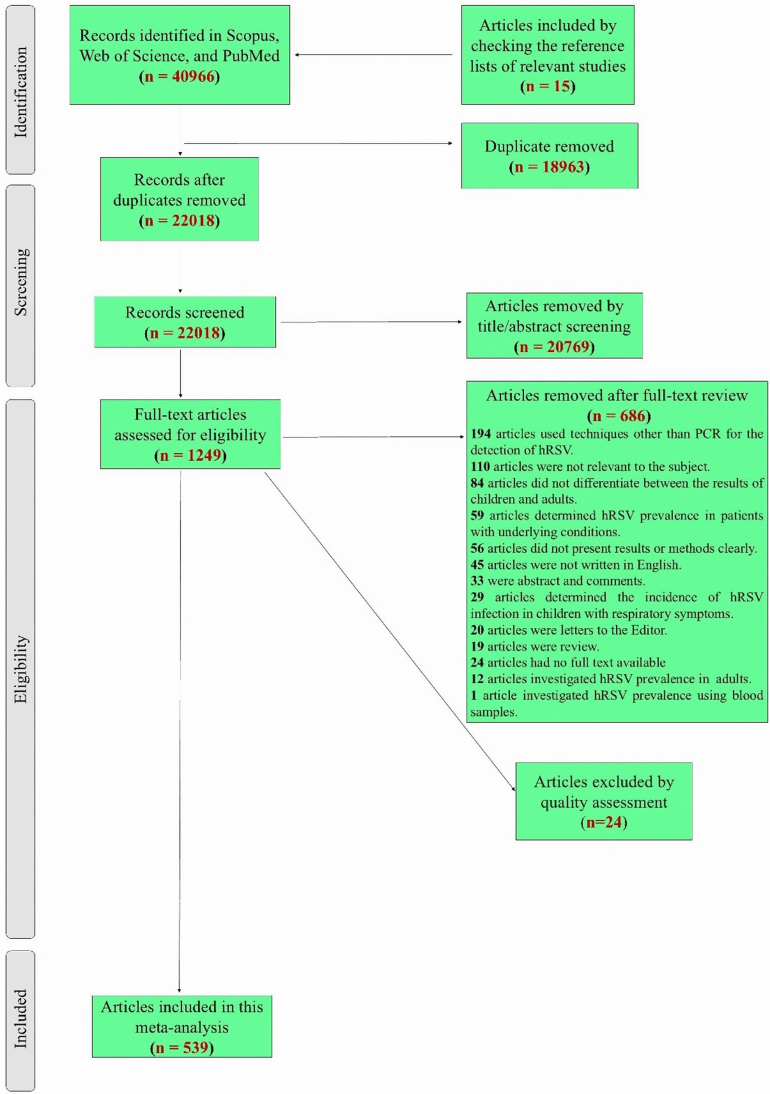
Flowchart presenting the steps of literature search and selection.

During the initial search, 40,966 papers were identified, and 15 further papers were discovered by manually examining the reference lists of pertinent research. A total of 18,963 duplicate papers were initially removed, and 20,769 additional papers were removed after a manual check of titles and abstracts. After a thorough evaluation of the full text of the remaining 1249 papers to determine their eligibility for the meta-analysis, 686 of them were removed. According to the modified STROBE checklist, 539 publications were deemed to be of good quality (scoring 8 or higher), with 24 papers failing to get a score of 8. Finally, this systematic review and meta-analysis contained 539 papers (584 datasets). An overview of the selection of relevant studies is depicted in Fig. 1.

### Study characteristics

Out of the 584 research, 551 studies were cross-sectional and 33 studies were case–control in design. The articles' publication dates varied from 1992 to 2025. The largest research involved 155,165 pediatric patients with respiratory infections,^16^ while the smallest contained 11 cases.^17^ Out of the 584 datasets included in this meta-analysis, 73 studies examined the gender distribution of hRSV infection, and 144 studies performed hRSV typing. Overall, children under 5 years of age (n = 688,375) were the predominant population participating in the studies compared to children aged 6–18 years (n = 107,328). Among the age group of children under 5 years old, the largest population included in the studies were children under 6 months of age (n = 68,866). The country with the highest number of studies was China (n = 120), followed by India (n = 32), United States (n = 28), and Italy (n = 24). In terms of the number of participants, out of 1,733,341 cases, China ranked first with 834,429 cases, followed by Brazil, Italy, Spain, and the United States with 128,602, 89,204, 88,651, and 71,365 cases, respectively. The characteristics of included studies in this systematic review and meta-analysis are summarized in Table 1. Table 2 shows results of subgroup analysis of the prevalence of hRSV infection in children with respiratory infections. Results of the meta-regression analysis are presented in the Supplementary File.

**Table 1:** Characteristics of all studies included in this systematic review and meta-analysis.

Author (Ref)	Publication year	Location	Type of disease	Number of cases	No. RSV positive cases
Cubie^18^	1992	UK	Bronchiolitis	123	45
Freymuth^19^	1997	France		277	173
Grondahl^20^	1999	Germany		1031	141
Weigl^21^	2000	Germany		1281	162
Zambon^22^	2001	UK	ILI	762	198
Weigl^23^	2002	Germany		2108	348
Cuevas^24^	2003	Brazil	LRI	111	61
Jartti^25^	2004	Netherlands	Wheezing	291	50
Jennings^26^	2004	New Zealand		75	32
Scott^27^	2004	Kenya		1044	397
Serafino^28^	2004	Brazil	LRI	217	133
Al-Sonboli^29^	2005	Yemen		604	244
Kotaniemi-syrjanen^30^	2005	Finland	Wheezing	61	14
Mentel^31^	2005	Germany		356	94
Sato^32^	2005	Japan	LRI	499	185
Versteegh^33^	2005	Netherlands		136	1
Al-Sonboli^34^	2006	Yemen		601	266
Choi^35^	2006	South Korea	LRI	515	122
Crowcroft^36^	2007	UK		91	70
Naghipour^37^	2007	Iran		261	39
Pierangeli^38^	2007	Italy		227	39
Teeratakulpisarn^39^	2007	Thailand	Bronchiolitis	170	110
Thomazelli^40^	2007	Brazil	LRI	336	81
Alper^41^	2008	USA	URI	170	29
Bonzel^42^	2008	Germany		254	112
Bosis^43^	2008	Italy	Wheezing	85	63
Calvo^44^	2008	Spain	LRI	749	376
Kaplan^45^	2008	Jordan		326	140
Rihkanen^46^	2008	Finland	Wheezing	76	21
Rihkanen^46^	2008	Finland		144	21
Agrawal^47^	2009	India		1720	177
Al-Majhdi^48^	2009	Saudi Arabia		200	70
Bharaj^49^	2009	India	LRI	301	61
Chun^50^	2009	South Korea	LRI	297	87
Fabbiani^51^	2009	Italy	URI	166	7
Fabbiani^51^	2009	Italy	LRI	71	27
Hall^52^	2009	USA		2892	547
Midulla^53^	2009	Italy	Bronchiolitis	182	75
Pavlova^54^	2009	Bulgaria		278	67
Sung^55^	2009	China		475	40
Zaraket^56^	2009	Lebanon	ILI	24	10
Calvo^57^	2010	Spain	Bronchiolitis	318	195
Antunes^58^	2010	Portugal	Bronchiolitis	207	166
Faghihloo^59^	2010	Iran		107	24
Garcıa-Garcıa^60^	2010	Spain	Wheezing	626	170
Malekshahi^61^	2010	Iran	ILI	202	34
Nascimento^62^	2010	Brazil	Bronchiolitis	77	49
Singleton^63^	2010	USA	LRI	440	102
Wang^64^	2010	China		817	120
Zhang^65^	2010	China		894	341
Zhang^66^	2010	China		1387	439
Pientong^67^	2011	Thailand	Bronchiolitis	170	110
Bezerra^68^	2011	Brazil		407	152
Do^69^	2011	Vietnam		309	73
Frobert^70^	2011	France		73	46
Fujitsuka^71^	2011	Japan	Wheezing	115	61
Gardinassi^72^	2011	Brazil		272	79
Jin^73^	2011	China	LRI	813	331
Kristoffersen^74^	2011	Norway	LRI	536	142
Mathisen^75^	2011	Nepal	Pneumonia	627	88
Pogka^76^	2011	Greek	ILI	1272	155
Razanajatovo^77^	2011	Madagascar	ILI	177	54
Salomao Junior^78^	2011	Brazil	LRI	290	85
Sezer^79^	2011	Turkey	LRI	55	21
Simoes^80^	2011	Indonesia	LRI	802	163
Suntarattiwong^81^	2011	Thailand	LRI	354	104
Suryadevara^82^	2011	USA		197	104
Wang^83^	2011	UK		155	3
Zuccotti^84^	2011	Italy		575	196
Esposito^85^	2012	Italy	Pneumonia	592	188
Brand^86^	2012	Netherlands	Bronchiolitis	142	104
Chatzopoulou^87^	2012	Greece	ILI	430	45
Cho^88^	2012	South Korea	LRI	108	46
Garcia-Garcia^89^	2012	Spain	Pneumonia	884	270
Gorjipour^90^	2012	Iran	URI	330	17
Hoffmann^91^	2012	Madagascar		295	35
Hombrouck^92^	2012	Belgium	ILI	139	27
Kadjo^93^	2012	Ivory Coast	ILI	470	113
Kwofie^94^	2012	Ghana	LRI	128	18
Mansbach^95^	2012	USA	Bronchiolitis	2207	1589
Pierangeli^96^	2012	Italy		231	87
Schlaudecker^97^	2012	Honduras		345	26
Suzuki^98^	2012	Philippines	Pneumonia	819	198
Turner^99^	2013	Thailand	Pneumonia	640	174
Aamir^100^	2013	Pakistan		105	75
Alavi^101^	2013	Iran		100	29
Ali^102^	2013	Pakistan	Pneumonia	169	30
Bigogo^103^	2013	Kenya		5595	756
Choudhary^104^	2013	India		854	159
Enan^105^	2013	Sudan		368	26
Feikin^106^	2013	Kenya	SARI	408	90
Guerrier^107^	2013	Cambodia	LRI	1006	192
Harada^108^	2013	Japan		286	128
Harada^108^	2013	Japan	Pneumonia	86	44
Huang^109^	2013	China		279	36
Huo^110^	2013	China	SARI	511	87
Jafri^111^	2013	USA	LRI	4172	1306
Kim^112^	2013	South Korea		4212	1212
Li^113^	2013	China	ILI	844	86
Miyaji^114^	2013	Japan		214	37
Miller^115^	2013	USA	URI	175	18
Miller^115^	2013	USA	Bronchiolitis	455	298
Miller^115^	2013	USA		18	4
Nakouné^116^	2013	Central African Republic		329	10
Naorat^117^	2013	Thailand	LRI	6641	876
Nikfar^118^	2013	Iran	LRI	100	9
Ohno^119^	2013	Philippines	Pneumonia	2150	415
Tecu^120^	2013	Romania		241	49
Tran^120^	2013	Vietnam		1082	257
Zhang^121^	2013	China	SARI	370	189
Broor^122^	2014	India		245	50
Hara^123^	2014	Japan		495	138
He^124^	2014	China		2025	296
Kool^125^	2014	Netherlands		257	36
Kono^126^	2014	New Guinea	ILI	167	22
Lekana-Douki^127^	2014	Gabon	ILI	921	114
Shatizadeh^128^	2014	Iran		202	34
Al-Ayed^129^	2014	Saudi Arabia		135	33
Balmaks^130^	2014	Latvia	LRI	207	88
Cai^131^	2014	China		1980	446
Faghihloo^132^	2014	Iran		485	94
Feng^133^	2014	China	LRI	20,637	9087
Gooskens^134^	2014	Netherlands		274	69
Junior^135^	2014	Brazil		116	12
Kaida^136^	2014	Japan		1044	198
Karadag-Oncel^137^	2014	Turkey	ILI	194	16
Wen Liu^138^	2014	China	Pneumonia	2361	768
Jia Liu^139^	2014	China		2407	184
Obodai^140^	2014	Ghana	LRI	53	32
Panayiotou^141^	2014	Cyprus		391	128
Pourakbari^142^	2014	Iran	LRI	232	40
Radin^143^	2014	USA	Pneumonia	270	57
Schulert^144^	2014	USA	Pneumonia	202	38
Singh^145^	2014	India	LRI	188	40
Mendoza^146^	2015	Peru		717	116
Moattari^147^	2015	Iran		252	71
Aydemir^148^	2015	Turkey	Pneumonia	78	12
Berce^149^	2015	Slovenia	LRI	278	77
Cebey-Lopez^150^	2015	Spain	LRI	204	108
Cebey-Lopez^150^	2015	UK	LRI	97	35
Cui^151^	2015	China		1074	75
Diaz^152^	2015	Mexico		162	23
Fu^153^	2015	China	ILI	305	29
Halasa^154^	2015	Jordan		3175	1397
Lagare^155^	2015	Niger		160	56
Lee^156^	2015	Taiwan		216	18
Malasao^157^	2015	Philippines	Pneumonia	1505	423
Martinez-Roig^158^	2015	Spain		463	250
Othman^159^	2015	Egypt	LRI	127	59
Ren^160^	2015	China	LRI	3167	1035
Simusika^161^	2015	Zambia		496	114
Tuan^162^	2015	Vietnam	LRI	1117	316
Wei^163^	2015	China		3181	831
Wertheim^164^	2015	Multiple countries	ILI	525	139
Yu^165^	2015	China		1820	269
Zhang^166^	2015	China	Pneumonia	371	163
Wishaupt^167^	2016	Netherlands		241	108
Richter^168^	2016	Cyprus		424	129
Ali^169^	2016	Pakistan	Pneumonia	817	13
Amer^170^	2016	Saudi Arabia	LRI	113	14
Antón^171^	2016	Spain	ILI	3482	285
Bimouhen^172^	2016	Morocco		654	211
Chou^173^	2016	Taiwan	LRI	90	5
Cangiano^174^	2016	Italy	Bronchiolitis	723	234
Do^175^	2016	Vietnam	LRI	632	302
Dong^176^	2016	China		2819	100
Dut^177^	2016	Turkey		312	29
Faber^178^	2016	Netherlands	Bronchiolitis	100	83
Fall^179^	2016	Senegal	ILI	2803	436
Girit^180^	2016	Turkey	ILI	132	46
Goktas^181^	2016	Turkey		309	43
Gurgel^182^	2016	Brazil	LRI	507	204
Hu^183^	2016	China		1827	433
Kenmoe^184^	2016	Cameroon		347	46
Karppinen^185^	2016	Finland		2275	279
Liu^186^	2016	China		5483	729
Malhotra^187^	2016	India		155	6
Meligy^188^	2016	Egypt	Pneumonia	44	9
Meskill^189^	2016	USA		13,664	3018
Mishra^190^	2016	India		300	61
Moesker^191^	2016	Netherlands		44	6
Nyawanda^192^	2016	Kenya		3634	446
Panda^193^	2016	India		332	15
Parsania^194^	2016	Iran		158	49
Reeves^195^	2016	UK		63,827	13,034
Slovic^196^	2016	Croatia		486	388
Wang^197^	2016	China	ILI	3662	206
Arbefeville^198^	2017	USA		752	72
Abdulhaq^199^	2017	Saudi Arabia		62	5
Benet^200^	2017	Cambodia	Pneumonia	176	39
Benet^200^	2017	China	Pneumonia	39	17
Benet^200^	2017	Haiti	Pneumonia	101	26
Benet^200^	2017	India	Pneumonia	192	17
Benet^200^	2017	Madagascar	Pneumonia	80	12
Benet^200^	2017	Mali	Pneumonia	118	30
Benet^200^	2017	Mongolia	Pneumonia	108	23
Benet^200^	2017	Paraguay	Pneumonia	99	14
Avcu^201^	2017	Turkey	LRI	114	27
Bashir^202^	2017	Pakistan	LRI	155	104
Bedolla Barajas^203^	2017	Mexico	Wheezing	55	7
Bhuyan^204^	2017	Bangladesh		200	62
Brini^205^	2017	Tunisia		372	123
Dang^206^	2017	China		411	95
Fagbo^207^	2017	Saudi Arabia		2235	512
Gokce^208^	2017	Turkey	Bronchiolitis	316	127
Janahi^209^	2017	Qatar	Bronchiolitis	369	189
Jonnalagadda^210^	2017	Ecuador	Pneumonia	406	159
Kim^211^	2017	South Korea		16,842	1116
Korsun^212^	2017	Bulgaria		610	157
Lim^213^	2017	Australia	ILI	2356	622
Moe^214^	2017	Norway	LRI	1816	870
Nenna^215^	2017	Italy	Bronchiolitis	723	266
Nguyen^216^	2017	Laos		383	157
O Grady^217^	2017	Australia		817	157
Pale^218^	2017	Mozambique	SARI	424	113
Park^219^	2017	South Korea	ILI	3305	180
Petrarca^220^	2017	Italy	Bronchiolitis	486	365
Piralla^221^	2017	Italy	Pneumonia	39	2
Sahu^222^	2017	India	ILI	180	56
Saxena^223^	2017	India	ILI	325	135
Swamy^224^	2017	India		689	175
Taylor^225^	2017	Australia	ILI	111	18
Taylor^225^	2017	Brazil	ILI	710	42
Taylor^225^	2017	Colombia	ILI	584	49
Taylor^225^	2017	Costa Rica	ILI	379	16
Taylor^225^	2017	Mexico	ILI	669	51
Taylor^225^	2017	Philippines	ILI	1045	167
Taylor^225^	2017	Singapore	ILI	49	4
Taylor^225^	2017	Thailand	ILI	170	12
Thongpan^226^	2017	Thailand		3306	277
Trenholme^227^	2017	New Zealand	LRI	1645	540
Valle Mendoza^228^	2017	Peru	Pneumonia	146	35
Vieira^229^	2017	Brazil	Bronchiolitis	94	73
Wishaupt^230^	2017	Netherlands		560	291
Wollmeister^231^	2017	Brazil	Bronchiolitis	142	47
Wollmeister^231^	2017	Brazil	Bronchiolitis	172	121
Wong Chew^232^	2017	Mexico	Pneumonia	1404	332
Yan^233^	2017	China	LRI	387	205
Zheng^234^	2017	China		80	33
Swamy^235^	2018	India		997	279
Tine^236^	2018	Senegal		208	34
Appak^237^	2018	Turkey		3162	292
Assane^238^	2018	Senegal		162	26
Aykac^239^	2018	Turkey		1240	74
Bhuiyan^240^	2018	Australia	Pneumonia	230	46
Canela^241^	2018	Brazil	SARI	63	7
Chen^242^	2018	China		1764	401
Chittaganpitch^243^	2018	Thailand	ILI	5069	447
Chittaganpitch^243^	2018	Thailand	SARI	1404	196
Cieslak^244^	2018	Poland	ILI	1096	73
Cowling^245^	2018	Hong Kong	ILI	2090	103
El Baroudy^246^	2018	Egypt	ILI	132	21
Famoroti^247^	2018	South Africa		2172	316
Fillatre^248^	2018	France		3199	237
Fieldhouse^249^	2018	Malaysia	Pneumonia	95	41
Gaymard^250^	2018	France		9776	2518
Ge^251^	2018	China		2160	368
Gimferrer^252^	2018	Spain		11,412	1796
Hassan^253^	2018	Iraq		269	55
Hendaus^254^	2018	Qatar	Bronchiolitis	769	352
Hindupur^255^	2018	India		135	24
Kadjo^256^	2018	Ivory Coast	ILI	917	61
Kadjo^256^	2018	Ivory Coast	SARI	142	14
Kabego^257^	2018	Congo	URI	109	16
Kabego^257^	2018	Congo	LRI	37	15
Khalifa^258^	2018	Tunisia		515	177
Kurskaya^259^	2018	Russia		1560	358
Xuechao Li^260^	2018	China		973	104
Jin Li^261^	2018	China		775	151
Nascimento Carvalho^262^	2018	Brazil	Pneumonia	774	193
Nicholson^263^	2018	USA		104	18
Obodai^264^	2018	Ghana	LRI	552	127
Ogunsemowo^265^	2018	Nigeria		231	41
Okamoto^266^	2018	Philippines		3471	439
Rashid^267^	2018	Malaysia	LRI	102	17
Ravindranath^268^	2018	USA	SARI	218	161
Razanajatovo^269^	2018	Madagascar	SARI	747	334
Snoeck^270^	2018	Laos		245	30
Tsagarakis^271^	2018	Greece		268	26
Yu^272^	2018	China		3607	427
Mackenzie^273^	2019	Gambia	LRI	519	244
Liu^274^	2019	China		11,398	1690
Tokak^275^	2019	Turkey		997	377
Abduljabbar^276^	2019	Iraq		150	26
Alharbiaburiziza^277^	2019	Saudi Arabia	LRI	129	29
Barlotta^278^	2019	Italy	Bronchiolitis	52	40
Bekhof^279^	2019	Netherlands	Bronchiolitis	218	182
Derrar^280^	2019	Algeria	LRI	117	56
Etemadi^281^	2019	Malaysia	LRI	165	83
Halaji^282^	2019	Iran		156	56
Harun^283^	2019	Turkey		269	44
Hasegawa^284^	2019	USA	Bronchiolitis	2912	2228
Hatem^285^	2019	Egypt	SARI	2479	470
Hindupur^286^	2019	India		267	57
Knobbe^287^	2019	Senegal		102	17
Korsun^288^	2019	Bulgaria	LRI	515	193
Lagare^289^	2019	Niger		638	149
Le Wang^290^	2019	China	LRI	440	124
Li^291^	2019	China	LRI	659	75
McCallum^292^	2019	Australia		794	17
McCallum^292^	2019	Australia	Bronchiolitis	333	156
Midulla^293^	2019	Italy	Bronchiolitis	998	413
Rha^294^	2019	South Africa	LRI	9969	2723
Saez Lopez^295^	2019	Portugal	ILI	756	31
Sonawane^296^	2019	India	LRI	100	29
Thongpan^297^	2019	Thailand	ILI	5081	763
Wen^298^	2019	China	LRI	3232	930
Toh^299^	2019	Malaysia	Pneumonia	439	118
Wilson^300^	2019	Ghana	SARI	2176	248
Xu^301^	2019	China	ILI	1992	124
Yen^302^	2019	Taiwan		442	88
Yew^303^	2019	Malaysia		394	85
Yurtseven^304^	2019	Turkey	Bronchiolitis	241	108
Zhao^305^	2019	China	SARI	700	198
Vanderburg^306^	2020	Sri Lanka	SARI	325	93
Tsou^307^	2020	USA	Bronchiolitis	270	179
Thongpan^308^	2020	Thailand	ILI	8209	1082
Şık^309^	2020	Turkey	LRI	123	36
Pham^310^	2020	Vietnam	LRI	194	73
Perales^311^	2020	Bolivia	Pneumonia	274	60
Palani^312^	2020	India		292	32
Lin^313^	2020	Taiwan		474	113
Lee^314^	2020	South Korea	Pneumonia	30,994	6304
Korsun^315^	2020	Bulgaria		875	229
Karaarslan^316^	2020	Turkey		88	42
Jarju^317^	2020	Gambia	ILI	735	108
Huang^318^	2020	China		14,482	2200
Hattoufi^319^	2020	Morocco	Pneumonia	86	46
Gao^320^	2020	China		3121	230
Emanuels^321^	2020	Nepal		3646	214
Duyu^322^	2020	Turkey	LRI	63	23
Chowdhury^323^	2020	Bangladesh	Pneumonia	359	32
Castro^324^	2020	Brazil		164	5
Calvo^325^	2020	Spain		5131	1607
Bunthi^326^	2020	Thailand	Pneumonia	223	51
Aygün^327^	2020	Turkey	LRI	422	103
Ang^328^	2020	Singapore		4470	375
Al-Romaihi^329^	2020	Qatar	ILI	30,946	6102
Adema^330^	2020	Kenya		1726	11
Abinaya^331^	2020	India	LRI	69	15
Atay^332^	2020	Turkey	Bronchiolitis	101	22
Aamir^333^	2020	Pakistan		1941	472
Tsergouli^334^	2020	Greece	Bronchiolitis	71	37
Luo^335^	2020	China		9158	1432
Hasuwa^336^	2020	Japan	LRI	373	87
Zhu^337^	2021	China	Pneumonia	2721	413
Vianna^338^	2021	Brazil	SARI	632	352
Vasconcelos^339^	2021	Multiple countries		349	74
Thongpan^340^	2021	Thailand	ILI	574	232
Tavakoli^341^	2021	Iran		206	74
Shutes^342^	2021	USA	LRI	984	586
Snoeck^343^	2021	Laos		436	28
Ramezannia^344^	2021	Iran		100	18
Raju^345^	2021	India	LRI	317	96
Mathisen^346^	2021	Nepal	Pneumonia	610	299
Mandelia^347^	2021	USA		4947	1228
Lin^348^	2021	China		2853	332
Lin^348^	2021	China		1222	202
Lim^349^	2021	South Korea		6576	1106
Li^350^	2021	China		2298	152
Li^350^	2021	China		3398	683
Leli^351^	2021	Italy		197	37
Lei^352^	2021	Macao		4880	757
Komoyo^353^	2021	Central African Republic		3903	312
Khomenko^354^	2021	Ukraine		487	64
Juliana^355^	2021	Suriname	SARI	316	107
Ihling^356^	2021	Tanzania		293	9
Ihling^356^	2021	Burkina Faso		115	2
Ihling^356^	2021	Gabon		182	4
Ihling^356^	2021	Ghana		490	31
Haddadin^357^	2021	USA		360	101
Guo^358^	2021	China		11,306	1783
El-Senousy^359^	2021	Egypt		100	2
Diesner-Treiber^360^	2021	Austria		448	0
Correia^361^	2021	Cabo Verde		129	13
Chen^362^	2021	China	LRI	5529	964
Arshad^363^	2021	Pakistan	LRI	70	21
Agca^364^	2021	Turkey	URI	248	6
Vittucci^365^	2021	Italy		6209	1415
Vittucci^365^	2021	Italy		615	5
Zhang^366^	2022	China	Pneumonia	2364	242
Zhang^366^	2022	China	Pneumonia	375	74
Yun^367^	2022	USA	Pneumonia	441	75
Xu^368^	2022	China	LRI	417	50
Xu^368^	2022	China	LRI	632	25
Xiang^369^	2022	China		1442	492
Windsor^370^	2022	USA		931	114
Tabatabai^371^	2022	Germany		946	405
Suryadevara^372^	2022	Ecuador		820	99
Sen Zeynep^373^	2022	Turkey	LRI	255	99
Sarkar^374^	2022	India	LRI	349	142
Pretell^375^	2022	Peru		79	4
Paul Shen^376^	2022	Belgium		360	65
Paul Shen^376^	2022	Belgium		93	3
Orqueda^377^	2022	Argentina		619	158
Ogunbayo^378^	2022	South Africa	SARI	84	40
Nenna^379^	2022	Italy		476	130
Nenna^379^	2022	Italy		85	4
Moleleki^380^	2022	South Africa		154	39
Jiang^381^	2022	China		3338	666
Jiang^381^	2022	China		5860	581
Meyer^382^	2022	Germany		748	169
Maglione^383^	2022	Italy		1763	733
Low^384^	2022	Malaysia		23,306	3652
Lokida^385^	2022	Indonesia	Pneumonia	188	51
Lei^386^	2022	China		4880	755
Kume^387^	2022	Japan		1757	639
Kume^388^	2022	Japan		743	275
Kume^388^	2022	Japan		422	113
Koul^389^	2022	India	SARI	412	118
Kamata^390^	2022	Myanmar	LRI	570	262
Kafntu-Kwashie^391^	2022	Ghana	LRI	188	20
Jamieson^392^	2022	USA		274	86
Hossain^393^	2022	Bangladesh		3170	555
Hanchi^394^	2022	Morocco	SARI	586	149
Hanchi^394^	2022	Morocco	SARI	316	65
Davis^395^	2022	New Zealand	SARI	3169	1258
Dananche^396^	2022	Multiple countries	Pneumonia	888	112
Dai^397^	2022	China		63,392	7105
Cui^398^	2022	China		6481	824
Cui^398^	2022	China		1508	230
Ng^399^	2022	Malaysia	Pneumonia	111	31
Chawla^400^	2022	India	LRI	50	7
Chandy^401^	2022	India		256	92
Cason^402^	2022	Italy		1227	1
Bimouhen^403^	2022	Morocco		740	282
Ahmed^404^	2022	Saudi Arabia		580	164
Shen^405^	2022	China		541	106
Letafati^406^	2022	Iran		168	0
Calaor-Morin^407^	2022	Philippines		1036	122
Agarwal^408^	2023	India	URI	180	32
Alaib^409^	2023	Saudi Arabia		521	189
Alaib^409^	2023	Saudi Arabia		205	27
Almeida^410^	2023	Portugal		626	141
Alsayed^411^	2023	Jordan	Bronchiolitis	91	42
Atti^412^	2023	Italy		35,746	1927
Atti^412^	2023	Italy		37,213	1469
DeJonge^413^	2023	USA		1418	135
Edderdouri^414^	2023	Morocco		178	36
Fourie^415^	2023	Netherland	URI	88	30
Guo^416^	2023	China		1225	267
Han^417^	2023	China	URI	252	2
Han^417^	2023	China	LRI	785	186
Kandeel^418^	2023	Egypt	ILI	497	72
Kang^419^	2023	India	LRI	166	85
Kang^419^	2023	India	LRI	189	9
Kelly^420^	2023	Tanzania		2082	544
Kislal^421^	2023	Turkey		207	0
Krumkamp^422^	2023	Ghana	LRI	327	16
Kumar^423^	2023	India		94	5
Kurskaya^424^	2023	Russia		1088	229
Kurskaya^424^	2023	Russia		2102	200
Yuan Li^425^	2023	China	Pneumonia	9837	1507
Ming Li^426^	2023	China		556	117
Lin^427^	2023	Taiwan	Pneumonia	128	29
Mai^428^	2023	China		86	0
Mai^428^	2023	China		157	17
Osborne^429^	2023	USA	LRI	295	103
Ramgopal^430^	2023	USA	Pneumonia	573	114
Samuels^431^	2023	Sierra Leone		502	98
Shi^432^	2023	China		10,396	1655
Siddik^433^	2023	Bangladesh		320	21
Steponaviciene^434^	2023	Lithuania		5127	429
Vasconcelos^435^	2023	Switzerland	Pneumonia	138	31
Virant^436^	2023	Slovenia		3107	378
Virant^436^	2023	Slovenia		3316	411
Wadilo^437^	2023	Ethiopia	LRI	210	64
Xu^438^	2023	China	SARI	262	20
Xu^438^	2023	China	SARI	711	43
Yan Yan^439^	2023	China	LRI	989	317
Yi Yan^440^	2023	China	LRI	744	106
Zarur-Torralvo^441^	2023	Colombia		1249	178
Zarur-Torralvo^441^	2023	Colombia		231	8
Zdanowicz^442^	2023	Poland	LRI	100	5
Zendehrouh^443^	2023	Iran		87	2
Zhang^444^	2023	China		2632	535
Bimouhen^445^	2023	Morocco		1882	579
Hayek^446^	2023	USA		30,283	3506
Kandeel^447^	2023	Egypt	SARI	317	153
Kubale^448^	2023	Albania		1032	438
Kubale^448^	2023	Jordan		1056	358
Kubale^448^	2023	Nicaragua		936	208
Kubale^448^	2023	Philippines		607	123
Morgan^449^	2023	South Africa		460	142
Naeem^450^	2023	Iraq		158	15
Rybak^451^	2023	France	Bronchiolitis	984	437
Salim^452^	2023	UAE		3098	530
Suh^453^	2023	South Korea	Pneumonia	517	71
Trang^454^	2023	Vietnam	SARI	1563	438
Umar^455^	2023	China		6499	405
Wadilo^456^	2023	Ethiopia		210	64
Wanlapakorn^457^	2023	Thailand	SARI	169	49
Zendehrouh^443^	2023	Iran		87	2
Alimohammadi^458^	2024	Iran		102	23
Altawalah^459^	2024	Kuwait		367	94
Aneja^460^	2024	India	SARI	840	257
Begley^461^	2024	USA		1741	234
Bhardwaj^462^	2024	India		3171	357
Buonsenso^463^	2024	Italy		523	152
Do^464^	2024	Mongolia		5705	2113
Dorji^465^	2024	Bhutan	SARI	921	231
Farzi^466^	2024	Iran		340	11
Fröhlich^467^	2024	Brazil		748	612
Hou^468^	2024	China		19,531	3215
Huang^469^	2024	China		117	52
Korsun^470^	2024	Bulgaria		2241	302
Kuang^16^	2024	China		155,165	2524
Lebreiro^471^	2024	Brazil		369	55
Leija-Martínez^472^	2024	Mexico		390	160
Li^473^	2024	China		44,704	4018
Li^474^	2024	China		6864	376
Li^475^	2024	China		4565	273
Liu^476^	2024	China		5453	804
Liu^477^	2024	China		1344	186
Lv^478^	2024	China		4804	334
Ma^479^	2024	China	Pneumonia	309	82
Meier^480^	2024	Austria		329	110
Menezes^481^	2024	Brazil		54,685	17,626
Mojarrad^482^	2024	Iran		200	34
Moyes^483^	2024	South Africa		5786	1079
Ndiaye^484^	2024	Senegal		159	11
Pan^485^	2024	China		1374	54
Pasittungkul^486^	2024	Thailand		7710	1245
Pérez-Camacho^487^	2024	Colombia	Pneumonia	61	24
Philomenadin^488^	2024	India		1684	420
Pun^489^	2024	China		24,734	2144
Ramzali^490^	2024	Iran		411	111
Reddy^491^	2024	South Africa		1358	256
Reller^492^	2024	Bangladesh		1477	299
Rojo-Alba^493^	2024	Spain		65,382	4765
Shrestha^494^	2024	Nepal	ILI	803	132
Simusika^495^	2024	Zambia	SARI	3113	504
Stacevičienė^496^	2024	Lithuania		7014	431
Sun^497^	2024	China		345	17
Tayachew^498^	2024	Ethiopia		2234	362
Tran^499^	2024	Vietnam	Pneumonia	467	114
Umran^500^	2024	India		100	7
Wei^501^	2024	China		965	57
Wu^502^	2024	China		11,056	1501
Xu^503^	2024	China		42,379	6394
Yang^504^	2024	China	Pneumonia	7533	1051
Yang^505^	2024	China		15,993	1561
Zhang^506^	2024	China		4956	342
Zhang^507^	2024	China		4219	217
Zhao^508^	2024	China		1090	181
Zhao^509^	2024	China		1788	186
Zheng^510^	2024	China		1939	184
Adu-Gyamfi^511^	2025	Ghana		303	27
Bandeira^512^	2025	Brazil		465	185
Burrell^513^	2025	Australia		32,599	3338
Cha^514^	2025	China		10,580	474
Correia^515^	2025	Cabo Verde		96	37
Han^516^	2025	China		1184	338
Jamalidoust^517^	2025	Iran	SARI	155	0
Jiang^518^	2025	China		7131	732
Jiang^519^	2025	China		8454	452
Jie^520^	2025	China		740	84
Khan^521^	2025	Bangladesh	ILI	390	42
Lai^522^	2025	China		12,993	917
Li^523^	2025	China		3966	817
Liu^524^	2025	China		8550	805
Ma^525^	2025	China		1691	234
Matache^526^	2025	Romania		803	43
Matsumura^527^	2025	Japan		212	21
Mi^528^	2025	China	ILI	28,217	1562
Moleleki^529^	2025	South Africa	SARI	198	54
Mollel^17^	2025	Tanzania		11	1
Pale^530^	2025	Mozambique	SARI	472	109
Hosseinpour Sadeghi^531^	2025	Iran		92	2
Santos^532^	2025	Brazil		1081	344
Shrestha^533^	2025	Nepal	Pneumonia	1363	282
Soares^534^	2025	Brazil	SARI	66,170	18,026
Takashita^535^	2025	Japan	ILI	2177	218
Tan^536^	2025	China	Pneumonia	110	39
Tang^537^	2025	China		15,397	2177
Tayachew^538^	2025	Ethiopia		2990	628
Wang^539^	2025	China		40,174	5677
Wang^540^	2025	China		16,571	2361
Wu^541^	2025	China		12,743	1156
Xu^542^	2025	China	Bronchiolitis	697	300
Xu^543^	2025	China	Pneumonia	7635	991
Zeng^544^	2025	China		14,352	2125
Zhang^545^	2025	China		682	77
Zhu^546^	2025	China		3790	457

**Table 2:** Subgroup analysis of the prevalence of hRSV infection among pediatric patients with respiratory infections.

	Group	Number of datasets	Total sample size	Pooled prevalence (%) (95% CI)	Heterogeneity test I^2^%, p-value	Differences between subgroups; χ2 test (*p*-value)
Overall prevalence	–	584	1,733,341	21.6 (20.5–22.6)	99.6%, *P < 0.0001*	–
Study period	Year-round	432	1,547,640	20.2 (19.1–21.4)	99.6%, *P < 0.0001*	*P < 0.0001*
	Peak season	148	184,312	25.7 (22.8–28.8)	99.4%, *P < 0.0001*	
Sample type	NP	302	630,169	24.4 (22.9–26.0)	99.4%, *P < 0.0001*	*P < 0.0001*
	Throat	35	69,754	11.8 (9.4–14.4)	98.9%, *P < 0.0001*	
	Nasal	38	23,900	24.8 (19.9–29.9)	98.6%, *P < 0.0001*	
	OP	8	20,344	14.1 (9.1–20.1)	98.9%, *P < 0.0001*	
	Tracheal	2	457	45.7 (41.1–50.3)	NA	
	Sputum	4	3806	18.8 (7.9–32.9)	98.6%, *P < 0.0001*	
	BAL	2	1199	12.3 (10.5–14.2)	NA	
Sample type (Overall)	URS	498	1,019,307	22.0 (20.9–23.1)	99.3%, *P < 0.0001*	*P < 0.0001*
	MRS	66	516,375	17.3 (14.9–19.9)	99.8%, *P < 0.0001*	
	LRS	8	5462	23.3 (14.4–33.6)	98.3%, *P < 0.0001*	
Type of disease	ILI	47	116,075	13.7 (11.5–16.1)	99.09%, *P < 0.0001*	*P < 0.0001*
	SARI	32	90,373	25.8 (22.2–29.5)	98.6%, *P < 0.0001*	
	Pneumonia	57	84,366	22.5 (20.4–24.6)	97.5%, *P < 0.0001*	
	Bronchiolitis	32	14,913	56.9 (50.6–63.2)	98.2%, *P < 0.0001*	
	Wheezing	7	1309	32.5 (19.8–46.7)	95.5%, *P < 0.0001*	
Type of disease (Overall)	URTI	56	120,047	13.2 (11.1–15.3)	98.9%, *P < 0.0001*	*P < 0.0001*
	LRTI	224	357,450	30.2 (28.3–32.1)	99.2%, *P < 0.0001*	
Sampling time	1991–2000	9	6090	31.0 (21.6–41.3)	98.3%, *P < 0.0001*	*P < 0.0001*
	2001–2010	124	80,362	28.0 (25.1–31.0)	98.8%, *P < 0.0001*	
	2011–2019	294	699,849	21.6 (20.4–22.9)	99.3%, *P < 0.0001*	
	2020–2024	117	572,979	15.1 (13.4–17.0)	99.7%, *P < 0.0001*	
Gender	Male	72	104,412	24.0 (21.6–26.5)	98.6%, *P < 0.0001*	*P = 0.55*
	Female	72	80,996	22.9 (20.5–25.3)	98.1%, *P < 0.0001*	
Economy classification	Low-income	23	16,662	21.8 (16.6–27.5)	98.5%, *P < 0.0001*	*P < 0.0001*
	Middle-income	374	1,175,838	19.6 (18.3–20.9)	99.6%, *P < 0.0001*	
	High-income	184	539,079	25.8 (23.8–27.9)	99.6%, *P < 0.0001*	
Age (month)	0–6	60	68,866	33.8 (30.3–37.4)	98.6%, *P < 0.0001*	*P < 0.0001*
	7–12	42	29,509	23.8 (19.5–28.3)	98.3%, *P < 0.0001*	
	13–24	60	30,945	17.3 (14.4–20.4)	97.3%, *P < 0.0001*	
	25–36	17	7329	10.4 (7.8–13.3)	80.7%, *P < 0.0001*	
	37–48	13	5311	4.9 (2.1–8.5)	85.9%, *P < 0.0001*	
	49–60	14	4232	1.8 (0.1–4.5)	77.7%, *P < 0.0001*	
Age (year)	0–5	369	688,375	25.2 (23.9–26.6)	99.3%, *P < 0.0001*	*P < 0.0001*
	6–18	94	107,328	4.6 (3.7–5.6)	97.4%, *P < 0.0001*	
Patient type	Outpatients	61	50,112	11.1 (9.2–13.2)	97.8%, *P < 0.0001*	*P < 0.0001*
	Inpatients	339	920,717	25.9 (24.2–27.7)	99.7%, *P < 0.0001*	

### Prevalence of hRSV infection among children with respiratory infections

The overall pooled prevalence of hRSV infection among 1,733,341 pediatric patients with respiratory infections was 21.6% (95% CI: 20.5%–22.6%; I^2^ = 99.6%, *P* < 0.0001). The prevalence of hRSV varied significantly across different respiratory conditions (*P* < 0.0001). The highest prevalence of hRSV was observed in bronchiolitis (56.9%, 95% CI: 50.6%–63.2%), followed by wheezing (32.5%, 95% CI: 19.8%–46.7%), severe acute respiratory infection (SARI) (25.8%, 95% CI: 22.2%–29.5%), and pneumonia (22.5%, 95% CI: 20.4%–24.6%).

The prevalence of hRSV was the highest in studies conducted between 1991 and 2000 (31.0%, 95% CI: 21.6%–41.3%), followed by 2001–2010 (28.0%, 95% CI: 25.1%–31.0%). The differences between time periods were statistically significant (*P* < 0.0001). Additionally, the highest number of studies conducted (n = 294) and respiratory samples collected (n = 699,849) were recorded between 2011 and 2019.

The prevalence of hRSV was slightly higher in males (24.0%, 95% CI: 21.6%–26.5%) compared to females (22.9%, 95% CI: 20.5%–25.3%), but the difference was not statistically significant (*P* = 0.55). The highest prevalence of hRSV was observed in children aged 0–6 months (33.8%, 95% CI: 30.3%–37.4%), followed by 7–12 months (23.8%, 95% CI: 19.5%–28.3%). The differences between age groups were statistically significant (*P* < 0.0001). Overall, the prevalence of hRSV was significantly higher in children aged 0–5 years (25.2%, 95% CI: 23.9%–26.6%) compared to those aged 6–18 years (4.6%, 95% CI: 3.7%–5.6%) (*P* < 0.0001).

The prevalence of hRSV was higher among inpatients (25.9%, 95% CI: 24.2%–27.7%) compared to outpatients (11.1%, 95% CI: 9.2%–13.2%), with a statistically significant difference (*P* < 0.0001). In total, 37,536 hRSV positive samples were typed, among which 20,937 (55.7%) belonged to type A and 16,599 (44.3%) belonged to type B.

### Geographic distribution of hRSV

Subgroup analysis of the prevalence of hRSV infection among pediatric patients with respiratory tract infection revealed considerable geographic variation in 97 countries, as shown in Table 3. Pooled estimates of prevalence varied greatly, ranging from 1.7% (95% CI: 0.2–6.1) in Burkina Faso to 79.8% (95% CI: 75.9–83.3) in Croatia, reflecting differing epidemiological patterns globally.

**Table 3:** Subgroup analysis of the prevalence of hRSV infection among pediatric patients with respiratory tract infections based on geographic areas.

Country	No. of Studies	Total sample size	Pooled prevalence (%) (95% CI)	Heterogeneity test I^2^%, *p*-value
Albania	1	1032	42.4 (39.4–45.5)	NA
Algeria	1	117	47.8 (38.5–57.2)	NA
Argentina	1	619	25.5 (22.1–29.1)	NA
Australia	7	37,240	18.2 (10.2–27.9)	99.2%, *P < 0.0001*
Austria	2	777	7.2 (5.5–9.2)	NA
Bangladesh	6	5916	14.9 (10.5–20.0)	95.0%, *P < 0.0001*
Belgium	3	592	12.7 (4.8–23.6)	NA
Bhutan	1	921	25.0 (22.3–28.0)	NA
Bolivia	1	274	21.9 (17.1–27.2)	NA
Brazil	23	128,602	35.6 (31.8–39.5)	99.0%, *P < 0.0001*
Bulgaria	5	4519	24.9 (16.3–34.6)	97.7%, *P < 0.0001*
Burkina Faso	1	115	1.7 (0.2–6.1)	NA
Cambodia	2	1182	19.4 (17.2–21.8)	NA
Cameroon	1	347	13.2 (9.8–17.2)	NA
Cape Verde	2	225	20.6 (15.5–26.1)	NA
Central African Republic	2	4232	7.4 (6.7–8.3)	NA
China	120	834,429	15.1 (13.4–16.9)	99.7%, *P < 0.0001*
Colombia	4	2125	13.1 (6.3–21.9)	95.2%, *P < 0.0001*
Congo	2	146	20.2 (13.9–27.2)	NA
Costa Rica	1	379	4.2 (2.4–6.7)	NA
Croatia	1	486	79.8 (75.9–83.3)	NA
Cyprus	2	815	31.5 (28.3–34.7)	NA
Ecuador	2	1226	19.8 (17.6–22.0)	NA
Egypt	7	3696	21.8 (12.4–32.9)	97.1%, *P < 0.0001*
Ethiopia	4	5644	23.4 (18.3–29.0)	93.7%, *P < 0.0001*
Finland	4	2556	17.9 (11.3–25.4)	82.1%, *P = 0.0008*
France	5	14,309	38.3 (21.9–56.2)	99.6%, *P < 0.0001*
Gabon	2	1103	10.1 (8.4–12.0)	NA
Gambia	2	1254	26.7 (24.3–29.2)	NA
Germany	7	6724	24.5 (16.2–33.8)	98.5%, *P < 0.0001*
Ghana	8	4217	14.3 (9.1–20.6)	95.4%, *P < 0.0001*
Greece	4	2041	17.7 (9.7–27.4)	95.0%, *P < 0.0001*
Haiti	1	101	25.7 (17.5–35.4)	NA
Honduras	1	345	7.5 (4.9–10.8)	NA
India	32	16,146	20.3 (16.7–24.1)	97.0%, *P < 0.0001*
Indonesia	2	990	21.5 (19.0–24.1)	NA
Iran	22	4446	14.6 (9.7–20.2)	95.8%, *P < 0.0001*
Iraq	3	577	15.6 (9.5–22.8)	NA
Italy	24	89,204	25.5 (19.2–32.4)	99.7%, *P < 0.0001*
Ivory Coast	3	1529	12.7 (3.4–26.5)	NA
Japan	13	8423	29.0 (21.4–37.2)	98.3%, *P < 0.0001*
Jordan	4	4648	41.1 (34.8–47.6)	91.4%, *P < 0.0001*
Kenya	5	12,407	14.6 (5.9–26.5)	99.5%, *P < 0.0001*
Kuwait	1	367	25.6 (21.2–30.4)	NA
Laos	3	1064	17.8 (2.4–42.7)	NA
Latvia	1	207	42.5 (35.6–49.5)	NA
Lebanon	1	24	41.6 (22.1–63.3)	NA
Lithuania	2	12,141	7.0 (6.6–7.5)	NA
Madagascar	4	1299	24.6 (9.3–44.2)	97.8%, *P < 0.0001*
Malaysia	7	24,612	27.9 (19.3–37.4)	96.6%, *P < 0.0001*
Mali	1	118	25.4 (17.8–34.2)	NA
Mexico	5	2680	18.8 (8.7–31.7)	97.8%, *P < 0.0001*
Mozambique	2	896	24.7 (21.9–27.6)	NA
Mongolia	2	5813	36.6 (35.4–37.9)	NA
Morocco	7	4442	30.5 (25.4–35.8)	91.7%, *P < 0.0001*
Myanmar	1	570	45.9 (41.8–50.1)	NA
Nepal	5	7049	19.5 (8.1–34.4)	99.4%, *P < 0.0001*
Netherlands	11	2351	38.0 (21.4–56.2)	98.7%, *P < 0.0001*
New Guinea	1	167	13.1 (8.4–19.2)	NA
New Zealand	3	4889	37.3 (31.4–43.4)	NA
Nicaragua	1	936	22.2 (19.6–25.0)	NA
Niger	2	798	25.5 (22.5–28.6)	NA
Nigeria	1	231	17.7 (13.0–23.3)	NA
Norway	2	2352	42.8 (40.8–44.8)	NA
Pakistan	6	3257	32.0 (12.3–55.7)	99.2%, *P < 0.0001*
Paraguay	1	99	14.1 (7.9–22.5)	NA
Peru	3	942	14.6 (7.2–23.9)	NA
Philippines	7	10,633	18.5 (14.1–23.4)	97.2%, *P < 0.0001*
Poland	2	1196	6.3 (5.0–7.8)	NA
Portugal	3	1589	32.0 (2.9–73.4)	NA
Qatar	3	32,084	38.1 (16.9–62.0)	NA
Romania	2	1044	8.0 (6.4–9.7)	NA
Russia	3	4750	17.3 (9.0–27.6)	NA
Saudi Arabia	9	4180	22.5 (17.6–27.9)	91.3%, *P < 0.0001*
Senegal	5	3434	14.1 (11.0–17.6)	65.4%, *P = 0.02*
Sierra Leone	1	502	19.5 (16.1–23.2)	NA
Singapore	2	4519	7.9 (7.1–8.8)	NA
Slovenia	3	6701	16.0 (11.7–20.9)	NA
South Africa	8	20,181	24.8 (20.1–29.9)	97.8%, *P < 0.0001*
South Korea	9	63,366	19.2 (12.9–26.3)	99.7%, *P < 0.0001*
Spain	10	88,651	31.9 (22.3–42.3)	99.8%, *P < 0.0001*
Sri Lanka	1	325	28.6 (23.7–33.8)	NA
Sudan	1	368	7.0 (4.6–10.1)	NA
Suriname	1	316	33.8 (28.6–39.3)	NA
Switzerland	1	138	22.4 (15.8–30.3)	NA
Taiwan	5	1350	15.5 (9.3–22.9)	90.7%, *P < 0.0001*
Tanzania	3	2386	11.1 (0.0–36.3)	NA
Thailand	15	39,890	22.5 (18.7–26.5)	98.6%, *P < 0.0001*
Tunisia	2	887	33.8 (30.7–36.9)	NA
Turkey	21	8926	21.1 (14.6–28.4)	98.2%, *P < 0.0001*
United Arab Emirates	1	3098	17.1 (15.8–18.4)	NA
United Kingdom	6	65,055	29.9 (19.0–42.2)	97.8%, *P < 0.0001*
United States	28	71,365	30.1 (22.7–38.0)	99.7%, *P < 0.0001*
Ukraine	1	487	13.1 (10.2–16.4)	NA
Vietnam	7	5364	30.1 (24.4–36.2)	95.3%, *P < 0.0001*
Yemen	2	1205	42.3 (39.5–45.1)	NA
Zambia	2	3609	17.0 (15.8–18.2)	NA

In Africa, there was a range of prevalence of 1.7% (95% CI: 0.2–6.1) in Burkina Faso to 30.5% (95% CI: 25.4–35.8) in Morocco. Noteworthy is Algeria with 47.8% (95% CI: 38.5–57.2) with one study and sample of 117. In Senegal, it was more mid-range at a prevalence of 14.1% (95% CI: 11.0–17.6) with lower heterogeneity (I^2^ = 65.4%, *P* = 0.02). This is in contrast to Kenya having a prevalence of 14.6% (95% CI: 5.9–26.5) and significant heterogeneity (I^2^ = 99.5%, *P* < 0.0001), indicating that its five studies were different.

In the Americas, prevalence ranged from 4.2% (95% CI: 2.4–6.7) in Costa Rica to 35.6% (95% CI: 31.8–39.5) in Brazil. The United States had a combined prevalence of 30.1% (95% CI: 22.7–38.0) from 28 studies with high heterogeneity (I^2^ = 99.7%, *P* < 0.0001). Brazil with 23 studies and 128,602 sample size also reported high heterogeneity (I^2^ = 99.0%, *P* < 0.0001), which showed diverse infection rates within the country.

Among Asian countries included in the analysis, pooled prevalence rates varied widely, reflecting diverse epidemiological patterns. The highest pooled prevalence was observed in Myanmar at 45.9% (95% CI: 41.8–50.1), based on a single study with 570 participants, followed closely by Yemen at 42.3% (95% CI: 39.5–45.1) from two studies totaling 1205 individuals. In contrast, the lowest pooled prevalence was recorded in Singapore at 7.9% (95% CI: 7.1–8.8) across two studies with 4519 participants. Other notable high-prevalence countries included Jordan (41.1%, 95% CI: 34.8–47.6) and Mongolia (36.6%, 95% CI: 35.4–37.9), whereas relatively lower rates were seen in South Korea (19.2%, 95% CI: 12.9–26.3) and Taiwan (15.5%, 95% CI: 9.3–22.9), despite large sample sizes in some cases. High heterogeneity (I^2^ > 90%, *P* < 0.0001) was common in countries with multiple studies, such as China, India, and Japan, underscoring significant variability in prevalence estimates across studies within the same nation.

Among European countries included in the analysis, pooled prevalence rates varied widely, reflecting diverse study populations and methodologies. Croatia reported the highest pooled prevalence at 79.8% (95% CI: 75.9–83.3) based on a single study of 486 participants, while Poland recorded the lowest at 6.3% (95% CI: 5.0–7.8) across two studies totaling 1196 individuals. High heterogeneity (I^2^ > 95%, *P* < 0.0001) was observed in countries with multiple studies, such as France, Germany, Italy, and Spain, indicating substantial variation across individual studies within these nations.

**Fig. 2:**
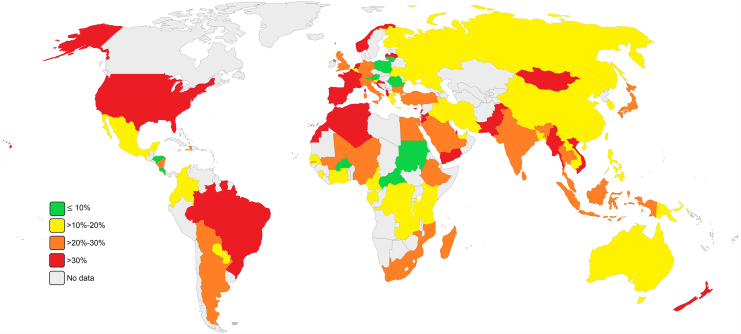
The global map presents the geographical variations in the prevalence of hRSV infection among pediatric patients with respiratory infections in a period of 33 years.

Within Oceania, Australia's prevalence was reported at 18.2% (95% CI: 10.2–27.9) based on seven studies with high heterogeneity (I^2^ = 99.2%, *P* < 0.0001) and New Zealand had a higher prevalence of 37.3% (95% CI: 31.4–43.4) based on three studies. Fig. 2 depicts the global distribution of hRSV infection among pediatric patients with respiratory infections.

### The association between hRSV infection and respiratory infections among pediatric patients

**Fig. 3:**
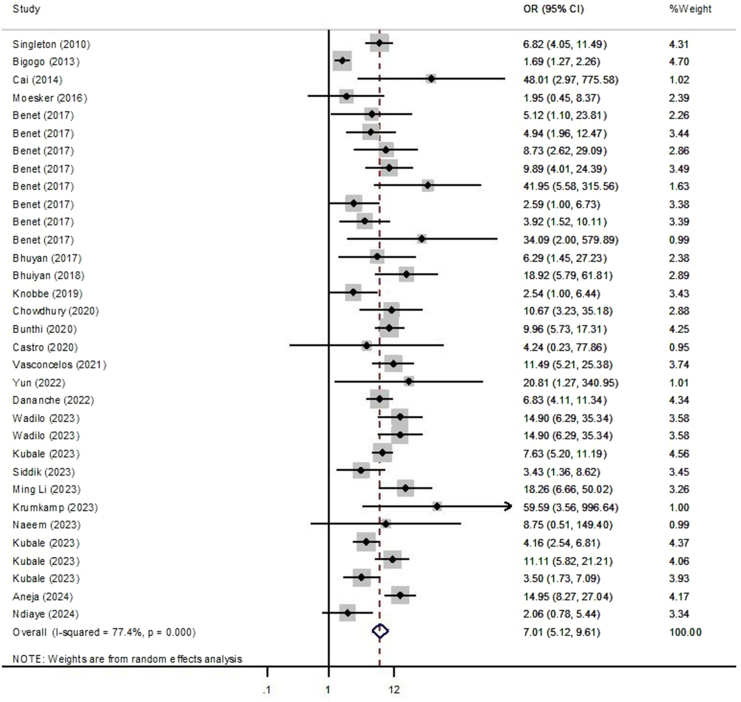
Forest plot of the association between hRSV infection and respiratory infection risk in pediatric patients according to the random effect model using case case–control studies.

The second meta-analysis, focusing on case–control studies, assessed the association between hRSV infection and the risk of respiratory infections among pediatric patients. A total of 33 datasets were included, comprising 18,345 pediatric patients with respiratory infections and 7975 controls. Using a random-effects model, the overall pooled odds ratio (OR) was calculated as 7.0 (95% CI: 5.1–9.6; I^2^ = 77.4%, *P* < 0.0001), indicating a strong association between hRSV infection and increased risk of respiratory infections (Fig. 3).

Subgroup analyses were conducted to explore this association further based on type of disease, economy classification, sample type, and sampling time. When stratified by type of disease, the pooled OR for SARI based on one dataset was 14.9 (95% CI: 8.2–27.0). For pneumonia, 13 datasets yielded a pooled OR of 7.6 (95% CI: 5.4–10.6; I^2^ = 26.0%, *P* = 0.1).

When analyzed by sampling time, studies conducted between 2001 and 2010 (four datasets) had a pooled OR of 3.9 (95% CI: 1.3–11.9) with significant heterogeneity (I^2^ = 88.3%, *P* < 0.0001). For the period of 2011–2019, 24 datasets yielded a pooled OR of 7.1 (95% CI: 5.5–9.1) with moderate heterogeneity (I^2^ = 47.4%, *P* = 0.006). The most recent period, 2020 to 2024, based on five datasets, showed the highest pooled OR of 9.5 (95% CI: 3.9–23.1; I^2^ = 69.7%, *P* = 0.01), suggesting a potentially stronger association in recent years, though limited by fewer studies. The subgroup analysis of the association between hRSV infection and the risk of respiratory infections among pediatric patients is presented in Table 4.

**Table 4:** Subgroup analysis of association between hRSV infection and respiratory infections risk among pediatric patients.

Characteristics	Categories	No. of datasets	Pooled ORs (95% CI)	Heterogeneity: I^2^%, *P* value
Overall	–	33	7.0 (5.1–9.6)	77.4%, *P < 0.0001*
Economy classification	Low	4	9.5 (5.1–17.7)	34.7%, *P = 0.2*
	Middle	23	6.5 (4.3–9.7)	80.9%, *P < 0.0001*
	High	4	7.5 (3.1–18.1)	51.9%, *P = 0.3*
Sample type	NP	17	6.7 (4.8–9.3)	39.5%, *P = 0.04*
	Nasal	1	6.2 (1.4–27.2)	NA
	OP	2	6.7 (0.9–46.5)	87.4%, *P = 0.005*
	Throat	1	59.6 (3.5–996.6)	NA
Sample type (Overall)	URS	32	7.2 (5.2–9.9)	77.8%, *P < 0.0001*
	MRS	1	1.9 (0.4–8.3)	NA
Type of disease	Pneumonia	13	7.6 (5.4–10.6)	26.0%, *P = 0.1*
	SARI	1	14.9 (8.2–27.0)	NA
Sampling time	2001–2010	4	3.9 (1.3–11.9)	88.3%, *P < 0.0001*
	2011–2019	24	7.1 (5.5–9.1)	47.4%, *P = 0.006*
	2020–2024	5	9.5 (3.9–23.1)	69.7%, *P = 0.01*

**Fig. 4:**
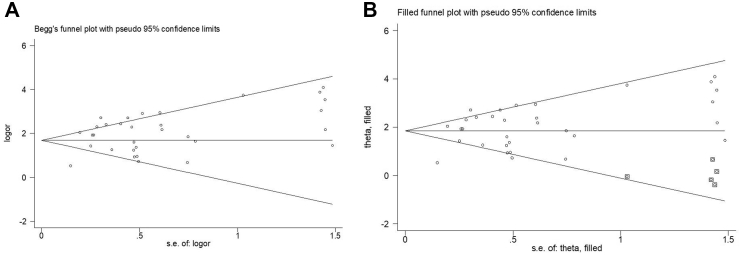
Funnel plots before (A) and after (B) applying the trim-and-fill method. The open dots indicate the observed studies, and the closed dots indicate the missing studies imputed by the trim-and-fill method.

We assessed publication bias with visual inspection of the funnel plot and statistical tests. The results showed evidence of publication bias for the association between hRSV infection and respiratory infection risk (*P* = 0.74, for Begg's adjusted rank correlation test and *P* = 0.014 for Egger's regression asymmetry test). Also, because of the potentially missing studies, the funnel plot looks fairly asymmetrical and strongly indicates publication bias. To identify and correct the publication bias, we used the trim-and-fill method, and 5 missing studies were identified (Fig. 4). After adjusting for missing studies with the ‘trim and fill’ method, the overall OR is estimated as 6.33 with a 95% confidence interval (CI) [4.65, 8.61]. Therefore, larger effects in the positive direction were likely favored in the publication process, and studies with smaller effects might be suppressed in the negative direction.

### Sensitivity analysis

In a sensitivity analysis by successively removing a particular study at a time to assess the influence of every single study on pooled results, a significant positive association [range of summary ORs 6.76–7.34] between hRSV infection and respiratory infections was observed consistently and did not alter the pooled results, which indicated that the meta-analysis model is robust.

## Discussion

This systematic review and meta-analysis provide a comprehensive synthesis of the global prevalence of hRSV among pediatric patients with RTIs, drawing from 539 studies across 97 countries over a 33-year period (1992–2024). The findings reveal a significant global burden of hRSV, with pooled prevalence estimates varying widely from 1.7% in Burkina Faso to 79.8% in Croatia, highlighting substantial geographic heterogeneity. This variability aligns with previous research indicating that hRSV epidemiology is influenced by climatic, demographic, and healthcare-related factors.^3^^,^^5^ The overall association between hRSV infection and increased risk of respiratory infections, with a pooled odds ratio (OR) of 7.0 (95% CI: 5.1–9.6), further underscores its role as a major contributor to pediatric respiratory morbidity worldwide.

The wide range of hRSV prevalence across countries underscores the complex interplay of environmental, demographic, and healthcare-related factors shaping its epidemiology. High prevalence rates in settings such as Croatia (79.8%), Algeria (47.8%), and Myanmar (45.9%) contrast sharply with low rates in Burkina Faso (1.7%), Costa Rica (4.2%), and Poland (6.3%). This variability aligns with established patterns of hRSV seasonality, where temperate regions experience peaks during colder months, and tropical areas see surges during rainy seasons.^5^ For instance, Myanmar's high prevalence may reflect increased transmission during monsoon periods, facilitated by indoor crowding and humidity, as noted in other tropical settings.^4^

Countries with multiple studies, such as China (15.1%, 120 studies), India (20.3%, 32 studies), and the United States (30.1%, 28 studies), exhibited extreme heterogeneity (I^2^ > 97%), suggesting within-country variations driven by factors like urban-rural disparities, climate zones, or differences in diagnostic practices. For example, China's vast geographic and socioeconomic diversity likely contributes to its high heterogeneity, a pattern echoed in other large nations.^8^ These findings emphasize the need for region-specific data to inform targeted interventions, as blanket assumptions about hRSV prevalence may overlook critical local dynamics.

The prevalence of hRSV varied significantly across respiratory conditions (*P* < 0.0001), with bronchiolitis exhibiting the highest rate (56.9%, 95% CI: 50.6%–63.2%), followed by wheezing (32.5%, 95% CI: 19.8%–46.7%) and SARI (25.8%, 95% CI: 22.2%–29.5%). This pattern corroborates hRSV's well-established role as the primary cause of bronchiolitis in infants, often leading to hospitalization.^2^ The elevated prevalence in wheezing and SARI further highlights its tropism for the lower respiratory tract, consistent with clinical observations of severe outcomes in young children.^4^ In contrast, lower rates in conditions like influenza-like illness (ILI; 13.7%, 95% CI: 11.5%–16.1%) suggest that hRSV is less dominant in milder or upper airway presentations, where other viruses like rhinovirus or influenza may predominate.^1^ These differences underscore the need for disease-specific approaches in hRSV management, particularly targeting severe manifestations like bronchiolitis.

The temporal analysis revealed a declining trend in hRSV prevalence, from 31.0% (1991–2000) to 15.1% (2020–2024), with statistically significant differences (*P* < 0.0001). This decline may reflect several factors, including improved infection control measures, shifts in diagnostic practices, or changes in hRSV circulation patterns. The highest prevalence in the 1991–2000 period could be linked to less stringent public health interventions or limited awareness of hRSV's burden at the time, while the 2011–2019 period, with the most studies (n = 294) and samples (n = 699,849), likely benefits from enhanced surveillance and molecular diagnostics.^5^ The sharp drop in 2020–2024 aligns with the COVID-19 pandemic's impact, where non-pharmaceutical interventions (e.g., masking, social distancing) disrupted respiratory virus transmission, followed by altered resurgence patterns post-restrictions.^6^ This temporal shift warrants further investigation to distinguish between methodological artifacts and true epidemiological changes.

In the case of age-related differences, with the highest prevalence in children aged 0–6 months (33.8%, 95% CI: 30.3%–37.4%), decreasing progressively to 1.8% (95% CI: 0.1%–4.5%) by 49–60 months (*P* < 0.0001). The overall prevalence in children aged 0–5 years (25.2%, 95% CI: 23.9%–26.6%) far exceeded that in those aged 6–18 years (4.6%, 95% CI: 3.7%–5.6%), reflecting hRSV's disproportionate impact on infants and preschoolers. This age gradient is consistent with the virus's peak severity in early life, driven by immature immune responses and smaller airway diameters, which exacerbate disease progression.^4^

The prevalence was significantly higher among inpatients (25.9%, 95% CI: 24.2%–27.7%) than outpatients (11.1%, 95% CI: 9.2%–13.2%; *P* < 0.0001), highlighting hRSV's association with severe disease requiring hospitalization. This disparity underscores its role as a major driver of healthcare utilization, particularly in young children with LRIs or bronchiolitis.^2^ Among typed samples, hRSV-A predominated (55.7%) over hRSV-B (44.3%), consistent with global patterns where subtype A often circulates more frequently, though subtype B can dominate in certain seasons or regions.^66^ This distribution has implications for vaccine development, as antigenic differences between subtypes may influence efficacy.^9^

Separate pooled prevalence estimates were calculated for peak-season and year-round studies using random-effects models. As expected, pooled prevalence was higher among peak-season studies compared to year-round studies. This reflects concentration of RSV detection during epidemic periods rather than a true difference in underlying annual burden. Therefore, peak-season prevalence should not be interpreted as annual prevalence.

The 21.6% pooled prevalence and its variation across subgroups emphasize hRSV's global significance, particularly in early childhood. The high burden in infants and inpatients supports the prioritization of preventive strategies, such as the monoclonal antibody nirsevimab, which has demonstrated efficacy in reducing severe RSV outcomes.^9^ Vaccine candidates in late-stage trials further heighten the need for accurate prevalence data to guide deployment, especially in high-risk groups like those under 12 months.^8^ The declining prevalence in recent years suggests that public health measures can mitigate transmission, offering lessons for future respiratory virus control. However, the higher burden in low-resource settings, inferred from inpatient rates, calls for enhanced diagnostic and surveillance capacity in LMICs to address underreporting.^4^

The findings of this meta-analysis underscore a robust association between hRSV infection and an increased risk of respiratory infections among pediatric patients, with an overall pooled odds ratio (OR) of 7.0 (95% CI: 5.1–9.6). This result aligns with previous evidence demonstrating hRSV as a major cause of ARI, pneumonia, and LRI in children globally. The consistency of this association across various subgroup analyses, which were stratified by economy classification, sample type, type of disease, and sampling time. This further reinforces the critical role of hRSV in pediatric respiratory morbidity.

Subgroup analysis by disease type revealed varying degrees of association, with SARI exhibiting the highest pooled OR of 14.9 (95% CI: 8.2–27.0), followed by pneumonia (OR: 7.6, 95% CI: 5.4–10.6). These findings are consistent with recent studies, such as Li et al.,^5^ which reported hRSV as a predominant etiologic agent in severe LRI cases among children under five years of age, often necessitating hospitalization. The higher OR for LRI may reflect the virus's tropism for the lower respiratory tract, leading to more severe clinical outcomes such as bronchiolitis and pneumonia, which are well-documented complications of hRSV infection.^2^

This study has several important limitations that should be acknowledged when interpreting the findings. First, despite extensive subgroup analyses, substantial heterogeneity was observed across studies (overall I^2^ > 99% for prevalence estimates), likely arising from differences in case definitions, testing indications, healthcare settings (inpatient vs. outpatient), seasonal timing, specimen types, and PCR assay performance. Although we restricted inclusion to PCR-confirmed cases to improve comparability, residual heterogeneity persists and may affect the precision of pooled estimates.

Second, although PCR is currently the gold-standard diagnostic method for hRSV, variability in assay sensitivity (primer targets, amplification platforms, and viral load thresholds) and differences in specimen collection (nasopharyngeal vs. nasal vs. throat swabs) across three decades of studies may have introduced modest detection bias. While restriction to molecular methods markedly reduced misclassification compared with older antigen-based tests, very early studies (pre-2010) using less sensitive first-generation PCR assays might slightly underestimate true prevalence, whereas recent multiplex panels could marginally overestimate it by detecting low-level or prolonged shedding.

Third, a large proportion of included studies were conducted in hospitalized children or those with severe disease, which likely overestimates hRSV prevalence and attributable risk in the broader community and outpatient settings. Conversely, in many low- and middle-income countries, especially in sub-Saharan Africa and parts of South Asia, access to PCR testing remains limited, leading to under-representation of these regions and potentially underestimating the true global burden in settings where hRSV mortality is highest.

Fourth, restriction to English-language publications and exclusion of gray literature and conference abstracts may have introduced language and publication bias, as suggested by funnel-plot asymmetry and statistical tests. Although trim-and-fill adjustment was applied, small studies with low or null prevalence may still be under-represented.

Fifth, the apparent temporal increase in the strength of association (pooled OR rising from 3.9 in 2001–2010 to 9.5 in 2020–2024) cannot be fully disentangled from improvements in diagnostic sensitivity over time vs. genuine epidemiological or virological changes. Similarly, the marked decline in hRSV detection during 2020–2024 and subsequent rebound reflect the profound impact of COVID-19 non-pharmaceutical interventions rather than long-term secular trends.

Additionally, due to inconsistent or incomplete reporting of the exact month/season of sample collection in many included studies, we were unable to perform a reliable subgroup analysis by season (e.g., winter vs. rainy season in tropical climates). This is an important limitation, as hRSV circulation is highly seasonal and prevalence can vary several-fold between peak and off-season periods, potentially masking true regional and climatic differences in our global pooled estimates.

In conclusion, this systematic review and meta-analysis, the first of its kind to comprehensively assess the global prevalence of hRSV among children with RTIs, reveals a pooled prevalence of 22.7% across 846,678 pediatric patients. The findings affirm hRSV as a major contributor to pediatric respiratory morbidity, with a pronounced burden in infants aged 0–6 months (35.3%) and inpatients (27.9%), particularly those with bronchiolitis (57.8%). The significant association with severe respiratory diseases, evidenced by a pooled odds ratio (OR) of 7.0, and up to 14.9 for SARI, underscores hRSV's role in driving hospitalization and healthcare utilization worldwide. The study highlights substantial regional, temporal, and demographic variations, with prevalence declining from 31.0% (1991–2000) to 15.1% (2020–2024), potentially reflecting the impact of improved diagnostics, public health measures, and the COVID-19 pandemic's disruption of viral transmission. The predominance of hRSV-A (55.7%) over hRSV-B (44.3%) informs vaccine and therapeutic design, while the higher burden in low- and middle-income countries (LMICs) signals a need for enhanced surveillance and resource allocation. These insights emphasize the urgency of advancing targeted prevention strategies, such as vaccines and monoclonal antibodies, particularly for high-risk groups like infants and hospitalized children.

## Contributors

A.T designed and administrated the study. H.S and S.G performed all statistical analyses. P.K, M.H.R, Z.S, M.V, and S.G performed search strategy and data extraction and A.T and S.G verified the data. P.K and AT wrote the initial draft. M.H.R and H.S constructed all maps and graphs. A.M and A.T performed intellectual interpretation. All authors read and approved the final draft.

## Data sharing statement

All data included in this study are available upon request from the corresponding author.

## Editor note

The Lancet Group takes a neutral position with respect to territorial claims in published maps and institutional affiliations.

## Declaration of interests

The authors have no competing interests.

## References

[1] Walker C.L.F., Rudan I., Liu L. (2013). Global burden of childhood pneumonia and diarrhoea. Lancet.

[2] Shi T., McAllister D.A., O'Brien K.L. (2017). Global, regional, and national disease burden estimates of acute lower respiratory infections due to respiratory syncytial virus in young children in 2015: a systematic review and modelling study. Lancet.

[3] Nair H., Nokes D.J., Gessner B.D. (2010). Global burden of acute lower respiratory infections due to respiratory syncytial virus in young children: a systematic review and meta-analysis. Lancet.

[4] Scheltema N.M., Gentile A., Lucion F. (2017). Global respiratory syncytial virus-associated mortality in young children (RSV GOLD): a retrospective case series. Lancet Glob Health.

[5] Li Y., Wang X., Blau D.M. (2022). Global, regional, and national disease burden estimates of acute lower respiratory infections due to respiratory syncytial virus in children younger than 5 years in 2019: a systematic analysis. Lancet.

[6] Foley D.A., Yeoh D.K., Minney-Smith C.A. (2021). The interseasonal resurgence of respiratory syncytial virus in Australian children following the reduction of coronavirus disease 2019–related public health measures. Clin Infect Dis.

[7] Hamid S., Winn A., Parikh R. (2023). Seasonality of respiratory syncytial virus—United States, 2017–2023. MMWR Morb Mortal Wkly Rep.

[8] Shi T., Denouel A., Tietjen A.K. (2020). Global disease burden estimates of respiratory syncytial virus–associated acute respiratory infection in older adults in 2015: a systematic review and meta-analysis. J Infect Dis.

[9] Hammitt L.L., Dagan R., Yuan Y. (2022). Nirsevimab for prevention of RSV in healthy late-preterm and term infants. N Engl J Med.

[10] Moher D., Liberati A., Tetzlaff J., Altman D.G., PRISMA Group (2009). Preferred reporting items for systematic reviews and meta-analyses: the PRISMA statement. Ann Intern Med.

[11] Eslamipour F., Afshari Z., Najimi A. (2018). Prevalence of orthodontic treatment need in permanent dentition of Iranian population: a systematic review and meta-analysis of observational studies. Dent Res J.

[12] Moosazadeh M., Nekoei-moghadam M., Emrani Z., Amiresmaili M. (2014). Prevalence of unwanted pregnancy in Iran: a systematic review and meta-analysis. Int J Health Plann Manage.

[13] Harris R.J., Deeks J.J., Altman D.G., Bradburn M.J., Harbord R.M., Sterne J.A. (2008). Metan: fixed-and random-effects meta-analysis. STATA J.

[14] DerSimonian R., Laird N. (1986). Meta-analysis in clinical trials. Control Clin Trials.

[15] Higgins J.P., Thompson S.G. (2002). Quantifying heterogeneity in a meta-analysis. Stat Med.

[16] Kuang L., Xu T., Wang C. (2024). Changes in the epidemiological patterns of respiratory syncytial virus and human metapneumovirus infection among pediatric patients and their correlation with severe cases: a long-term retrospective study. Front Cell Infect Microbiol.

[17] Mollel J.S., Mziray S.R., Ndaro A. (2025). Profiles of viral pathogens from individuals with acute respiratory tract infections in northern Tanzania. IJID Reg.

[18] Cubie H.A., Inglis J., Leslie E.E., Edmunds A., Totapally B. (1992). Detection of respiratory syncytial virus in acute bronchiolitis in infants. J Med Virol.

[19] Freymuth F., Vabret A., Galateau-Salle F. (1997). Detection of respiratory syncytial virus, parainfluenzavirus 3, adenovirus and rhinovirus sequences in respiratory tract of infants by polymerase chain reaction and hybridization. Clin Diagn Virol.

[20] Gröndahl B., Puppe W., Hoppe A., Kühne I., Weigl J.A., Schmitt H.-J. (1999). Rapid identification of nine microorganisms causing acute respiratory tract infections by single-tube multiplex reverse transcription-PCR: feasibility study. J Clin Microbiol.

[21] Weigl J.A., Puppe W., Gröndahl B., Schmitt H.J. (2000). Epidemiological investigation of nine respiratory pathogens in hospitalized children in Germany using multiplex reverse-transcriptase polymerase chain reaction. Eur J Clin Microbiol Infect Dis.

[22] Zambon M.C., Stockton J.D., Clewley J.P., Fleming D.M. (2001). Contribution of influenza and respiratory syncytial virus to community cases of influenza-like illness: an observational study. Lancet.

[23] Weigl J.A., Puppe W., Schmitt H.J. (2002). Seasonality of respiratory syncytial virus-positive hospitalizations in children in Kiel, Germany, over a 7-year period. Infection.

[24] Cuevas L.E., Nasser A.M.B., Dove W., Gurgel R.Q., Greensill J., Hart C.A. (2003). Human metapneumovirus and respiratory syncytial virus, Brazil. Emerg Infect Dis.

[25] Jartti T., Lehtinen P., Vuorinen T. (2004). Respiratory picornaviruses and respiratory syncytial virus as causative agents of acute expiratory wheezing in children. Emerg Infect Dis.

[26] Jennings L.C., Anderson T.P., Werno A.M., Beynon K.A., Murdoch D.R. (2004). Viral etiology of acute respiratory tract infections in children presenting to hospital: role of polymerase chain reaction and demonstration of multiple infections. Pediatr Infect Dis J.

[27] Scott P.D., Ochola R., Ngama M. (2004). Molecular epidemiology of respiratory syncytial virus in Kilifi district, Kenya. J Med Virol.

[28] Serafino R.L., Gurgel R.Q., Dove W., Hart C.A., Cuevas L.E. (2004). Respiratory syncytial virus and metapneumovirus in children over two seasons with a high incidence of respiratory infections in Brazil. Ann Trop Paediatr.

[29] Al-Sonboli N., Hart C.A., Al-Aeryani A. (2005). Respiratory syncytial virus and human metapneumovirus in children with acute respiratory infections in Yemen. Pediatr Infect Dis J.

[30] Kotaniemi-Syrjanen A., Laatikainen A., Waris M., Reijonen T.M., Vainionpaa R., Korppi M. (2005). Respiratory syncytial virus infection in children hospitalized for wheezing: Virus-specific studies from infancy to preschool years. Acta Paediatr.

[31] Mentel R., Ilgert U., Wegner U., Zimmerman K., Bruns R., Gürtler L. (2005). Molecular and clinical characteristics of respiratory syncytial virus infections in hospitalized children. Med Microbiol Immunol.

[32] Sato M., Saito R., Sakai T. (2005). Molecular epidemiology of respiratory syncytial virus infections among children with acute respiratory symptoms in a community over three seasons. J Clin Microbiol.

[33] Versteegh F.G., Weverling G.J., Peeters M.F. (2005). Community-acquired pathogens associated with prolonged coughing in children: a prospective cohort study. Clin Microbiol Infect.

[34] Al-Sonboli N., Hart C.A., Al-Aghbari N., Al-Ansi A., Ashoor O., Cuevas L.E. (2006). Human metapneumovirus and respiratory syncytial virus disease in children, Yemen. Emerg Infect Dis.

[35] Choi E.H., Lee H.J., Kim S.J. (2006). The association of newly identified respiratory viruses with lower respiratory tract infections in Korean children, 2000-2005. Clin Infect Dis.

[36] Crowcroft N.S., Zambon M., Harrison T.G., Mok Q., Heath P., Miller E. (2008). Respiratory syncytial virus infection in infants admitted to paediatric intensive care units in London, and in their families. Eur J Pediatr.

[37] Naghipour M., Cuevas L.E., Bakhshinejad T. (2007). Contribution of viruses, Chlamydia spp. and Mycoplasma pneumoniae to acute respiratory infections in Iranian children. J Trop Pediatr.

[38] Pierangeli A., Gentile M., Di Marco P. (2007). Detection and typing by molecular techniques of respiratory viruses in children hospitalized for acute respiratory infection in Rome, Italy. J Med Virol.

[39] Teeratakulpisarn J., Ekalaksananan T., Pientong C., Limwattananon C. (2007). Human metapneumovirus and respiratory syncytial virus detection in young children with acute bronchiolitis. Asian Pac J Allergy Immunol.

[40] Thomazelli L.M., Vieira S., Leal A.L. (2007). Surveillance of eight respiratory viruses in clinical samples of pediatric patients in southeast Brazil. J Pediatr.

[41] Alper C.M., Doyle W.J., Winther B., Hendley J.O. (2008). Upper respiratory virus detection without parent-reported illness in children is virus-specific. J Clin Virol.

[42] Bonzel L., Tenenbaum T., Schroten H., Schildgen O., Schweitzer-Krantz S., Adams O. (2008). Frequent detection of viral coinfection in children hospitalized with acute respiratory tract infection using a real-time polymerase chain reaction. Pediatr Infect Dis J.

[43] Bosis S., Esposito S., Niesters H.G. (2008). Role of respiratory pathogens in infants hospitalized for a first episode of wheezing and their impact on recurrences. Clin Microbiol Infect.

[44] Calvo C., Garcia-Garcia M.L., Blanco C. (2008). Multiple simultaneous viral infections in infants with acute respiratory tract infections in Spain. J Clin Virol.

[45] Kaplan N.M., Dove W., Abd-Eldayem S.A., Abu-Zeid A.F., Shamoon H.E., Hart C.A. (2008). Molecular epidemiology and disease severity of respiratory syncytial virus in relation to other potential pathogens in children hospitalized with acute respiratory infection in Jordan. J Med Virol.

[46] Rihkanen H., Beng E.R., Nieminen T. (2008). Respiratory viruses in laryngeal croup of young children. J Pediatr.

[47] Agrawal A.S., Sarkar M., Ghosh S. (2009). Prevalence of respiratory syncytial virus group B genotype BA-IV strains among children with acute respiratory tract infection in Kolkata, Eastern India. J Clin Virol.

[48] Al-Majhdi F.N., Al-Jaralla A., Elaeed M., Latif A., Gissmann L., M Amer H. (2009). Prevalence of respiratory syncytial virus infection in Riyadh during the winter season 2007-2008 and different risk factors impact. Int J Virol.

[49] Bharaj P., Sullender W.M., Kabra S.K. (2009). Respiratory viral infections detected by multiplex PCR among pediatric patients with lower respiratory tract infections seen at an urban hospital in Delhi from 2005 to 2007. Virol J.

[50] Chun J.K., Lee J.H., Kim H.S. (2009). Establishing a surveillance network for severe lower respiratory tract infections in Korean infants and young children. Eur J Clin Microbiol Infect Dis.

[51] Fabbiani M., Terrosi C., Martorelli B. (2009). Epidemiological and clinical study of viral respiratory tract infections in children from Italy. J Med Virol.

[52] Hall C.B., Weinberg G.A., Iwane M.K. (2009). The burden of respiratory syncytial virus infection in young children. N Engl J Med.

[53] Midulla F., Scagnolari C., Bonci E. (2010). Respiratory syncytial virus, human bocavirus and rhinovirus bronchiolitis in infants. Arch Dis Child.

[54] Pavlova S., Hadzhiolova T., Abadjieva P., Kotseva R. (2009). Application of RT-PCR for diagnosis of respiratory syncytial virus and human metapneumovirus infections in Bulgaria, 2006-7 and 2007-8. Euro Surveill.

[55] Sung R.Y., Chan P.K., Tsen T. (2009). Identification of viral and atypical bacterial pathogens in children hospitalized with acute respiratory infections in Hong Kong by multiplex PCR assays. J Med Virol.

[56] Zaraket H., Dbaibo G., Salam O., Saito R., Suzuki H. (2009). Influenza virus infections in Lebanese children in the 2007-2008 season. Jpn J Infect Dis.

[57] Calvo C., Pozo F., Garcia-Garcia M.L. (2010). Detection of new respiratory viruses in hospitalized infants with bronchiolitis: a three-year prospective study. Acta Paediatr.

[58] Antunes H., Rodrigues H., Silva N. (2010). Etiology of bronchiolitis in a hospitalized pediatric population: prospective multicenter study. J Clin Virol.

[59] Faghihloo E., Rezaie F., Salimi V. (2010). Molecular epidemiology of human respiratory syncytial virus in Iranian children less than 5 years in 2007: a study on 72 cases. Tehran Uni Med J.

[60] Garcia-Garcia M.L., Calvo C., Falcon A. (2010). Role of emerging respiratory viruses in children with severe acute wheezing. Pediatr Pulmonol.

[61] Malekshahi S.S., Azad T.M., Yavarian J., Shahmahmoodi S., Naseri M., Rezaei F. (2010). Molecular detection of respiratory viruses in clinical specimens from children with acute respiratory disease in Iran. Pediatr Infect Dis J.

[62] Nascimento M.S., Souza A.V.D., Ferreira A.V.D.S., Rodrigues J.C., Abramovici S., Silva Filho L.V.F.D. (2010). High rate of viral identification and coinfections in infants with acute bronchiolitis. Clinics.

[63] Singeton R.J., Bulkow L.R., Miernyk K. (2010). Viral respiratory infections in hospitalized and community control children in Alaska. J Med Virol.

[64] Wang W., Cavailler P., Ren P. (2010). Molecular monitoring of causative viruses in child acute respiratory infection in endemo-epidemic situations in Shanghai. J Clin Virol.

[65] Zhang R.F., Jin Y., Xie Z.P. (2010). Human respiratory syncytial virus in children with acute respiratory tract infections in China. J Clin Microbiol.

[66] Zhang Z.Y., Du L.N., Chen X. (2010). Genetic variability of respiratory syncytial viruses (RSV) prevalent in Southwestern China from 2006 to 2009: emergence of subgroup B and A RSV as dominant strains. J Clin Microbiol.

[67] Pientong C., Ekalaksananan T., Teeratakulpisarn J., Tanuwattanachai S., Kongyingyoes B., Limwattananon C. (2011). Atypical bacterial pathogen infection in children with acute bronchiolitis in northeast Thailand. J Microbiol Immunol Infect.

[68] Bezerra P.G., Britto M.C., Correia J.B. (2011). Viral and atypical bacterial detection in acute respiratory infection in children under five years. PLoS One.

[69] Do A.H.L., van Doorn H.R., Nghiem M.N. (2011). Viral etiologies of acute respiratory infections among hospitalized Vietnamese children in Ho Chi Minh City, 2004–2008. PLoS One.

[70] Frobert E., Escuret V., Javouhey E. (2011). Respiratory viruses in children admitted to hospital intensive care units: evaluating the CLART (R) pneumovir DNA array. J Med Virol.

[71] Fujitsuka A., Tsukagoshi H., Arakawa M. (2011). A molecular epidemiological study of respiratory viruses detected in Japanese children with acute wheezing illness. BMC Infect Dis.

[72] Gardinassi L.G., Simas P.V.M., Salomão J.B. (2012). Seasonality of viral respiratory infections in southeast of Brazil: the influence of temperature and air humidity. Braz J Microbiol.

[73] Jin Y., Zhang R.F., Xie Z.P. (2012). Newly identified respiratory viruses associated with acute lower respiratory tract infections in children in Lanzou, China, from 2006 to 2009. Clin Microbiol Infect.

[74] Kristoffersen A.W., Nordbø S.A., Rognlien A.G., Christensen A., Døllner H. (2011). Coronavirus causes lower respiratory tract infections less frequently than RSV in hospitalized Norwegian children. Pediatr Infect Dis J.

[75] Mathisen M., Basnet S., Sharma A. (2011). RNA viruses in young Nepalese children hospitalized with severe pneumonia. Pediatr Infect Dis J.

[76] Pogka V., Kossivakis A., Kalliaropoulos A. (2011). Respiratory viruses involved in influenza-like illness in a Greek pediatric population during the winter period of the years 2005-2008. J Med Virol.

[77] Razanajatovo N.H., Richard V., Hoffmann J. (2011). Viral etiology of influenza-like illnesses in Antananarivo, Madagascar, July 2008 to June 2009. PLoS One.

[78] Salomao J.B., Gardinassi L.G.A., Simas P.V.M. (2011). Human respiratory syncytial virus in children hospitalized for acute lower respiratory infection. J Pediatr.

[79] Sezer G.M., Unsur E.K., Kayaoglu S., Akcay F. (2011). The respiratory syncytial virus may not always be responsible for Bronchiolitis in children. Afr J Microbiol Res.

[80] Simoes E.A.F., Mutyara K., Soh S., Agustian D., Hibberd M.L., Kartasasmita C.B. (2011). The epidemiology of respiratory syncytial virus lower respiratory tract infections in children less than 5 years of age in Indonesia. Pediatr Infect Dis J.

[81] Suntarattiwong P., Sojisirikul K., Sitaposa P. (2011). Clinical and epidemiological characteristics of respiratory syncytial virus and influenza virus associated hospitalization in urban Thai infants. J Med Assoc Thai.

[82] Suryadevara M., Cummings E., Bonville C.A. (2011). Viral etiology of acute febrile respiratory illnesses in hospitalized children younger than 24 months. Clin Pediatr.

[83] Wang K., Chalker V., Bermingham A., Harrison T., Mant D., Harnden A. (2011). Mycoplasma pneumoniae and respiratory virus infections in children with persistent cough in England: a retrospective analysis. Pediatr Infect Dis J.

[84] Zuccotti G., Dilillo D., Zappa A. (2011). Epidemiological and clinical features of respiratory viral infections in hospitalized children during the circulation of influenza virus A(H1N1) 2009. Influenza Other Respir Viruses.

[85] Esposito S., Daleno C., Prunotto G. (2013). Impact of viral infections in children with community-acquired pneumonia: results of a study of 17 respiratory viruses. Influenza Other Respir Viruses.

[86] Brand H.K., de Groot R., Galama J.M. (2012). Infection with multiple viruses is not associated with increased disease severity in children with bronchiolitis. Pediatr Pulmonol.

[87] Chatzopoulou E., Melidou A., Gioula G. (2012). Contribution of influenza viruses, human metapneumovirus and respiratory syncytial virus to acute respiratory infections in children in northern Greece, 2008 - 2010. East J Med.

[88] Choi S.H., Hong S.B., Ko G.B. (2012). Viral infection in patients with severe pneumonia requiring intensive care unit admission. Am J Respir Crit Care Med.

[89] Garcia-Garcia M.L., Calvo C., Pozo F., Villadangos P.A., Perez-Brena P., Casas I. (2012). Spectrum of respiratory viruses in children with community-acquired pneumonia. Pediatr Infect Dis J.

[90] Gorjipour H., Karimi A.E., Fahimzad A., Shiva F., Falah F., Shamshiri A.R. (2012).

[91] Hoffmann J., Rabezanahary H., Randriamarotia M. (2012). Viral and atypical bacterial etiology of acute respiratory infections in children under 5 years old living in a rural tropical area of Madagascar. PLoS One.

[92] Hombrouck A., Sabbe M., Van Casteren V. (2012). Viral aetiology of influenza-like illness in Belgium during the influenza A(H1N1)2009 pandemic. Eur J Clin Microbiol Infect Dis.

[93] Kadjo H.A., Ekaza E., Coulibaly D. (2013). Sentinel surveillance for influenza and other respiratory viruses in Côte d'Ivoire, 2003-2010. Influenza Other Respir Viruses.

[94] Kwofie T.B., Anane Y.A., Nkrumah B., Annan A., Nguah S.B., Owusu M. (2012). Respiratory viruses in children hospitalized for acute lower respiratory tract infection in Ghana. Virol J.

[95] Mansbach J.M., Piedra P.A., Teach S.J. (2012). Prospective multicenter study of viral etiology and hospital length of stay in children with severe bronchiolitis. Arch Pediatr Adolesc Med.

[96] Pierangeli A., Scagnolari C., Selvaggi C. (2012). Virological and clinical characterization of respiratory infections in children attending an emergency department during the first autumn-winter circulation of pandemic A (H1N1) 2009 influenza virus. Clin Microbiol Infect.

[97] Schlaudecker E.P., Heck J.P., Macintyre E.T. (2012). Etiology and seasonality of viral respiratory infections in rural Honduran children. Pediatr Infect Dis J.

[98] Suzuki A., Lupisan S., Furuse Y. (2012). Respiratory viruses from hospitalized children with severe pneumonia in the Philippines. BMC Infect Dis.

[99] Turner P., Turner C., Watthanaworawit W. (2013). Respiratory virus surveillance in hospitalised pneumonia patients on the Thailand-Myanmar border. BMC Infect Dis.

[100] Aamir U.B., Alam M.M., Sadia H., Zaidi S.S.Z., Kazi B.M. (2013). Molecular characterization of circulating Respiratory Syncytial Virus (RSV) genotypes in Gilgit Baltistan Province of Pakistan during 2011-2012 winter season. PLoS One.

[101] Alavi S.M., Makvandi M., Fard S.N., Alavi L. (2013). Human respiratory syncytial virus infection and its subgroups among the hospitalized young children with acute respiratory infection. Jundishapur J Microbiol.

[102] Ali A., Khowaja A.R., Bashir M.Z., Aziz F., Mustafa S., Zaidi A. (2013). Role of human metapneumovirus, influenza A virus and respiratory syncytial virus in causing WHO-defined severe pneumonia in children in a developing country. PLoS One.

[103] Bigogo G.M., Breiman R.F., Feikin D.R. (2013). Epidemiology of respiratory syncytial virus infection in rural and urban Kenya. J Infect Dis.

[104] Choudhary M.L., Anand S.P., Wadhwa B.S., Chadha M.S. (2013). Genetic variability of human respiratory syncytial virus in Pune, Western India. Infect Genet Evol.

[105] Enan K.A., Nabeshima T., Kubo T. (2013). Survey of causative agents for acute respiratory infections among patients in Khartoum- State, Sudan, 2010-2011. Virol J.

[106] Feikin D.R., Njenga M.K., Bigogo G. (2013). Viral and bacterial causes of severe acute respiratory illness among children aged less than 5 years in a high malaria prevalence area of western Kenya, 2007-2010. Pediatr Infect Dis J.

[107] Guerrier G., Goyet S., Chheng E.T. (2013). Acute viral lower respiratory tract infections in Cambodian children: clinical and epidemiologic characteristics. Pediatr Infect Dis J.

[108] Harada Y., Kinoshita F., Yoshida L.M. (2013). Does respiratory virus coinfection increases the clinical severity of acute respiratory infection among children infected with respiratory syncytial virus?. Pediatr Infect Dis J.

[109] Huang G., Yu D., Mao N. (2013). Viral etiology of acute respiratory infection in Gansu Province, China, 2011. PLoS One.

[110] Huo X., Fang B., Liu L. (2013). Clinical and epidemiologic characteristics of respiratory syncytial virus infection among children aged <5 years, Jingzhou City, China, 2011. J Infect Dis.

[111] Jafri H.S., Wu X., Makari D., Henrickson K.J. (2013). Distribution of respiratory syncytial virus subtypes A and B among infants presenting to the emergency department with lower respiratory tract infection or apnea. Pediatr Infect Dis J.

[112] Kim J.K., Jeon J.S., Kim J.W., Rheem I. (2013). Epidemiology of respiratory viral infection using multiplex rt-PCR in Cheonan, Korea (2006-2010). J Microbiol Biotechnol.

[113] Li H.X., Wei Q.D., Tan A.J., Wang L.Y. (2013). Epidemiological analysis of respiratory viral etiology for influenza-like illness during 2010 in Zhuhai, China. Virol J.

[114] Miyaji Y., Kobayashi M., Sugai K. (2013). Severity of respiratory signs and symptoms and virus profiles in Japanese children with acute respiratory illness. Microbiol Immunol.

[115] Miller E.K., Gebretsadik T., Carroll K.N. (2013). Viral etiologies of infant bronchiolitis, croup and upper respiratory illness during 4 consecutive years. Pediatr Infect Dis J.

[116] Nakoune E., Tricou V., Manirakiza A. (2013). First introduction of pandemic influenza A/H1N1 and detection of respiratory viruses in pediatric patients in Central African Republic. Virol J.

[117] Naorat S., Chittaganpitch M., Thamthitiwat S. (2013). Hospitalizations for acute lower respiratory tract infection due to respiratory syncytial virus in Thailand, 2008-2011. J Infect Dis.

[118] Nikfar R., Shamsizadeh A., Makvandi M., Khoshghalb A. (2013). Detection of Respiratory syncytial virus in hospitalized children with acute lower respiratory tract infections, using RT PCR in Ahvaz, Iran. Arch Pediatr Infect Dis.

[119] Ohno A., Suzuki A., Lupisan S. (2013). Genetic characterization of human respiratory syncytial virus detected in hospitalized children in the Philippines from 2008 to 2012. J Clin Virol.

[120] Tecu C., Mihai M.E., Alexandrescu V.I. (2013). Single and multipathogen viral infections in hospitalized children with acute respiratory infections. Roum Arch Microbiol Immunol.

[121] Zhang C., Zhu N., Xie Z. (2013). Viral etiology and clinical profiles of children with severe acute respiratory infections in China. PLoS One.

[122] Broor S., Dawood F.S., Pandey B.G. (2014). Rates of respiratory virus-associated hospitalization in children aged <5 years in rural northern India. J Infect.

[123] Hara M., Takao S., Shimazu Y., Nishimura T. (2014). Three-year study of viral etiology and features of febrile respiratory tract infections in Japanese pediatric outpatients. Pediatr Infect Dis J.

[124] He Y., Lin G.Y., Wang Q. (2014). A 3-year prospective study of the epidemiology of acute respiratory viral infections in hospitalized children in Shenzhen, China. Influenza Other Respir Viruses.

[125] Kool M., Monteny M., van Doornum G.J., Moll H.A., Berger M.Y. (2015). Respiratory virus infections in febrile children presenting to a general practice out-of-hours service. Eur J Gen Pract.

[126] Kono J., Jonduo M.H., Omena M., Siba P.M., Horwood P.F. (2014). Viruses associated with influenza-like-illnesses in Papua New Guinea, 2010. J Med Virol.

[127] Lekana-Douki S.E., Nkoghe D., Drosten C., Ngoungou E.B., Drexler J.F., Leroy E.M. (2014). Viral etiology and seasonality of influenza-like illness in Gabon, March 2010 to June 2011. BMC Infect Dis.

[128] Shatizadeh S., Yavarian J., Rezaie F., Mahmoodi M., Naseri M., Azad T.M. (2014). Epidemiological and clinical evaluation of children with respiratory virus infections. Med J Islam Repub Iran.

[129] Al-Ayed M.S., Asaad A.M., Qureshi M.A., Ameen M.S. (2014). Viral etiology of respiratory infections in children in southwestern Saudi Arabia using multiplex reverse-transcriptase polymerase chain reaction. Saudi Med J.

[130] Balmaks R., Ribakova I., Gardovska D., Kazaks A. (2014). Molecular epidemiology of human respiratory syncytial virus over three consecutive seasons in Latvia. J Med Virol.

[131] Cai X.Y., Wang Q., Lin G.Y. (2014). Respiratory virus infections among children in South China. J Med Virol.

[132] Faghihloo E., Yavarian J., Jandaghi N.Z., Shadab A., Azad T.M. (2014). Genotype circulation pattern of human respiratory syncytial virus in Iran. Infect Genet Evol.

[133] Feng L., Li Z., Zhao S. (2014). Viral etiologies of hospitalized acute lower respiratory infection patients in China, 2009-2013. PLoS One.

[134] Gooskens J., van der Ploeg V., Sukhai R.N., Vossen A., Claas E.C.J., Kroes A.C.M. (2014). Clinical evaluation of viral acute respiratory tract infections in children presenting to the emergency department of a tertiary referral hospital in the Netherlands. BMC Pediatr.

[135] Martins Júnior R.B., Carney S., Goldemberg D. (2014). Detection of respiratory viruses by real-time polymerase chain reaction in outpatients with acute respiratory infection. Mem Inst Oswaldo Cruz.

[136] Kaida A., Kubo H., Takakura K. (2014). Associations between co-detected respiratory viruses in children with acute respiratory infections. Jpn J Infect Dis.

[137] Karadag-Oncel E., Ciblak M.A., Ozsurekci Y., Badur S., Ceyhan M. (2014). Viral etiology of influenza-like illnesses during the influenza season between December 2011 and April 2012. J Med Virol.

[138] Liu W.K., Liu Q., Chen D.H. (2014). Epidemiology of acute respiratory infections in children in Guangzhou: a three-year study. PLoS One.

[139] Liu J., Mu Y., Dong W. (2014). Genetic variation of human respiratory syncytial virus among children with fever and respiratory symptoms in Shanghai, China, from 2009 to 2012. Infect Genet Evol.

[140] Obodai E., Asmah R., Boamah I., Goka B., Odoom J.K., Adiku T. (2014). Respiratory syncytial virus genotypes circulating in urban Ghana: February to November 2006. Pan Afr Med J.

[141] Panayiotou C., Richter J., Koliou M., Kalogirou N., Georgiou E., Christodoulou C. (2014). Epidemiology of respiratory syncytial virus in children in Cyprus during three consecutive winter seasons (2010-2013): age distribution, seasonality and association between prevalent genotypes and disease severity. Epidemiol Infect.

[142] Pourakbari B., Mahmoudi S., Movahedi Z. (2014). Viral etiology of acute lower respiratory tract infections in hospitalized young children in a children's referral hospital in Iran. Turk J Pediatr.

[143] Radin J.M., Hawksworth A.W., Kammerer P.E. (2014). Epidemiology of pathogen-specific respiratory infections among three US populations. PLoS One.

[144] Schulert G.S., Hain P.D., Williams D.J. (2014). Utilization of viral molecular diagnostics among children hospitalized with community acquired pneumonia. Hosp Pediatr.

[145] Singh A.K., Jain A., Jain B. (2014). Viral aetiology of acute lower respiratory tract illness in hospitalised paediatric patients of a tertiary hospital: one year prospective study. Indian J Med Microbiol.

[146] del Valle Mendoza J., Cornejo-Tapia A., Weilg P. (2015). Incidence of respiratory viruses in peruvian children with acute respiratory infections. J Med Virol.

[147] Moattari A., Aleyasin S., Emami A., Fyruzi M., Pirbonyeh N. (2015). The prevalence of human metapneumovirus and respiratory syncytial virus and coinfection with both in hospitalized children with acute respiratory infection in South of Iran. Arch Pediatr Infect Dis.

[148] Aydemir Y., Aydemir O., Pekcan S., Ozdemir M. (2017). Value of multiplex PCR to determine the bacterial and viral aetiology of pneumonia in school-age children. Paediatr Int Child Health.

[149] Berce V., Unuk S., Duh D., Homsak M., Vicic M. (2015). Clinical and laboratory characteristics of viral lower respiratory tract infections in preschool children. Wien Klin Wochenschr.

[150] Cebey-López M., Herberg J., Pardo-Seco J. (2015). Viral co-infections in pediatric patients hospitalized with lower tract acute respiratory infections. PLoS One.

[151] Cui B., Zhang D., Pan H. (2015). Viral aetiology of acute respiratory infections among children and associated meteorological factors in southern China. BMC Infect Dis.

[152] Diaz J., Morales-Romero J., Perez-Gil G. (2015). Viral coinfection in acute respiratory infection in Mexican children treated by the emergency service: a cross-sectional study. Ital J Pediatr.

[153] Fu Y.F., Pan L.F., Sun Q. (2015). The clinical and etiological characteristics of influenza-like illness (ILI) in outpatients in Shanghai, China, 2011 to 2013. PLoS One.

[154] Halasa N., Williams J., Faouri S. (2015). Natural history and epidemiology of respiratory syncytial virus infection in the Middle East: hospital surveillance for children under age two in Jordan. Vaccine.

[155] Lagare A., Mainassara H.B., Issaka B., Sidiki A., Tempia S. (2015). Viral and bacterial etiology of severe acute respiratory illness among children < 5 years of age without influenza in Niger. BMC Infect Dis.

[156] Lee C.Y., Chang Y.F., Lee C.L. (2015). Molecular viral epidemiology and clinical characterization of acute febrile respiratory infections in hospitalized children in Taiwan. J Med Virol.

[157] Malasao R., Okamoto M., Chaimongkol N. (2015). Molecular characterization of human respiratory syncytial virus in the Philippines, 2012-2013. PLoS One.

[158] Martinez-Roig A., Salvado M., Caballero-Rabasco M.A., Sanchez-Buenavida A., Lopez-Segura N., Bonet-Alcaina M. (2015). Viral coinfection in childhood respiratory tract infections. Arch Bronconeumol.

[159] Othman H.T., Abu Elhamed W.A., Hassan D.M., Soliman M.S., Abdel Baset R.W. (2016). Respiratory syncytial virus and human metapneumovirus in severe lower respiratory tract infections in children under two. J Infect Dev Ctries.

[160] Ren L., Xiao Q., Zhou L., Xia Q., Liu E. (2015). Molecular characterization of human respiratory syncytial virus subtype B: a novel genotype of subtype B circulating in China. J Med Virol.

[161] Simusika P., Bateman A.C., Theo A. (2015). Identification of viral and bacterial pathogens from hospitalized children with severe acute respiratory illness in Lusaka, Zambia, 2011-2012: a cross-sectional study. BMC Infect Dis.

[162] Tuan T.A., Thanh T.T., Hai N. (2015). Characterization of hospital and community-acquired respiratory syncytial virus in children with severe lower respiratory tract infections in Ho Chi Minh City, Vietnam, 2010. Influenza Other Respir Viruses.

[163] Wei L., Liu W., Zhang X.A. (2015). Detection of viral and bacterial pathogens in hospitalized children with acute respiratory illnesses, Chongqing, 2009-2013. Medicine.

[164] Wertheim H.F.L., Nadjm B., Thomas S. (2015). Viral and atypical bacterial aetiologies of infection in hospitalised patients admitted with clinical suspicion of influenza in Thailand, Vietnam and Indonesia. Influenza Other Respir Viruses.

[165] Yu X., Kou Y., Xia D. (2015). Human respiratory syncytial virus in children with lower respiratory tract infections or influenza-like illness and its co-infection characteristics with viruses and atypical bacteria in Hangzhou, China. J Clin Virol.

[166] Zhang T.G., Li A.H., Lyu M., Chen M., Huang F., Wu J. (2015). Detection of respiratory viral and bacterial pathogens causing pediatric community-acquired pneumonia in Beijing using real-time PCR. Chronic Dis Transl Med.

[167] Wishaupt J.O., van den Berg E.A., van Wijk T., van der Ploeg T., Versteegh F.G., Hartwig N.G. (2016). Paediatric apnoeas are not related to a specific respiratory virus, and parental reports predict hospitalisation. Acta Paediatr.

[168] Richter J., Panayiotou C., Tryfonos C. (2016). Aetiology of acute respiratory tract infections in hospitalised children in Cyprus. PLoS One.

[169] Ali A., Akhund T., Warraich G.J. (2016). Respiratory viruses associated with severe pneumonia in children under 2 years old in a rural community in Pakistan. J Med Virol.

[170] Amer H.M., Alshaman M.S., Farrag M.A., Hamad M.E., Alsaadi M.M., Almajhdi F.N. (2016). Epidemiology of 11 respiratory RNA viruses in a cohort of hospitalized children in Riyadh, Saudi Arabia. J Med Virol.

[171] Antón A., Marcos M.A., Torner N. (2016). Virological surveillance of influenza and other respiratory viruses during six consecutive seasons from 2006 to 2012 in Catalonia, Spain. Clin Microbiol Infect.

[172] Bimouhen A., El Falaki F., Ihazmad H., Regragui Z., Benkerroum S., Barakat A. (2016). Circulation of Respiratory Syncytial Virus in Morocco during 2014-2016: findings from a sentinel-based virological surveillance system for influenza. East Mediterr Health J.

[173] Chou C.A., Lin T.I., Chen Y.S. (2016). Comparisons of etiology and diagnostic tools of lower respiratory tract infections in hospitalized young children in Southern Taiwan in two seasons. J Microbiol Immunol Infect.

[174] Cangiano G., Nenna R., Frassanito A. (2016). Bronchiolitis: analysis of 10 consecutive epidemic seasons. Pediatr Pulmonol.

[175] Do L.A.H., Bryant J.E., Tran A.T. (2016). Respiratory syncytial virus and other viral infections among children under two years old in Southern Vietnam 2009-2010: clinical characteristics and disease severity. PLoS One.

[176] Dong W., Chen Q., Hu Y. (2016). Epidemiological and clinical characteristics of respiratory viral infections in children in Shanghai, China. Arch Virol.

[177] Dut R., Kocagöz S. (2017). Respiratory viral test results in children. Cocuk Enf Derg.

[178] Faber T.E., Schuurs T.A., Veeger N.J., Hennus M.P., Bont L.J. (2016). Dynamics of nasopharyngeal pneumococcal carriage during the course of viral bronchiolitis. Pediatr Pulmonol.

[179] Fall A., Dia N., Cisse E. (2016). Epidemiology and molecular characterization of human respiratory syncytial virus in Senegal after four consecutive years of surveillance, 2012-2015. PLoS One.

[180] Girit S., Karaaslan A., Gencer S. (2018). Active surveillance of influenza A and other respiratory viruses in children with influenza-like-illness in two seasons. J Infect Dev Ctries.

[181] Goktas S., Sirin M.C. (2016). Prevalence and seasonal distribution of respiratory viruses during the 2014 - 2015 season in Istanbul. Jundishapur J Microbiol.

[182] Gurgel R.Q., Bezerra P.G.D., Duarte M. (2016). Relative frequency, possible risk factors, viral codetection rates, and seasonality of respiratory syncytial virus among children with lower respiratory tract infection in Northeastern Brazil. Medicine.

[183] Hu P., Zheng T., Chen J. (2017). Alternate circulation and genetic variation of human respiratory syncytial virus genotypes in Chengdu, West China, 2009-2014. J Med Virol.

[184] Kenmoe S., Tchendjou P., Vernet M.A. (2016). Viral etiology of severe acute respiratory infections in hospitalized children in Cameroon, 2011-2013. Influenza Other Respir Viruses.

[185] Karppinen S., Toivonen L., Schuez-Havupalo L., Waris M., Peltola V. (2016). Interference between respiratory syncytial virus and rhinovirus in respiratory tract infections in children. Clin Microbiol Infect.

[186] Liu W., Chen D., Tan W. (2016). Epidemiology and clinical presentations of respiratory syncytial virus subgroups A and B detected with multiplex real-time PCR. PLoS One.

[187] Malhotra B., Swamy M.A., Reddy P.V.J., Gupta M.L. (2016). Viruses causing severe acute respiratory infections (SARI) in children <= 5 years of age at a tertiary care hospital in Rajasthan, India. Indian J Med Res.

[188] Meligy B., Sayed A., Ismail D.K., Kamal D., Abdel-Latif W., Erfan D.M. (2016). Detection of viral acute lower respiratory tract infection in hospitalized infants using real-time PCR. Gaz Egypt Paediatr Assoc.

[189] Meskill S.D., Revell P.A., Chandramohan L., Cruz A.T. (2017). Prevalence of co-infection between respiratory syncytial virus and influenza in children. Am J Emerg Med.

[190] Mishra P., Nayak L., Das R.R., Dwibedi B., Singh A. (2016). Viral agents causing acute respiratory infections in children under five: a Study from Eastern India. Int J Pediatr.

[191] Moesker F.M., van Kampen J.J.A., van Rossum A.M.C. (2016). Viruses as sole causative agents of severe acute respiratory tract infections in children. PLoS One.

[192] Nyawanda B.O., Mott J.A., Njuguna H.N. (2016). Evaluation of case definitions to detect respiratory syncytial virus infection in hospitalized children below 5 years in Rural Western Kenya, 2009-2013. BMC Infect Dis.

[193] Panda S., Mohakud N.K., Suar M., Kumar S. (2017). Etiology, seasonality, and clinical characteristics of respiratory viruses in children with respiratory tract infections in Eastern India (Bhubaneswar, Odisha). J Med Virol.

[194] Parsania M., Poopak B., Pouriayevali M.H., Haghighi S., Amirkhani A., Nateghian A. (2016). Detection of human metapneumovirus and respiratory syncytial virus by real-time polymerase chain reaction among hospitalized young children in Iran. Jundishapur J Microbiol.

[195] Reeves R.M., Hardelid P., Gilbert R. (2016). Epidemiology of laboratory-confirmed respiratory syncytial virus infection in young children in England, 2010-2014: the importance of birth month. Epidemiol Infect.

[196] Slovic A., Ivancic-Jelecki J., Ljubin-Sternak S., Galinovic G.M., Forcic D. (2016). A molecular epidemiological study of human respiratory syncytial virus in Croatia, 2011-2014. Infect Genet Evol.

[197] Wang D., Chen L., Ding Y. (2016). Viral etiology of medically attended influenza-like illnesses in children less than five years old in Suzhou, China, 2011-2014. J Med Virol.

[198] Arbefeville S., Ferrieri P. (2017). Epidemiologic analysis of respiratory viral infections mainly in hospitalized children and adults in a Midwest University Medical Center after the implementation of a 14-virus multiplex nucleic acid amplification test. Am J Clin Pathol.

[199] Abdulhaq A.A., Basode V.K., Hashem A.M. (2017). Patterns of human respiratory viruses and lack of MERS-Coronavirus in patients with acute upper respiratory tract infections in Southwestern province of Saudi Arabia. Adv Virol.

[200] Benet T., Sánchez Picot V., Messaoudi M. (2017). Microorganisms associated with pneumonia in children < 5 years of age in developing and emerging countries: the GABRIEL pneumonia multicenter, prospective, case-control study. Clin Infect Dis.

[201] Avcu G., Bal Z.S., Cicek C., Vardar F. (2017). Clinical and epidemiological evaluation of hospitalized children with respiratory virus infections. J Pediatr Infect.

[202] Bashir U., Nisar N., Arshad Y. (2017). Respiratory syncytial virus and influenza are the key viral pathogens in children <2 years hospitalized with bronchiolitis and pneumonia in Islamabad Pakistan. Arch Virol.

[203] Bedolla-Barajas M., Montero H., Morales-Romero J. (2017). Prevalence of respiratory viruses in wheezing children not older than 24 months of age. Gac Med Mex.

[204] Bhuyan G.S., Hossain M.A., Sarker S.K. (2017). Bacterial and viral pathogen spectra of acute respiratory infections in under-5 children in hospital settings in Dhaka city. PLoS One.

[205] Brini I., Guerrero A., Hannachi N. (2017). Epidemiology and clinical profile of pathogens responsible for the hospitalization of children in Sousse area, Tunisia. PLoS One.

[206] Dang J.L., Zhao J.J. (2017). Viral respiratory tract infections and their correlation with clinical presentations and outcomes among young children attending emergency department of tertiary care hospital in China. Bio Res India.

[207] Fagbo S.F., Garbati M.A., Hasan R. (2017). Acute viral respiratory infections among children in MERS-endemic Riyadh, Saudi Arabia, 2012-2013. J Med Virol.

[208] Gökçe Ş., Kurugöl Z., Koturoğlu G., Çiçek C., Aslan A. (2017). Etiology, seasonality, and clinical features of viral respiratory tract infections in children hospitalized with acute bronchiolitis: a single-center study. Glob Pediatr Health.

[209] Janahi I., Abdulkayoum A., Almeshwesh F., Alkuwari M., Al Hammadi A., Alameri M. (2017). Viral aetiology of bronchiolitis in hospitalised children in Qatar. BMC Infect Dis.

[210] Jonnalagadda S., Rodriguez O., Estrella B., Sabin L.L., Sempertegui F., Hamer D.H. (2017). Etiology of severe pneumonia in Ecuadorian children. PLoS One.

[211] Kim Y.S., Kim K.R., Kang J.M., Kim J.M., Kim Y.J. (2017). Etiology and clinical characteristics of fever of unknown origin in children: a 15-year experience in a single center. Korean J Pediatr.

[212] Korsun N., Angelova S., Tzotcheva I. (2017). Prevalence and genetic characterisation of respiratory syncytial viruses circulating in Bulgaria during the 2014/15 and 2015/16 winter seasons. Pathog Glob Health.

[213] Lim F.J., Wake Z.V., Levy A. (2017). Viral etiology and the impact of codetection in young children presenting with influenza-like illness. J Pediatric Infect Dis Soc.

[214] Moe N., Krokstad S., Stenseng I.H. (2017). Comparing human metapneumovirus and respiratory syncytial virus: viral co-detections, genotypes and risk factors for severe disease. PLoS One.

[215] Nenna R., Evangelisti M., Frassanito A. (2017). Respiratory syncytial virus bronchiolitis, weather conditions and air pollution in an Italian urban area: an observational study. Environ Res.

[216] Nguyen V.H., Dubot-Peres A., Russell F.M. (2017). Acute respiratory infections in hospitalized children in Vientiane, Lao PDR - the importance of Respiratory Syncytial Virus. Sci Rep.

[217] O'Grady K.A.F., Grimwood K., Sloots T.P. (2017). Upper airway viruses and bacteria and clinical outcomes in children with cough. Pediatr Pulmonol.

[218] Pale M., Nacoto A., Tivane A. (2017). Respiratory syncytial and influenza viruses in children under 2 years old with severe acute respiratory infection (SARI) in Maputo, 2015. PLoS One.

[219] Park E., Park P.H., Huh J.W. (2017). Molecular and clinical characterization of human respiratory syncytial virus in South Korea between 2009 and 2014. Epidemiol Infect.

[220] Petrarca L., Nenna R., Frassanito A. (2018). Acute bronchiolitis: influence of viral co-infection in infants hospitalized over 12 consecutive epidemic seasons. J Med Virol.

[221] Piralla A., Mariani B., Rovida F., Baldanti F. (2017). Frequency of respiratory viruses among patients admitted to 26 Intensive Care units in seven consecutive winter-spring seasons (2009–2016) in Northern Italy. J Clin Virol.

[222] Sahu M., Shukla M.K., Barde P.V. (2017). Molecular characterization of human respiratory syncytial virus detected from central India. J Med Virol.

[223] Saxena S., Singh D., Zia A. (2017). Clinical characterization of influenza A and human respiratory syncytial virus among patients with influenza like illness. J Med Virol.

[224] Swamy M.A., Malhotra B., Reddy P.V., Tiwari J.K., Kumar N., Gupta M.L. (2017). Trends of respiratory syncytial virus sub-types in children hospitalised at a tertiary care centre in Jaipur during 2012-2014. Indian J Med Microbiol.

[225] Taylor S., Lopez P., Weckx L. (2017). Respiratory viruses and influenza-like illness: epidemiology and outcomes in children aged 6 months to 10 years in a multi-country population sample. J Infect.

[226] Thongpan I., Mauleekoonphairoj J., Vichiwattana P. (2017). Respiratory syncytial virus genotypes NA1, ON1, and BA9 are prevalent in Thailand, 2012-2015. PeerJ.

[227] Trenholme A.A., Best E.J., Vogel A.M., Stewart J.M., Miller C.J., Lennon D.R. (2017). Respiratory virus detection during hospitalisation for lower respiratory tract infection in children under 2 years in South Auckland, New Zealand. J Paediatr Child Health.

[228] Del Valle-Mendoza J., Silva-Caso W., Cornejo-Tapia A. (2017). Molecular etiological profile of atypical bacterial pathogens, viruses and coinfections among infants and children with community acquired pneumonia admitted to a national hospital in Lima, Peru. BMC Res Notes.

[229] Vieira S.E., Thomazelli L.M., De Paulis M. (2017). Infections caused by HRSV A ON1 are predominant among hospitalized infants with bronchiolitis in São Paulo City. BioMed Res Int.

[230] Wishaupt J.O., van der Ploeg T., de Groot R., Versteegh F.G., Hartwig N.G. (2017). Single- and multiple viral respiratory infections in children: disease and management cannot be related to a specific pathogen. BMC Infect Dis.

[231] Wollmeister E., Alvarez A.E., Bastos J.C.S. (2018). Respiratory syncytial virus in Brazilian infants - ten years, two cohorts. J Clin Virol.

[232] Wong-Chew R.M., Garcia-Leon M.L., Noyola D.E. (2017). Respiratory viruses detected in Mexican children younger than 5 years old with community-acquired pneumonia: a national multicenter study. Int J Infect Dis.

[233] Yan X.L., Li Y.N., Tang Y.J. (2017). Clinical characteristics and viral load of respiratory syncytial virus and human metapneumovirus in children hospitaled for acute lower respiratory tract infection. J Med Virol.

[234] Zheng Y., Liu L., Wang S. (2017). Prevailing genotype distribution and characteristics of human respiratory syncytial virus in northeastern China. J Med Virol.

[235] Swamy M.A., Malhotra B., Janardhan Reddy P.V., Tiwari J. (2018). Profile of respiratory pathogens causing acute respiratory infections in hospitalised children at Rajasthan a 4 year's study. Indian J Med Microbiol.

[236] Tine R.C., Ndiaye L.A., Niang M.N. (2018). Upper respiratory infections in a rural area with reduced malaria transmission in Senegal: a pathogens community study. BMC Infect Dis.

[237] Appak O., Duman M., Belet N., Sayiner A.A. (2019). Viral respiratory infections diagnosed by multiplex polymerase chain reaction in pediatric patients. J Med Virol.

[238] Assane D., Makhtar C., Abdoulaye D. (2018). Viral and bacterial etiologies of acute respiratory infections among children under 5 years in Senegal. Microbiol Insights.

[239] Aykaç K., Karadağ-Öncel E., Bayhan C. (2018). Prevalence and seasonal distribution of viral etiology of respiratory tract infections in inpatients and outpatients of the pediatric population: 10 year follow-up. Turk J Pediatr.

[240] Bhuiyan M.U., Snelling T.L., West R. (2019). The contribution of viruses and bacteria to community-acquired pneumonia in vaccinated children: a case-control study. Thorax.

[241] Canela L.N.P., Magalhães-Barbosa M.C.D., Raymundo C.E. (2018). Viral detection profile in children with severe acute respiratory infection. Braz J Infect Dis.

[242] Chen J.Y., Hu P.W., Zhou T. (2018). Epidemiology and clinical characteristics of acute respiratory tract infections among hospitalized infants and young children in Chengdu, West China, 2009-2014. BMC Pediatr.

[243] Chittaganpitch M., Waicharoen S., Yingyong T. (2018). Viral etiologies of influenza-like illness and severe acute respiratory infections in Thailand. Influenza Other Respir Viruses.

[244] Cieslak K., Kowalczyk D., Szymanski K., Hallmann-Szelinska E., Brydak L.B., Porkorski M. (2018). Clinical pulmonary research.

[245] Cowling B.J., Chan K.H., Peiris J.S. (2018). Influenza-like illness and viral aetiology in Hong Kong children. Hong Kong Med J.

[246] El Baroudy N.R., El Refay A.S., Abdel Hamid T.A., Hassan D.M., Soliman M.S., Sherif L. (2018). Respiratory viruses and atypical bacteria co-infection in children with acute respiratory infection. Open Access Maced J Med Sci.

[247] Famoroti T., Sibanda W., Ndung'u T. (2018). Prevalence and seasonality of common viral respiratory pathogens, including Cytomegalovirus in children, between 0-5 years of age in KwaZulu-Natal, an HIV endemic province in South Africa. BMC Pediatr.

[248] Fillatre A., Francois C., Segard C. (2018). Epidemiology and seasonality of acute respiratory infections in hospitalized children over four consecutive years (2012-2016). J Clin Virol.

[249] Fieldhouse J.K., Toh T.-H., Lim W.-H. (2018). Surveillance for respiratory syncytial virus and parainfluenza virus among patients hospitalized with pneumonia in Sarawak, Malaysia. PLoS One.

[250] Gaymard A., Bouscambert-Duchamp M., Pichon M. (2018). Genetic characterization of respiratory syncytial virus highlights a new BA genotype and emergence of the ON1 genotype in Lyon, France, between 2010 and 2014. J Clin Virol.

[251] Ge X., Guo Y., Chen J., Hu R., Feng X. (2018). Epidemiology and seasonality of respiratory viruses detected from children with respiratory tract infections in Wuxi, East China. Med Sci Monit.

[252] Gimferrer L., Vila J., Piñana M. (2019). Virological surveillance of human respiratory syncytial virus A and B at a tertiary hospital in Catalonia (Spain) during five consecutive seasons (2013-2018). Future Microbiol.

[253] Hassan D.A., Rachid S.K., Ziebuhr J. (2018). A single-center Study of viral respiratory tract infections in hospitalized children from the Kurdistan Region of Iraq. Glob Pediatr Health.

[254] Hendaus M.A., Alhammadi A.H., Chandra P., Muneer E., Khalifa M.S. (2018). Identifying agents triggering bronchiolitis in the State of Qatar. Int J Gen Med.

[255] Hindupur A., Menon T., Dhandapani P. (2019). Epidemiology of respiratory syncytial virus infections in Chennai, South India. Clin Epidemiol Glob Health.

[256] Kadjo H.A., Adjogoua E., Dia N. (2018). Detection of non-influenza viruses in acute respiratory infections in children under five-year-old in Cote d’ivoire (January – December 2013). Afr J Infect Dis.

[257] Kabego L., Balol'Ebwami S., Kasengi J.B. (2018). Human respiratory syncytial virus: prevalence, viral co-infections and risk factors for lower respiratory tract infections in children under 5 years of age at a general hospital in the democratic republic of Congo. J Med Microbiol.

[258] Brini Khalifa I., Hannachi N., Guerrero A. (2019). Demographic and seasonal characteristics of respiratory pathogens in neonates and infants aged 0 to 12 months in the central-east region of Tunisia. J Med Virol.

[259] Kurskaya O., Ryabichenko T., Leonova N. (2018). Viral etiology of acute respiratory infections in hospitalized children in Novosibirsk City, Russia (2013 - 2017). PLoS One.

[260] Li X., Li J., Meng L. (2018). Viral etiologies and epidemiology of patients with acute respiratory infections based on sentinel hospitals in Gansu Province, Northwest China, 2011-2015. J Med Virol.

[261] Li J., Tao Y., Tang M. (2018). Rapid detection of respiratory organisms with the FilmArray respiratory panel in a large children's hospital in China. BMC Infect Dis.

[262] Nascimento-Carvalho A.C., Vilas-Boas A.L., Fontoura M.S.H., Vuorinen T., Nascimento-Carvalho C.M., PNEUMOPAC-Efficacy Study Group (2018). Respiratory viruses among children with non-severe community-acquired pneumonia: a prospective cohort study. J Clin Virol.

[263] Nicholson E.G., Avadhanula V., Ferlic-Stark L., Patel K., Gincoo K.E., Piedra P.A. (2019). The risk of serious bacterial infection in febrile infants 0-90 days of life with a respiratory viral infection. Pediatr Infect Dis J.

[264] Obodai E., Odoom J.K., Adiku T. (2019). The significance of human respiratory syncytial virus (HRSV) in children from Ghana with acute lower respiratory tract infection: a molecular epidemiological analysis, 2006 and 2013-2014 (vol 13, e0203788, 2018). PLoS One.

[265] Ogunsemowo O., Olaleye D.O., Odaibo G.N. (2018). Human respiratory syncytial virus subtypes A and B infection among children attending primary and secondary health care facilities in Ibadan, Nigeria. Arch Basic Appl Med.

[266] Okamoto M., Dapat C.P., Sandagon A.M.D. (2018). Molecular characterization of respiratory syncytial virus in children with repeated infections with subgroup B in the Philippines. J Infect Dis.

[267] Rashid Z.Z., Lee P.C., Ali U.K., Najihan M., Samat A., Tang S.F. (2018). Multiplex real-time PCR detection of respiratory viruses in lower respiratory tract infections in children. Sains Malays.

[268] Ravindranath T.M., Gomez A., Harwayne-Gidansky I. (2018). Pediatric acute respiratory distress syndrome associated with human metapneumovirus and respiratory syncytial virus. Pediatr Pulmonol.

[269] Razanajatovo N.H., Guillebaud J., Harimanana A. (2018). Epidemiology of severe acute respiratory infections from hospital-based surveillance in Madagascar, November 2010 to July 2013. PLoS One.

[270] Snoeck C.J., Ponghsavath V., Luetteke N. (2018). Etiology of viral respiratory infections in Northern Lao People'S Democratic Republic. J Med Virol.

[271] Tsagarakis N.J., Sideri A., Makridis P., Triantafyllou A., Stamoulakatou A., Papadogeorgaki E. (2018). Age-related prevalence of common upper respiratory pathogens, based on the application of the FilmArray Respiratory panel in a tertiary hospital in Greece. Medicine.

[272] Yu J., Xie Z., Zhang T. (2018). Comparison of the prevalence of respiratory viruses in patients with acute respiratory infections at different hospital settings in North China, 2012-2015. BMC Infect Dis.

[273] Mackenzie G.A., Vilane A., Salaudeen R. (2019). Respiratory syncytial, parainfluenza and influenza virus infection in young children with acute lower respiratory infection in rural Gambia. Sci Rep.

[274] Liu W.K., Chen D.H., Tan W.P. (2019). Paramyxoviruses respiratory syncytial virus, parainfluenza virus, and human metapneumovirus infection in pediatric hospitalized patients and climate correlation in a subtropical region of southern China: a 7-year survey. Eur J Clin Microbiol Infect Dis.

[275] Tokak S., Gulseren Y.D., Ozdemir M. (2019). Determination of epidemiology and seasonal distribution of viral agents detected in children with respiratory tract infection. J Pediatr Inf.

[276] Abduljabbar H.L., Hussein A.A., Al-Mayah Q.S., Aufi I.M. (2019). Al-Galiby QH, Al-Galiby QH.

[277] Alharbiaburiziza S. (2019). Clinical outcomes of lower respiratory tract infections: an epidemiological study comparing viral and non-viral lower respiratory tract infections in Jeddah. J Med Microb Diagn.

[278] Barlotta A., Pirillo P., Stocchero M. (2019). Metabolomic profiling of infants with recurrent wheezing after bronchiolitis. J Infect Dis.

[279] Bekhof J., Wessels M., Ten Velde E. (2019). Room sharing in hospitalized children with bronchiolitis and the occurrence of hospital-acquired infections: a prospective cohort study. Hosp Pediatr.

[280] Derrar F., Izri K., Kaddache C., Boukari R., Hannoun D. (2019). Virologic study of acute lower respiratory tract infections in children admitted to the paediatric department of Blida University Hospital, Algeria. New Microbes New Infect.

[281] Etemadi M.R., Jalilian F.A., Othman N. (2019). Diversity of respiratory viruses detected among hospitalized children with acute lower respiratory tract infections at Hospital Serdang, Malaysia. J Virol Methods.

[282] Halaji M., Hashempour T., Moayedi J. (2019). Viral etiology of acute respiratory infections in children in Southern Iran. Rev Soc Bras Med Trop.

[283] Harun A., Beyza E. (2019). Viral and atypical bacterial respiratory infections in a university teaching hospital. Jpn J Infect Dis.

[284] Hasegawa K., Goto T., Hirayama A. (2019). Respiratory virus epidemiology among us infants with severe bronchiolitis: analysis of 2 multicenter, multiyear cohort studies. Pediatr Infect Dis J.

[285] Mohamed S.A., Hatem A., Abuelhassan U., Rizk M.S., El-kholy A., Al-Harras M. (2019). Viral and atypical bacterial etiologies of severe acute respiratory infection (SARI) in Egyptian patients: epidemiological patterns and results from the sentinel surveillance study 2010-2014. Egypt J Chest Dis Tuberc.

[286] Hindupur A., Menon T., Dhandapani P. (2019). Genetic diversity of human respiratory syncytial virus in children with acute respiratory infections in Chennai, South India. Indian J Med Microbiol.

[287] Knobbe R.B., Diallo A., Fall A. (2019). Pathogens causing respiratory tract infections in children less than 5 years of age in Senegal. Microbiol Insights.

[288] Korsun N., Angelova S., Trifonova I. ([publication of the Brazilian Society for Microbiology] 2019). Viral pathogens associated with acute lower respiratory tract infections in children younger than 5 years of age in Bulgaria. Braz J Microbiol.

[289] Lagare A., Ousmane S., Dano I.D. (2019). Molecular detection of respiratory pathogens among children aged younger than 5 years hospitalized with febrile acute respiratory infections: a prospective hospital-based observational study in Niamey, Niger. Health Sci Rep.

[290] Wang L., Yang S., Yan X., Liu T., Feng Z., Li G. (2019). Comparing the yield of oropharyngeal swabs and sputum for detection of 11 common pathogens in hospitalized children with lower respiratory tract infection. Virol J.

[291] Li Y.T., Liang Y., Ling Y.S., Duan M.Q., Pan L., Chen Z.G. (2019). The spectrum of viral pathogens in children with severe acute lower respiratory tract infection: a 3-year prospective study in the pediatric intensive care unit. J Med Virol.

[292] McCallum G., Grimwood K., Oguoma V. (2019). The point prevalence of respiratory syncytial virus in hospital and community-based studies in children from Northern Australia: studies in a 'high-risk' population. Eur Respir J.

[293] Midulla F., Nenna R., Scagnolari C. (2019). How respiratory syncytial virus genotypes influence the clinical course in infants hospitalized for bronchiolitis. J Infect Dis.

[294] Rha B., Dahl R.M., Moyes J. (2019). Performance of surveillance case definitions in detecting respiratory syncytial virus infection among young children hospitalized with severe respiratory illness-south Africa, 2009-2014. J Pediatric Infect Dis Soc.

[295] Sáez-López E., Pechirra P., Costa I. (2019). Performance of surveillance case definitions for respiratory syncytial virus infections through the sentinel influenza surveillance system, Portugal, 2010 to 2018. Euro Surveill.

[296] Sonawane A.A., Shastri J., Bavdekar S.B. (2019). Respiratory pathogens in infants diagnosed with acute lower respiratory tract infection in a tertiary care Hospital of Western India using multiplex real time PCR. Indian J Pediatr.

[297] Thongpan I., Suntronwong N., Vichaiwattana P., Wanlapakorn N., Vongpunsawad S., Poovorawan Y. (2019). Respiratory syncytial virus, human metapneumovirus, and influenza virus infection in Bangkok, 2016-2017. PeerJ.

[298] Wen S., Lv F., Chen X. (2019). Application of a nucleic acid-based multiplex kit to identify viral and atypical bacterial aetiology of lower respiratory tract infection in hospitalized children. J Med Microbiol.

[299] Toh T.H., Hii K.C., Fieldhouse J.K. (2019). High prevalence of viral infections among hospitalized pneumonia patients in equatorial Sarawak, Malaysia. Open Forum Infect Dis.

[300] Wilson P.T., Baiden F., Brooks J.C. (2019). Respiratory pathogens in children 1 month to 5 years of age presenting with undifferentiated acute respiratory distress in 2 district-level hospitals in Ghana. J Pediatric Infect Dis Soc.

[301] Xu K., Huo X., Zu R. (2019). Etiological characteristics of influenza-like illness in Jiangsu province from 2012 to 2016. J Biomed Res.

[302] Yen C.Y., Wu W.T., Chang C.Y. (2019). Viral etiologies of acute respiratory tract infections among hospitalized children – a comparison between single and multiple viral infections. J Microbiol Immunol Infect.

[303] Yew S.M., Tan K.L., Yeo S.K., Ng K.P., Kuan C.S. (2019). Molecular epidemiology of respiratory viruses among Malaysian young children with a confirmed respiratory infection during 2014–2015. J Thorac Dis.

[304] Yurtseven A., Turan C., Elibol P., Çiçek C., Saz E.U. (2019). Is multiple viral infection a predictor of severity in children with acute bronchiolitis?. Hong Kong J Emerg Med.

[305] Zhao Y.J., Lu R.J., Shen J., Xie Z.D., Liu G.S., Tan W.J. (2019). Comparison of viral and epidemiological profiles of hospitalized children with severe acute respiratory infection in Beijing and Shanghai, China. BMC Infect Dis.

[306] Vanderburg S., Wijayaratne G., Danthanarayana N. (2020). Outbreak of severe acute respiratory infection in Southern Province, Sri Lanka in 2018: a cross-sectional study. BMJ Open.

[307] Tsou P., Vadivelan A., Kovvuri M. (2020). Association between multiple respiratory viral infections and pediatric intensive care unit admission among infants with bronchiolitis. Arch Pediatr.

[308] Thongpan I., Vongpunsawad S., Poovorawan Y. (2020). Respiratory syncytial virus infection trend is associated with meteorological factors. Sci Rep.

[309] Sik G., Demirbuga A., Annayev A., Cabiri A., Deliceo E., Citak A. (2020). Viral infections among patients with acute lower respiratory tract infections in the pediatric intensive care unit. J Pediatr Inf.

[310] Pham H.T., Nguyen P.T.T., Tran S.T., Phung T.T.B. (2020). Clinical and pathogenic characteristics of lower respiratory tract infection treated at the Vietnam National Children's Hospital. Can J Infect Dis Med Microbiol.

[311] Gareca Perales J., Soleto Ortiz L., Loayza Mafayle R. (2021). Diagnosis of community-acquired pneumonia in hospitalized children: a multicenter experience in Bolivia. Pediatr Infect Dis J.

[312] Palani N., Sistla S. (2020). Epidemiology and phylogenetic analysis of respiratory viruses from 2012 to 2015 - a sentinel surveillance report from union territory of Puducherry, India. Clin Epidemiol Glob Health.

[313] Lin C.Y., Hwang D., Chiu N.C. (2020). Increased detection of viruses in children with respiratory tract infection using PCR. Int J Environ Res Public Health.

[314] Lee E., Kim C.H., Lee Y.J. (2020). Annual and seasonal patterns in etiologies of pediatric community-acquired pneumonia due to respiratory viruses and Mycoplasma pneumoniae requiring hospitalization in South Korea. BMC Infect Dis.

[315] Korsun N., Angelova S., Trifonova I. (2021). Predominance of ON1 and BA9 genotypes of respiratory syncytial virus (RSV) in Bulgaria, 2016-2018. J Med Virol.

[316] Karaarslan U., Topal S., Ayhan Y., Agin H. (2021). The differences in viral etiologies between children with and without severe disability admitted to the pediatric intensive care unit with acute respiratory illness. J Pediatr Infect Dis.

[317] Jarju S., Greenhalgh K., Wathuo M. (2020). Viral etiology, clinical features and antibiotic use in children <5 years of age in the Gambia presenting with influenza-like illness. Pediatr Infect Dis J.

[318] Huang X.B., Yuan L., Ye C.X. (2020). Epidemiological characteristics of respiratory viruses in patients with acute respiratory infections during 2009–2018 in southern China. Int J Infect Dis.

[319] Hattoufi K., Tligui H., Obtel M., El Ftouh S., Kharbach A., Barkat A. (2020). Molecular diagnosis of pneumonia using multiplex real-time PCR assay RespiFinder® SMART 22 FAST in a group of Moroccan infants. Adv Virol.

[320] Gao M., Yao X., Mao W. (2020). Etiological analysis of virus, mycoplasma pneumoniae and chlamydia pneumoniae in hospitalized children with acute respiratory infections in Huzhou. Virol J.

[321] Emanuels A., Hawes S.E., Newman K.L. (2020). Respiratory viral coinfection in a birth cohort of infants in rural Nepal. Influenza Other Respir Viruses.

[322] Duyu M., Karakaya Z. (2021). Viral etiology and outcome of severe lower respiratory tract infections among critically ill children admitted to the PICU. Med Intensiva.

[323] Chowdhury F., Shahid A.S.M.S.B., Ghosh P.K. (2020). Viral etiology of pneumonia among severely malnourished under-five children in an urban hospital, Bangladesh. PLoS One.

[324] Castro I.A., Costa L.D.C., Oliveira A.C.R. (2020). Circulation profile of respiratory viruses in symptomatic and asymptomatic children from Midwest Brazil. Braz J Microbiol.

[325] Calvo C., Alcolea S., Casas I. (2020). A 14-year prospective study of human coronavirus infections in hospitalized children comparison with other respiratory viruses. Pediatr Infect Dis J.

[326] Bunthi C., Rhodes J., Thamthitiwat S. (2021). Etiology and clinical characteristics of severe pneumonia among young children in Thailand: pneumonia etiology research for child health (PERCH) case-control study findings, 2012-2013. Pediatr Infect Dis J.

[327] Aygun D., Erbek F., Kuskucu M. (2020). The epidemiologic and clinical features of viral agents among hospitalized children with lower respiratory tract infections. Turk Pediatri Ars.

[328] Ang L., Mak T.M., Cui L., Leo Y.S., Lee V.J.M., Lin R.T.P. (2020). Characterisation of respiratory syncytial virus activity in children and adults presenting with acute respiratory illness at primary care clinics in Singapore, 2014-2018. Influenza Other Respir Viruses.

[329] Al-Romaihi H.E., Smatti M.K., Al-Khatib H.A. (2020). Molecular epidemiology of influenza, RSV, and other respiratory infections among children in Qatar: a six years report (2012-2017). Int J Infect Dis.

[330] Adema I.W., Kamau E., Uchi Nyiro J. (2020). Surveillance of respiratory viruses among children attending a primary school in rural coastal Kenya. Wellcome Open Res.

[331] Abinaya S., Gaspar B.L., Benjamin A.T. (2020). A study on aetiology and outcomes of viral lower respiratory tract infections in hospitalized children from South India. SriLanka J Child Health.

[332] Atay Ö., Pekcan S., Göktürk B., Özdemir M. (2020). Risk factors and clinical determinants in bronchiolitis of infancy. Turk Thorac J.

[333] Aamir U.B., Salman M., Nisar N. (2020). Molecular characterization of circulating respiratory syncytial virus genotypes in Pakistani children, 2010-2013. J Infect Public Health.

[334] Tsergouli K., Pappa S., Haidopoulou K., Gogou M., Giannopoulos A., Papa A. (2019). Respiratory syncytial virus in Greece, 2016–2018. Intervirology.

[335] Luo H.J., Huang X.B., Zhong H.L. (2020). Epidemiological characteristics and phylogenic analysis of human respiratory syncytial virus in patients with respiratory infections during 2011-2016 in southern China. Int J Infect Dis.

[336] Hasuwa T., Kinoshita F., Harada S. (2020). Viral etiology of acute lower respiratory tract infections in hospitalized children in Nagasaki, a regional city of Japan in 2013-2015. Pediatr Infect Dis J.

[337] Zhu Y., Xu B.P., Li C.C. (2021). A multicenter study of viral aetiology of community-acquired pneumonia in hospitalized children in Chinese Mainland. Virol Sin.

[338] Vianna L.A., Siqueira M.M., Volpini L.P.B., Louro I.D., Resende P.C. (2021). Seasonality, molecular epidemiology, and virulence of Respiratory Syncytial Virus (RSV): a perspective into the Brazilian Influenza Surveillance Program. PLoS One.

[339] Vasconcelos M.K., Loens K., Sigfrid L. (2021). Aetiology of acute respiratory infection in preschool children requiring hospitalisation in Europe-results from the PED-MERMAIDS multicentre case-control study. BMJ Open Respir Res.

[340] Thongpan I., Vichaiwattana P., Vongpunsawad S., Poovorawan Y. (2021). Upsurge of human rhinovirus infection followed by a delayed seasonal respiratory syncytial virus infection in Thai children during the coronavirus pandemic. Influenza Other Respir Viruses.

[341] Tavakoli F., Izadi A., Yavarian J., Sharifi-Zarchi A., Salimi V., Mokhtari-Azad T. (2021). Determination of genetic characterization and circulation pattern of Respiratory Syncytial Virus (RSV) in children with a respiratory infection, Tehran, Iran, during 2018-2019. Virus Res.

[342] Shutes B.L., Patel A.B., Moore-Clingenpeel M.D., Mejias A., Karsies T.J. (2021). Relationship of viral detection with duration of ventilation in critically ill infants with lower respiratory tract infection. Ann Am Thorac Soc.

[343] Snoeck C.J., Evdokimov K., Xaydalasouk K. (2021). Epidemiology of acute respiratory viral infections in children in Vientiane, Lao People's Democratic Republic. J Med Virol.

[344] Ramezannia Z., Sadeghi J., Abdoli Oskouie S. (2021). Evaluation of human respiratory syncytial virus and human parainfluenza virus type 3 among hospitalized children in Northwest of Iran. Can J Infect Dis Med Microbiol.

[345] Raju A.T., Das R., Rai N., Kumar A., Gaind R. (2021). A prospective study on respiratory viral pathogens causing acute lower respiratory infections in children below five years of age at a tertiary care hospital of India. J Clin Diagn Res.

[346] Mathisen M., Basnet S., Christensen A. (2021). Viral and atypical bacterial detection in young Nepalese children hospitalized with severe pneumonia. Microbiol Spectr.

[347] Mandelia Y., Procop G.W., Richter S.S., Worley S., Liu W., Esper F. (2021). Dynamics and predisposition of respiratory viral co-infections in children and adults. Clin Microbiol Infect.

[348] Lin C.X., Lian H.B., Lin G.Y. (2021). Pathogen spectrum changes of respiratory tract infections in children in Chaoshan area under the influence of COVID-19. Epidemiol Infect.

[349] Lim D.K., Jeon J.S., Jang T.S., Kim J.K. (2022). Association between climatic factors and respiratory syncytial virus detection rates in Cheonan, Korea. Environ Sci Pollut Res Int.

[350] Li L., Wang H., Liu A. (2021). Comparison of 11 respiratory pathogens among hospitalized children before and during the COVID-19 epidemic in Shenzhen, China. Virol J.

[351] Leli C., Di Matteo L., Gotta F. (2021). Prevalence of respiratory viruses by Multiplex PCR: a four-and-a-half year retrospective study in an Italian general hospital. Infez Med.

[352] Lei C., Yang L., Lou C.T. (2021). Viral etiology and epidemiology of pediatric patients hospitalized for acute respiratory tract infections in Macao: a retrospective study from 2014 to 2017. BMC Infect Dis.

[353] Komoyo G.F., Yambiyo B.M., Manirakiza A. (2021). Epidemiology and genetic characterization of respiratory syncytial virus in children with acute respiratory infections: findings from the influenza sentinel surveillance network in Central African Republic, 2015 to 2018. Health Sci Rep.

[354] Khomenko V.E., Iemets O.V., Volosovets O.P., Kryvopustov S.P., Kryvopustova M.V., Mozyrska O.V. (2021). Epidemiology of respiratory pathogens in children with acute respiratory tract infection in Ukraine during 2018-2020 years. Wiad Lek.

[355] Juliana A.E., Tang M.J., Kemps L. (2021). Viral causes of severe acute respiratory infection in hospitalized children and association with outcomes: a two-year prospective surveillance study in Suriname. PLoS One.

[356] Ihling C.M., Schnitzler P., Heinrich N. (2021). Molecular epidemiology of respiratory syncytial virus in children in Sub-Saharan Africa. Trop Med Int Health.

[357] Haddadin Z., Rankin D.A., Lipworth L. (2021). Respiratory virus surveillance in infants across different clinical settings. J Pediatr.

[358] Guo P., Wang Y., Zhou M., Mei S., Cheng Y. (2021). Detection and analysis of respiratory pathogens in hospitalized children with acute respiratory tract infections by a multiplex-PCR assay based on the genetic analyzer platform. Iran J Pediatr.

[359] El-Senousy W.M., Shouman M. (2021). Human coronavirus NL63 among other respiratory viruses in clinical specimens of Egyptian children and raw sewage samples. Food Environ Virol.

[360] Diesner-Treiber S.C., Voitl P., Voitl J.J.M. (2021). Respiratory infections in children during a Covid-19 pandemic winter. Front Pediatr.

[361] Correia W., Dorta-Guerra R., Sanches M. (2021). Study of the etiology of acute respiratory infections in children under 5 years at the Dr. Agostinho Neto Hospital, Praia, Santiago Island, Cabo Verde. Front Pediatr.

[362] Chen X., Zhu Y., Wang W. (2021). A multi-center study on molecular epidemiology of human respiratory syncytial virus from children with acute lower respiratory tract infections in the mainland of China between 2015 and 2019. Virol Sin.

[363] Arshad M., Saeed U., Ashraf M.I. (2021). Viral Origin of Wheeze in under-five population in Pakistan. Pak J Med Health Sci.

[364] Agca H., Akalin H., Saglik I., Hacimustafaoglu M., Celebi S., Ener B. (2021). Changing epidemiology of influenza and other respiratory viruses in the first year of COVID-19 pandemic. J Infect Public Health.

[365] Vittucci A.C., Piccioni L., Coltella L. (2021). The disappearance of respiratory viruses in children during the COVID-19 pandemic. Int J Environ Res Public Health.

[366] Zhang L.N., Cao L., Meng L.H. (2022). Pathogenic changes of community-acquired pneumonia in a children's hospital in Beijing, China before and after COVID-19 onset: a retrospective study. World J Pediatr.

[367] Yun K.W., Wallihan R., Desai A. (2022). Clinical characteristics and etiology of community-acquired pneumonia in US children, 2015-2018. Pediatr Infect Dis J.

[368] Xu M.H., Liu P.C., Su L.Y. (2022). Comparison of respiratory pathogens in children with lower respiratory tract infections before and during the COVID-19 pandemic in Shanghai, China. Front Pediatr.

[369] Xiang W.Q., Li L., Guo Y.J., Lin J., Li W. (2022). The impact of COVID-19 public health measures on detection of other respiratory viruses in children during the winter of 2020-2021 in Hangzhou, China. J Pediatr Infect Dis.

[370] Windsor W.J., Lamb M.M., Dominguez S.R., Mistry R.D., Rao S. (2022). Clinical characteristics and illness course based on pathogen among children with respiratory illness presenting to an emergency department. J Med Virol.

[371] Tabatabai J., Ihling C.M., Rehbein R.M. (2022). Molecular epidemiology of respiratory syncytial virus in hospitalised children in Heidelberg, Southern Germany, 2014-2017. Infect Genet Evol.

[372] Suryadevara M., Fajardo F.P., Aponte C.C. (2023). Etiologies of outpatient medically attended acute respiratory infections among young Ecuadorian children prior to the start of the 2020 SARS-CoV-2 pandemic. Influenza Other Respir Viruses.

[373] Savaş Şen Z., Vezir E., Erturk P. (2022). The relationship between viruses and clinical findings in hospitalized children diagnosed with acute lower respiratory tract infection. J Pediatr Inf.

[374] Sarkar S., Ratho R.K., Singh M., Singh M.P., Singh A., Sharma M. (2022). Comparative analysis of epidemiology, clinical features, and cytokine response of respiratory syncytial and human metapneumovirus infected children with acute lower respiratory infections. Jpn J Infect Dis.

[375] Gómez de la Torre Pretell J.C., Hueda-Zavaleta M., Cáceres-DelAguila J.A. (2022). Clinical characteristics associated with detected respiratory microorganism employing multiplex nested PCR in patients with presumptive COVID-19 but negative molecular results in Lima, Peru. Trop Med Infect Dis.

[376] Shen D.P., Vermeulen F., Debeer A., Lagrou K., Smits A. (2022). Impact of COVID-19 on viral respiratory infection epidemiology in young children: a single-center analysis. Front Public Health.

[377] Orquedaa A.S., Luciona M.F., Juareza M.D. (2022). Respiratory syncytial virus and influenza surveillance in schoolchildren seen at a children's hospital over 2 months of the second semester of 2021. Arch Argent Pediatr.

[378] Ogunbayo A.E., Mogotsi M.T., Sondlane H., Nkwadipo K.R., Sabiu S., Nyaga M.M. (2022). Pathogen profile of children hospitalised with severe acute respiratory infections during COVID-19 pandemic in the free state province, South Africa. Int J Environ Res Public Health.

[379] Nenna R., Matera L., Pierangeli A. (2022). First COVID-19 lockdown resulted in most respiratory viruses disappearing among hospitalised children, with the exception of rhinoviruses. Acta Paediatr.

[380] Moleleki M., du Plessis M., Ndlangisa K. (2022). Pathogens detected using a syndromic molecular diagnostic platform in patients hospitalized with severe respiratory illness in South Africa in 2017. Int J Infect Dis.

[381] Jiang M.L., Xu Y.P., Wu H. (2023). Changes in endemic patterns of respiratory syncytial virus infection in pediatric patients under the pressure of nonpharmaceutical interventions for COVID-19 in Beijing, China. J Med Virol.

[382] Meyer M., Ruebsteck E., Eifinger F. (2022). Morbidity of respiratory syncytial virus (RSV) infections: RSV compared with severe acute respiratory syndrome coronavirus 2 infections in children aged 0-4 years in Cologne, Germany. J Infect Dis.

[383] Maglione M., Pascarella A., Botti C. (2022). Changing epidemiology of acute viral respiratory infections in hospitalized children: the post-lockdown effect. Children Basel.

[384] Low Y.L., Wong S.Y., Lee E.K.H., Muhammed M.H. (2022). Prevalence of respiratory viruses among paediatric patients in acute respiratory illnesses in Malaysia. PLoS One.

[385] Lokida D., Farida H., Triasih R. (2022). Epidemiology of community-acquired pneumonia among hospitalised children in Indonesia: a multicentre, prospective study. BMJ Open.

[386] Lei C., Lou C.T., Io K. (2022). Viral etiology among children hospitalized for acute respiratory tract infections and its association with meteorological factors and air pollutants: a time-series study (2014-2017) in Macao. BMC Infect Dis.

[387] Kume Y., Hashimoto K., Shirato K. (2022). Epidemiological and clinical characteristics of infections with seasonal human coronavirus and respiratory syncytial virus in hospitalized children immediately before the coronavirus disease 2019 pandemic. J Infect Chemother.

[388] Kume Y., Hashimoto K., Chishiki M. (2022). Changes in virus detection in hospitalized children before and after the severe acute respiratory syndrome coronavirus 2 pandemic. Influenza Other Respir Viruses.

[389] Koul P.A., Saha S., Kaul K.A. (2022). Respiratory syncytial virus among children hospitalized with severe acute respiratory infection in Kashmir, a temperate region in northern India. J Glob Health.

[390] Kamata K., Thein K.N., Di Ja L. (2022). Clinical manifestations and outcome of viral acute lower respiratory infection in hospitalised children in Myanmar. BMC Infect Dis.

[391] Kafintu-Kwashie A.A., Nii-Trebi N.I., Obodai E., Neizer M., Adiku T.K., Odoom J.K. (2022). Molecular epidemiological surveillance of viral agents of acute lower respiratory tract infections in children in Accra, Ghana. BMC Pediatr.

[392] Jamieson N., Akande M., Karsies T., Smith R.M., Kline D., Spencer S.P. (2022). Respiratory pathogen detection in pediatric patients intubated for presumed infection. Pediatr Emerg Care.

[393] Hossain M.E., Rahman M.Z., Islam M.M. (2022). Pre COVID-19 molecular epidemiology of respiratory syncytial virus (RSV) among children in Bangladesh. Heliyon.

[394] Lamrani Hanchi A., Guennouni M., Ben Houmich T. (2022). Changes in the epidemiology of respiratory pathogens in children during the COVID-19 pandemic. Pathogens.

[395] Davis W., Duque J., Huang Q.S. (2022). Sensitivity and specificity of surveillance case definitions in detection of influenza and respiratory syncytial virus among hospitalized patients, New Zealand, 2012-2016. J Infect.

[396] Dananché C., Paranhos-Baccalà G., Messaoudi M. (2022). Nasopharyngeal viral and bacterial Co-Detection among children from Low- and middle-income countries with and without pneumonia. Am J Trop Med Hyg.

[397] Dai Y., Zhong J., Lan Y., Zhao X. (2022). Virus–virus interactions of febrile respiratory syndrome among patients in China based on surveillance data from February 2011 to December 2020. J Med Virol.

[398] Cui A., Xie Z., Xu J. (2022). Comparative analysis of the clinical and epidemiological characteristics of human influenza virus versus human respiratory syncytial virus versus human metapneumovirus infection in nine provinces of China during 2009-2021. J Med Virol.

[399] Ng D.C., Tan K.K., Ting G.S.S. (2022). Comparison of severe viral pneumonia caused by SARS-CoV-2 and other respiratory viruses among Malaysian children during the COVID-19 pandemic. Front Pediatr.

[400] Chawla K., Kumar A., Hegde A., Govindakarnavar A.K. (2022). A pilot study on aetiology of acute lower respiratory tract infections among children hospitalized of respiratory illness at a rural hospital in south coastal Karnataka. Biomed Pharmacol J.

[401] Chandy S., Manoharan A., Hameed A. (2022). A study on pediatric respiratory tract infections in hospitalised children from Chennai. Clin Epidemiol Glob Health.

[402] Cason C., Zamagni G., Cozzi G. (2022). Spread of respiratory pathogens during the COVID-19 pandemic among children in the Northeast of Italy. Front Microbiol.

[403] Bimouhen A., Regragui Z., El Falaki F. (2022). Viral aetiology of influenza-like illnesses and severe acute respiratory illnesses in Morocco, September 2014 to December 2016. J Glob Health.

[404] Ahmed A., Alsenaidy A.M., Mobaireek K.F., AlSaadi M.M. (2022). Viral etiology of acute respiratory infections during 2014-16 in Riyadh, Saudi Arabia. Future Virol.

[405] Shen Z., Zhang Y., Li H., Du L. (2022). Rapid typing diagnosis and clinical analysis of subtypes A and B of human respiratory syncytial virus in children. Virol J.

[406] Letafati A., Aghamirmohammadali F.S., Rahimi-Foroushani A., Hasani S.A., Mokhtari-Azad T., Yavarian J. (2022). No human respiratory syncytial virus but SARS-CoV-2 found in children under 5 years old referred to Children Medical Center in 2021, Tehran, Iran. J Med Virol.

[407] Calaor-Morin J., Arguelles V.L., Foronda J.L. (2022). Genotyping of respiratory syncytial virus among influenza-like illness and severe acute respiratory infection cases of children in the Philippines from 2006 to 2016. Influenza Other Respir Viruses.

[408] Agarwal A., Chakma N., Manchanda V., Dabas A. (2023). Virological profile of upper respiratory tract infections in children under 5 years of age-a cross sectional study in a tertiary care hospital in North India. Indian J Med Microbiol.

[409] Alaib H., Algariri N., Ahmed H. (2023). Frequency and seasonal variations of viruses causing respiratory tract infections in children pre-and post-COVID-19 pandemic in Riyadh (2017–2022). Cureus.

[410] Almeida T., Guimarães J.T., Rebelo S. (2023). Epidemiological changes in respiratory viral infections in children: the influence of the COVID-19 pandemic. Viruses.

[411] Alsayed A.R., Abed A., Abu-Samak M., Alshammari F., Alshammari B. (2023). Etiologies of acute bronchiolitis in children at risk for asthma, with emphasis on the human rhinovirus genotyping protocol. J Clin Med.

[412] Ciofi degli Atti M., Rizzo C., D'Amore C. (2023). Acute respiratory infection emergency access in a tertiary care children hospital in Italy, prior and after the SARS-CoV-2 emergence. Influenza Other Respir Viruses.

[413] DeJonge P.M., Monto A.S., Malosh R.E. (2023). Comparing the etiology of viral acute respiratory illnesses between children who do and do not attend childcare. Pediatr Infect Dis J.

[414] Edderdouri K., Kabbaj H., Laamara L. (2023). Contribution of the FilmArray BioFire® technology in the diagnosis of viral respiratory infections during the COVID-19 pandemic at Ibn Sina University Hospital Center in Rabat: epidemiological study about 503 cases. Adv Virol.

[415] Fourie E., Sijm Y.E., Badoux P. (2023). High detection rate of viral pathogens in nasal discharge in children aged 0 till 5 years. J Med Virol.

[416] Guo Y.-J., Wang B.-H., Li L., Li Y.-L., Chu X.-L., Li W. (2023). Epidemiological and genetic characteristics of respiratory syncytial virus infection in children from Hangzhou after the peak of COVID-19. J Clin Virol.

[417] Han X.-Y., Wang X.-L., Zhang J. (2023). Study on pathogen spectrum of 1,046 hospitalized children with respiratory tract infections during COVID-19. J Lab Med.

[418] Kandeel A., Fahim M., Deghedy O. (2023). Resurgence of influenza and respiratory syncytial virus in Egypt following two years of decline during the COVID-19 pandemic: outpatient clinic survey of infants and children, October 2022. BMC Public Health.

[419] Kang M., Sarkar S., Angurana S.K. (2023). Paradigm shift of respiratory viruses causing lower respiratory tract infection in children during COVID-19 pandemic in India. J Infect Dev Ctries.

[420] Kelly M.E., Gharpure R., Shivji S. (2023). Etiologies of influenza-like illness and severe acute respiratory infections in Tanzania, 2017–2019. PLOS Glob Public Health.

[421] Kişlal F., Hanilçe Y., Altaş B., Büyükbaşaran Z., Güven D. (2023). The disappearance of respiratory syncytial virus and influenza viruses in children during the second year of the COVID-19 pandemic-are non-pharmaceutical interventions as effective as vaccines?. Eur Rev Med Pharmacol Sci.

[422] Krumkamp R., Kohsar M., Nolte K. (2023). Pathogens associated with hospitalization due to acute lower respiratory tract infections in children in rural Ghana: a case–control study. Sci Rep.

[423] Kumar A., Bahal A., Singh L., Ninawe S., Grover N., Suman N. (2023). Utility of multiplex real-time PCR for diagnosing paediatric acute respiratory tract infection in a tertiary care hospital. Med J Armed Forces India.

[424] Kurskaya O.G., Prokopyeva E.A., Sobolev I.A. (2023). Changes in the etiology of acute respiratory infections among children in Novosibirsk, Russia, between 2019 and 2022: the impact of the SARS-coV-2 virus. Viruses.

[425] Li Y., Zhai Y., Lin Y. (2023). Epidemiology of respiratory syncytial virus in hospitalized children with community-acquired pneumonia in Guangzhou: a 10-year study. J Thorac Dis.

[426] Li M., Li C., Jian X., Han D., Zhao J., Jiang L. (2023). Viral etiology of acute respiratory tract infection among children under 5 years of age in Kunming City, China: a matched case–case–control study. J Appl Microbiol.

[427] Lin S.-C., Wang H.-C., Lin W.-C. (2023). Viral pneumonia during the COVID-19 pandemic, 2019–2021 evoking needs for SARS-CoV-2 and additional vaccinations. Vaccines.

[428] Mai W., Ren Y., Tian X. (2023). Comparison of common human respiratory pathogens among hospitalized children aged≤ 6 years in Hainan Island, China, during spring and early summer in 2019–2021. J Med Virol.

[429] Osborne C.M., Langelier C., Kamm J. (2024). Viral detection by reverse transcriptase polymerase chain reaction in upper respiratory tract and metagenomic RNA sequencing in lower respiratory tract in critically ill children with suspected lower respiratory tract infection. Pediatr Crit Care Med.

[430] Ramgopal S., Cotter J.M., Navanandan N. (2023). Viral detection is associated with severe disease in children with suspected community-acquired pneumonia. Pediatr Emerg Care.

[431] Samuels R.J., Sumah I., Alhasan F. (2023). Respiratory virus surveillance in hospitalized children less than two-years of age in Kenema, Sierra Leone during the COVID-19 pandemic (October 2020-October 2021). PLoS One.

[432] Shi T., Huang L. (2023). Prevalence of respiratory pathogens and risk of developing pneumonia under non-pharmaceutical interventions in Suzhou, China. Epidemiol Infect.

[433] Siddik A.B., Tanvir N.A., Bhuyan G.S. (2023). Bacterial and viral etiology of acute respiratory infection among the Forcibly Displaced Myanmar Nationals (FDMNs) in fragile settings in Cox's Bazar-a prospective case-control study. PLoS Negl Trop Dis.

[434] Steponavičienė A., Burokienė S., Ivaškevičienė I., Stacevičienė I., Vaičiūnienė D., Jankauskienė A. (2023). Influenza and respiratory syncytial virus infections in pediatric patients during the COVID-19 pandemic: a single-center experience. Child.

[435] Kohns Vasconcelos M., Meyer Sauteur P.M., Keitel K. (2023). Detection of mostly viral pathogens and high proportion of antibiotic treatment initiation in hospitalised children with community-acquired pneumonia in Switzerland-baseline findings from the first two years of the KIDS-STEP trial. Swiss Med Wkly.

[436] Virant M.J., Luštrek M., Kogoj R., Petrovec M., Uršič T. (2023). Changes in HRSV Epidemiology but not circulating variants in hospitalized children due to the emergence of SARS-CoV-2. Viruses.

[437] Wadilo F., Feleke A., Gebre M. (2023). Viral etiologies of lower respiratory tract infections in children< 5 years of age in Addis Ababa, Ethiopia: a prospective case–control study. Virol J.

[438] Xu D., Ji L., Wu X., Chen L. (2023). Molecular typing and epidemiological profiles of human respiratory syncytial virus infection among children with severe acute respiratory infection in Huzhou, China. J Clin Virol Plus.

[439] Yan Y., Sun J., Ji K. (2023). High incidence of the virus among respiratory pathogens in children with lower respiratory tract infection in northwestern China. J Med Virol.

[440] Yan Y., Wang D., Li Y. (2023). Prevalence, variation, and transmission patterns of human respiratory syncytial virus from pediatric patients in Hubei, China during 2020–2021. Virol Sin.

[441] Zarur-Torralvo S., Stand-Niño I., Flórez-García V., Mendoza H., Viana-Cárdenas E. (2023). Viruses responsible for acute respiratory infections before (2016–2019) and during (2021) circulation of the SARS-CoV-2 virus in pediatric patients in a reference center at Barranquilla Colombia: a pattern analysis. J Med Virol.

[442] Zdanowicz K., Lewandowski D., Majewski P. (2023). Clinical presentation and co-detection of respiratory pathogens in children under 5 years with Non-COVID-19 bacterial and viral respiratory tract infections: a prospective study in Białystok, Poland (2021-2022). Med Sci Monit.

[443] Zendehrouh M., Karimi A., Azimi L. (2023). Respiratory viral infections among children hospitalized in a great referral hospital in Iran during the coronavirus pandemic. Arch Pediatr Infect Dis.

[444] Zhang J., Yang T., Zou M., Wang L., Sai L. (2023). The epidemiological features of respiratory tract infection using the multiplex panels detection during COVID-19 pandemic in Shandong province, China. Sci Rep.

[445] Bimouhen A., Regragui Z., El Falaki F. (2023). Circulation patterns and molecular epidemiology of human respiratory syncytial virus over five consecutive seasons in Morocco. Influenza Other Respir Viruses.

[446] Hayek H., Amarin J.Z., Qwaider Y.Z. (2023). Co-detection of respiratory syncytial virus with other respiratory viruses across all age groups before and during the COVID-19 pandemic. Front Virol.

[447] Kandeel A., Fahim M., Deghedy O. (2023). Multicenter study to describe viral etiologies, clinical profiles, and outcomes of hospitalized children with severe acute respiratory infections, Egypt 2022. Sci Rep.

[448] Kubale J., Kujawski S., Chen I.R.A. (2023). Etiology of acute lower respiratory illness hospitalizations among infants in 4 countries. Open Forum Infect Dis.

[449] Morgan N., Buys H., Muloiwa R. (2023). RSV infection in children hospitalised with severe lower respiratory tract infection in a low-middle-income setting: a cross-sectional observational study. PLoS One.

[450] Naeem R., Albayati H., Insaaf A. (2023). Molecular detection of respiratory syncytial virus in infants and young children by the conventional reverse transcriptase polymerase chain reaction. J Popul Ther Clin Pharmacol.

[451] Rybak A., Cohen R., Kramer R. (2023). Respiratory syncytial virus in outpatient children with bronchiolitis: continuous virus circulation during the nonepidemic period. Pediatr Infect Dis J.

[452] Salim S., Celiloglu H., Tayyab F., Malik Z.A. (2023). Seasonal prevalence of respiratory pathogens among children in the United Arab Emirates: a multicenter cross-sectional study in the Pre-COVID-19 era. Cureus.

[453] Suh J.H., Ahn B., Song S.H. (2023). Etiology and clinical characteristics of community-acquired pneumonia in Korean children during the Pre-COVID-19 period, 2015-2020. J Korean Med Sci.

[454] Trang U.T.H., Phuong H.V.M., Hoang N.H. (2023). Circulation of human respiratory syncytial virus and new ON1 genotype in northern Viet Nam, 2017-2020. West Pa Surveill Response.

[455] Umar S., Yang R.Y., Wang X.Y., Liu Y.T., Ke P.F., Qin S. (2023). Molecular epidemiology and characteristics of respiratory syncytial virus among hospitalized children in Guangzhou, China. Virol J.

[456] Wadilo F., Feleke A., Gebre M. (2023). Viral etiologies of lower respiratory tract infections in children < 5 years of age in Addis Ababa, Ethiopia: a prospective case-control study. Virol J.

[457] Wanlapakorn N., Thongpan I., Sarawanangkoor N. (2023). Epidemiology and clinical characteristics of severe acute respiratory infections among hospitalized children under 5 years of age in a tertiary care center in Bangkok, Thailand, 2019-2020. Heliyon.

[458] Alimohammadi N., Aelami M.H., Pourbadakhshan N., Jamehdar S.A., Khadem-Rezaiyan M., Asli S. (2024). Viral etiologies of pneumonia and bronchiolitis in hospitalized children during the COVID-19 pandemic. Arch Pediatr Infect Dis.

[459] Altawalah H., Alfouzan W., Al-Fadalah T., Zalzala M.A., Ezzikouri S. (2024). Viral etiology of severe lower respiratory tract infections in SARS-CoV-2 negative hospitalized patients during the COVID-19 pandemic in Kuwait. Heliyon.

[460] Aneja S., Singh V., Narayan V.V. (2024). Respiratory viruses associated with severe acute respiratory infection in children aged <5 years at a tertiary care hospital in Delhi, India during 2013-15. J Glob Health.

[461] Begley K.M., Leis A.M., Petrie J.G. (2024). Epidemiology of respiratory syncytial virus (RSV) in adults and children with medically attended acute respiratory illness over three seasons. Clin Infect Dis.

[462] Bhardwaj S., Choudhary M.L., Chadha M.S. (2024). Resurgence of respiratory syncytial virus infection during COVID-19 pandemic in Pune, India. BMC Infect Dis.

[463] Buonsenso D., Ferro V., Viozzi F. (2024). Changes in clinical, demographic, and outcome patterns of children hospitalized with non-SARS-CoV-2 viral low respiratory tract infections before and during the COVID pandemic in Rome, Italy. Pediatr Pulmonol.

[464] Do L., Tsedenbal N., Khishigmunkh C. (2024). Respiratory syncytial virus and influenza infections in children in Ulaanbaatar, Mongolia, 2015–2021. Influenza Other Respir Viruses.

[465] Dorji K., Yuden P., Ghishing T.D. (2024). Respiratory syncytial virus among hospitalized patients of severe acute respiratory infection in Bhutan: cross-sectional study. Influenza Other Respir Viruses.

[466] Farzi R., Pirbonyeh N., Kadivar M.R., Moattari A. (2024). Prevalence of influenza viruses A and B, adenovirus, respiratory syncytial virus, and human metapneumonia viruses among children with acute respiratory tract infection. Adv Virol.

[467] Fröhlich G.C., Gregianini T.S., Pinheiro F.G. (2024). Resurgence of human respiratory syncytial virus during COVID-19 pandemic in Southern Brazil. J Med Virol.

[468] Hou M., Liu G., Meng C. (2024). Circulation patterns and molecular characteristics of respiratory syncytial virus among hospitalized children in Tianjin, China, before and during the COVID-19 pandemic (2017–2022). Virol Sin.

[469] Huang L.M., Xu Y.Y., Yang Y.Q. (2023). Molecular epidemiology and clinical characteristics of respiratory syncytial virus in hospitalized children during winter 2021-2022 in Bengbu, China. Front Public Health.

[470] Korsun N., Trifonova I., Madzharova I. (2024). Resurgence of respiratory syncytial virus with dominance of RSV-B during the 2022–2023 season. Front Microbiol.

[471] Lebreiro G.P., Venceslau M.T., Guimarães M.A.A.M. (2024). Respiratory syncytial virus infection in children during SARS-CoV-2 pandemic at a referral center in Rio de Janeiro, Brazil. J Bras Pneumol.

[472] Leija-Martínez J.J., Cadena-Mota S., González-Ortiz A.M. (2024). Respiratory syncytial virus and other respiratory viruses in hospitalized infants during the 2023–2024 winter season in Mexico. Viruses.

[473] Li H.M., Yang Y., Tao R., Shang S.Q. (2024). Analyzing infections caused by 11 respiratory pathogens in children: pre- and post-COVID-19 pandemic trends in China. J Med Virol.

[474] Li M., Cong B., Wei X. (2024). Characterising the changes in RSV epidemiology in Beijing, China during 2015-2023: results from a prospective, multi-centre, hospital-based surveillance and serology study. Lancet Reg Health West Pac.

[475] Li S.L., Xue Z.F., Feng Y.X. (2024). Epidemiological characteristics of eleven common respiratory viral infections in children. BMC Pediatr.

[476] Liu S., Lei Y., Chen X.X., Wen Z.H., Mei B. (2024). Epidemiological characteristics of respiratory pathogens infections among children after the removal of non-pharmaceutical interventions in central China. Virol J.

[477] Liu X.K., Xu M.Y., Bai M.J. (2024). Epidemiology of respiratory syncytial virus among all ages in 2023 in Beijing, China. Adv Public Health.

[478] Lv G.J., Shi L.M., Liu Y., Sun X.C., Mu K. (2024). Epidemiological characteristics of common respiratory pathogens in children. Sci Rep.

[479] Ma R., Liu Z.B., Zhang L. (2024). Epidemiological characteristics of severe community-acquired pneumonia in children admitted to two tertiary hospitals in Shihezi, Xinjiang Region, China in 2023: a cross-sectional analysis. J Thorac Dis.

[480] Meier K., Riepl A., Voitl P. (2024). Characterisation of RSV infections in children without chronic diseases aged 0–36 months during the post-COVID-19 winter season 2022/2023. Front Pediatr.

[481] Menezes R.C., Ferreira I.B.B., Sobral L. (2024). Severe viral lower respiratory tract infections in Brazilian children: clinical features of a national cohort. J Infect Public Health.

[482] Mojarrad S., Tavakoli Movaghar N., Edalat F., Letafati A., Kargar Jahromi Z., Moattari A. (2024). Epidemiological and phylogenetic assessment of human respiratory syncytial virus among pediatric patients presenting acute respiratory infections in Shiraz, Iran during 2015-2016. Iran J Microbiol.

[483] Moyes J., Tempia S., Walaza S. (2024). Risk factors for severe respiratory syncytial virus-associated respiratory tract infection in a high HIV prevalence setting, South Africa, 2012 – 2018. BMC Infect Dis.

[484] Ndiaye D., Diatta G., Bassene H. (2024). Prevalence of respiratory pathogens in nasopharyngeal swabs of febrile patients with or without respiratory symptoms in the niakhar area of rural Senegal. Pathogens.

[485] Pan L., Yuan Y., Cui Q. (2024). Impact of the COVID-19 pandemic on the prevalence of respiratory viral pathogens in patients with acute respiratory infection in Shanghai, China. Front Public Health.

[486] Pasittungkul S., Thongpan I., Vichaiwattana P. (2024). Prevalence and genetic diversity of respiratory syncytial virus reinfections in young Thai children, 2016–2023. J Med Virol.

[487] Pérez-Camacho P., Monsalve A.M., Torres-Canchala L. (2024). Severe community-acquired pneumonia in pediatric patients at a high-complexity center in Cali, Colombia. Infectio.

[488] Philomenadin F.S., Mohammed S., Jayagandan S. (2024). Characterizing human respiratory syncytial virus among children admitted with acute respiratory tract infections from 2019 to 2022. J Med Virol.

[489] Pun J.C.S., Tao K.P., Yam S.L.S. (2024). Respiratory viral infection patterns in hospitalised children before and after COVID-19 in Hong Kong. Viruses.

[490] Ramzali M., Salimi V., Cheraghali F. (2024). Epidemiology and clinical features of respiratory syncytial virus (RSV) infection in hospitalized children during the COVID-19 pandemic in Gorgan, Iran. Health Sci Rep.

[491] Reddy B., Simane A., Mthiyane H., Mashishi B., Mbenenge N., Treurnicht F.K. (2024). Prevalence and seasonal patterns of 16 common viral respiratory pathogens during the COVID-19 pandemic in Gauteng Province, South Africa, 2020–2021. Viruses.

[492] Reller M.E., Mehta K., McCollum E.D. (2024). Viral acute lower respiratory tract infections (ALRI) in rural Bangladeshi children prior to the COVID-19 pandemic. Influenza Other Respir Viruses.

[493] Rojo-Alba S., Martínez Z.P., González-Alba J.M. (2024). Respiratory syncytial virus incidence and typing in the last six seasons in the north of Spain (Asturias). Genetic characterization during the SARS-CoV-2 pandemic. J Med Virol.

[494] Shrestha S.K., Shrestha J., Shrestha B. (2024). Enteroviruses, respiratory syncytial virus and seasonal coronaviruses in influenza-like illness cases in Nepal. Microbiol Res.

[495] Simusika P., Okamoto M., Dapat C. (2024). Characterization of human respiratory syncytial virus in children with severe acute respiratory infection before and during the COVID-19 pandemic. IJID Reg.

[496] Stacevičienė I., Ivaškevičienė I., Burokienė S. (2024). Epidemiological changes of acute respiratory infections in children: a single-center experience after COVID-19 lockdown. PLoS One.

[497] Sun B.H., Qiu Y.Z., Wang L.L. (2024). Viral etiology of febrile respiratory syndrome among patients in Liaoning Province, China. BMC Infect Dis.

[498] Tayachew A., Teka G., Gebeyehu A. (2024). Prevalence of respiratory syncytial virus infection and associated factors in children aged under five years with severe acute respiratory illness and influenza-like illness in Ethiopia. IJID Reg.

[499] Tran X.D., Hoang V.T., Goumballa N. (2024). Viral and bacterial microorganisms in Vietnamese children with severe and non-severe pneumonia. Sci Rep.

[500] Umran N., Kalpana S., Dhandapani P. (2024). Prevalence of non- SARS CoV-2 respiratory virus infection in children during COVID-19 pandemic in Chennai, South India. Indian J Pathol Microbiol.

[501] Wei M.Y., Li S.S., Lu X.H., Hu K.M., Li Z.L., Li M. (2024). Changing respiratory pathogens infection patterns after COVID-19 pandemic in Shanghai, China. J Med Virol.

[502] Wu Y.D., Zhou J., Shu T., Li W., Shang S.Q., Du L.Z. (2024). Epidemiological study of post-pandemic pediatric common respiratory pathogens using multiplex detection. Virol J.

[503] Xu X., Zhang Y., Xu L., Jiang W., Hao C. (2024). Analysis of respiratory pathogen detection in hospitalized children with acute respiratory tract infections after ending the zero COVID policy. Sci Rep.

[504] Yang R.L., Xu H.M., Zhang Z.Z. (2024). The epidemiology of pathogens in community-acquired pneumonia among children in Southwest China before, during and after COVID-19 non-Pharmaceutical interventions: a cross-sectional study. Influenza Other Respir Viruses.

[505] Yang T.D., Lian H.B., Liao J.Y. (2024). Epidemiological characteristics and meteorological factors of acute respiratory infections (ARIs) in hospitalized children in eastern Guangdong, China. Sci Rep.

[506] Zhang G.Q., Zhang Y., Ba L.M. (2024). Epidemiological changes in respiratory pathogen transmission among children with acute respiratory infections during the COVID-19 pandemic in Kunming, China. BMC Infect Dis.

[507] Zhang L., Wang Y.P., Xu H.M. (2024). Prevalence of respiratory viruses in children with acute respiratory infections in Shanghai, China, from 2013 to 2022. Influenza Other Respir Viruses.

[508] Zhao P., Zhang Y., Wang J. (2024). Epidemiology of respiratory pathogens in patients with acute respiratory infections during the COVID-19 pandemic and after easing of COVID-19 restrictions. Microbiol Spectr.

[509] Zhao Q.W., Ke P.F., Hu L.S. (2024). Epidemiological characteristics of upper respiratory tract pathogens in children in Guangdong, China. Clin Respir J.

[510] Zheng P.P., Zhao Y.N., Wang Z.K. (2024). Prevalence of respiratory pathogens among hospitalised patients with acute respiratory infection during and after the COVID-19 pandemic in Shijiazhuang, China. Front Cell Infect Microbiol.

[511] Adu-Gyamfi C., Asamoah J.A., Frimpong J.O. (2025). Prevalence of common respiratory viruses in children: insights from post-pandemic surveillance. BMC Infect Dis.

[512] Bandeira T.D.J.P.G., Oliveira A.L.S.D., Martins L.F.P. (2025). Molecular detection of respiratory viruses: an observational study on respiratory co-infections in children and adults. Braz J Microbiol.

[513] Burrell R., Saravanos G.L., Kesson A. (2025). Respiratory virus detections in children presenting to an Australian paediatric referral hospital pre-COVID-19 pandemic, January 2014 to December 2019. PLoS One.

[514] Cha M., Zhang Q., Luo M.J. (2025). Epidemiological characteristics of Mycoplasma pneumoniae and viral infections in hospitalized children with recurrent lower respiratory tract infections. Am J Transl Res.

[515] Correia W., Dorta-Guerra R., Sanches M., Valladares B., de Pina-Araújo I.I.M., Carmelo E. (2025). Epidemiological and clinical profile of viral respiratory infections in children under 5 years at pre- and post-COVID-19 era in Praia, Cabo Verde. Trop Med Int Health.

[516] Han T.Y., Wang Y.J., Zhang D. (2025). Changes in infant respiratory pathogens pre-during, and post-COVID-19 non-pharmacological interventions in Beijing. Ital J Pediatr.

[517] Jamalidoust M., Pouladfar G., Eskandari M. (2024). Etiology of severe acute respiratory infections in ICU-admitted patients during the COVID-19 pandemic in Iran: a single center study. Arch Pediatr Infect Dis.

[518] Jiang M., Wang F., Xu Y. (2025). The changed endemic pattern of respiratory syncytial virus infections in pediatric patients with the relaxation of NPIs in Beijing, China, 2022−2023. J Med Virol.

[519] Jiang Y., Lu J.Y., Tan Z.R. (2025). Epidemiological characteristics of acute viral and mycoplasma respiratory infections in Yongzhou, China: a retrospective descriptive study. Front Public Health.

[520] Jie W., Wenyan H., Chang L. (2025). Epidemiological intricacies of respiratory pathogens: a single-center study on infection dynamics in Beijing, 2023–2024. Front Public Health.

[521] Khan T., Halder S., Das R.S. (2025). Molecular epidemiology of influenza, respiratory syncytial virus, SARS-CoV-2, other respiratory viruses and bacteria among children 0–2-year-olds in West Bengal: a one-year influenza-like illness surveillance study (2022–2023). Front Epidemiol.

[522] Lai Q.R., Chu X.L., Chen Y.Y., Li W., Guo Y.J., Shang S.Q. (2025). Epidemiological and clinical characteristics of respiratory syncytial virus infection in children in Hangzhou (2022–2023). Pathogens.

[523] Li J., Zhang H., Xu Q., Huang Z. (2025). Analysis of the results of 13 combined pathogen detection in 3966 hospitalised children with acute lower respiratory tract infection. Sci Rep.

[524] Liu P.C., Xu M.H., Lu L.J. (2025). Resurgence of common respiratory viruses and mycoplasma pneumoniae after ending the zero-COVID policy in Shanghai. Sci Rep.

[525] Ma H., Hou J., Wang J., Yuan Z., Man S. (2025). Analysis of common respiratory pathogens and epidemiological trends during peak influenza seasons in Tengzhou. BMC Infect Dis.

[526] Matache Vasilache E.R., Gurau G., Raileanu C.R. (2025). Pathogen profile of children hospitalised with viral respiratory infections in Galati county, Romania. Viruses.

[527] Matsumura Y., Yamamoto M., Tsuda Y. (2025). Epidemiology of respiratory viruses according to age group, 2023–24 winter season, Kyoto, Japan. Sci Rep.

[528] Mi S., Yang Y., Li T. (2025). Epidemiological changes of common respiratory viruses in Shanghai, during 2021–2023. Clin Epidemiol Glob Health.

[529] Moleleki M., Reddy C., Ndlangisa K. (2025). Respiratory pathogens detected in children aged <5 years hospitalized with severe respiratory illness, South Africa, 2017. Front Pediatr.

[530] Pale M., Tivane A., Gils T. (2025). Risk factors and circulation pattern of respiratory syncytial virus in children under 2 years in Maputo, Mozambique. Int Health.

[531] Sadeghi R.H., Pourakbari B., Mahmoudi S. (2025). Evaluation of common respiratory viruses other than SARS-CoV-2 in hospitalized children during the COVID-19 pandemic. BMC Infect Dis.

[532] Santos R.W.F., Pimentel A.S., de Jesus M.C.S. (2025). Prevalence of respiratory viruses in children in northeast Brazil: the scenario before the COVID-19 pandemic. IJID Reg.

[533] Shrestha S., Bijukchhe S.M., Wahl B. (2025). Respiratory viral detection in children hospitalized with pneumonia during periods of major population disruptions in Nepal, 2014-2018. J Pediatric Infect Dis Soc.

[534] Soares T.D.N., Andrade N.C.O., Santos S. (2025). Risk of incidence and lethality by etiology of severe acute respiratory syndrome in hospitalized children under 1 year of age in Brazil in 2024: a cross-sectional study. Trop Med Infect Dis.

[535] Takashita E., Shimizu K., Kawakami C. (2025). Impact of COVID-19 on respiratory virus infections in children, Japan, 2018–2023. Immun Inflamm Dis.

[536] Tan J., Chen Y., Lu J. (2025). Pathogen distribution and infection patterns in pediatric severe pneumonia: a targeted next-generation sequencing study. Clin Chim Acta.

[537] Tang Z., Fan H.H., Tian Y.L., Lv Q.S. (2025). Epidemiological characteristics of six common respiratory pathogen infections in children. Microbiol Spectr.

[538] Tayachew A., Mekuria Z., Shure W. (2025). Epidemiology of respiratory syncytial virus and its subtypes among cases of influenza like illness and severe acute respiratory infection: findings from nationwide sentinel surveillance in Ethiopia. BMC Infect Dis.

[539] Wang H.P., Guo Y.P., Wang R.J. (2025). Epidemiological shifts in children respiratory pathogens in Shenzhen, China: a comparative analysis before and after the relaxation of COVID-19 non-pharmaceutical interventions. Influenza Other Respir Viruses.

[540] Wang J., Han Y.L., Wei X.L., Liu Y.Q., Zhang H., Ma X. (2025). The impact of COVID-19 pandemic on the etiological spectrum of respiratory infections in children. Eur J Clin Microbiol Infect Dis.

[541] Wu Z., Ye C., Wang G. (2025). Seasonal patterns and prevalence of respiratory pathogens in children with acute respiratory infections in Wuhan, China. J Infect Dev Ctries.

[542] Xu X.C., Chen X.Y., He J. (2025). Epidemiological changes in hospitalized bronchiolitis in children under 2 years of age in Hangzhou before and after COVID-19 restriction easing. Infect Drug Resist.

[543] Xu X.Y., Wu L.L., Liu Y. (2025). Impact of the COVID-19 pandemic on the respiratory syncytial virus infections in children admitted with community acquired pneumonia: a retrospective study at a tertiary hospital of Northeast China. Eur J Clin Microbiol Infect Dis.

[544] Zeng Y.J., Wang G.W., Yang H.C. (2025). Estimating the prevalence of six common respiratory viral infections in Zhangzhou, China using nasopharyngeal swabs in adults and throat swabs in children. Sci Rep.

[545] Zhang L., Wang Y.H., Zheng Y. (2025). Epidemiological characteristics of pathogens in bronchoalveolar lavage fluid in children with lower respiratory tract infections: a retrospective analysis. Pediatr Pulmonol.

[546] Zhu J.W., Wu S.Q., Chen Y., Zheng L.P. (2025). Prevalence and distribution of respiratory pathogens in pediatric acute respiratory infections in Putian, China. BMC Infect Dis.

