# Supplementary material for: The prevalence and role of human respiratory syncytial virus in pediatric respiratory tract infections: a systematic review and meta-analysis of global data
Source: eClinicalMedicine. 2026 Mar 20;94:103837. doi: 10.1016/j.eclinm.2026.103837 (PMC13018908; doi:10.1016/j.eclinm.2026.103837)
Supplement: Supplementary Table [file mmc1.docx]

**Table 1.** The full details of the search strategy for each database.

| **Database** | **Search terms** |
| --- | --- |
| **PubMed** | (("Respiratory Syncytial Virus*"[Title/Abstract] OR RSV[Title/Abstract] OR hRSV[Title/Abstract] OR "Human respiratory syncytial virus"[Title/Abstract] OR "Human orthopneumovirus"[Title/Abstract]) AND (Pediatric[Title/Abstract] OR Pediatrics[Title/Abstract] OR Child[Title/Abstract] OR Children[Title/Abstract] OR Infant[Title/Abstract] OR Newborn[Title/Abstract] OR Neonate[Title/Abstract] OR Adolescent[Title/Abstract]))) |
| **Scopus** | TITLE-ABS-KEY ( ( "Respiratory Syncytial Virus*" OR rsv OR hrsv OR "human respiratory syncytial virus" OR "Human orthopneumovirus" ) AND ( pediatric OR pediatrics OR child OR children OR infant OR newborn OR neonate OR adolescent ) ) |
| **Web of science** | (TS=((“Respiratory Syncytial Virus*” OR RSV OR hRSV OR “human respiratory syncytial virus” OR “Human orthopneumovirus”) AND (pediatric OR pediatrics OR child OR children OR infant OR newborn OR neonate OR adolescent))) |

**Meta-regression**

Meta-regression analyses were performed to explore potential sources of heterogeneity in both prevalence estimates and the association between the RSV virus and disease. In the prevalence meta-analysis, larger sample sizes were associated with slightly lower prevalence, and both disease type and sample type significantly influenced the estimates, whereas economic classification, study design, and sampling time had no significant effect. For the association meta-analysis of 33 case–control studies, none of the study-level characteristics, including sample size, country economic classification, sample type, disease type, and sampling time, were significantly associated with the observed effect sizes, and the joint test for all covariates was non-significant. These results indicate that study characteristics partially explain heterogeneity in prevalence but do not account for the variability in effect sizes for the association between the virus and disease.
